# Synergistic and Dose-Controlled Regulation of Cellulase Gene Expression in *Penicillium oxalicum*

**DOI:** 10.1371/journal.pgen.1005509

**Published:** 2015-09-11

**Authors:** Zhonghai Li, Guangshan Yao, Ruimei Wu, Liwei Gao, Qinbiao Kan, Meng Liu, Piao Yang, Guodong Liu, Yuqi Qin, Xin Song, Yaohua Zhong, Xu Fang, Yinbo Qu

**Affiliations:** 1 State Key Laboratory of Microbial Technology, School of Life Science, Shandong University, Jinan, Shandong, China; 2 National Glycoengineering Research Center, Shandong University, Jinan, Shandong, China; University of California, UNITED STATES

## Abstract

Filamentous fungus *Penicillium oxalicum* produces diverse lignocellulolytic enzymes, which are regulated by the combinations of many transcription factors. Here, a single-gene disruptant library for 470 transcription factors was constructed and systematically screened for cellulase production. Twenty transcription factors (including ClrB, CreA, XlnR, Ace1, AmyR, and 15 unknown proteins) were identified to play putative roles in the activation or repression of cellulase synthesis. Most of these regulators have not been characterized in any fungi before. We identified the ClrB, CreA, XlnR, and AmyR transcription factors as critical dose-dependent regulators of cellulase expression, the core regulons of which were identified by analyzing several transcriptomes and/or secretomes. Synergistic and additive modes of combinatorial control of each cellulase gene by these regulatory factors were achieved, and cellulase expression was fine-tuned in a proper and controlled manner. With one of these targets, the expression of the major intracellular β-glucosidase Bgl2 was found to be dependent on ClrB. The Bgl2-deficient background resulted in a substantial gene activation by ClrB and proved to be closely correlated with the relief of repression mediated by CreA and AmyR during cellulase induction. Our results also signify that probing the synergistic and dose-controlled regulation mechanisms of cellulolytic regulators and using it for reconstruction of expression regulation network (RERN) may be a promising strategy for cellulolytic fungi to develop enzyme hyper-producers. Based on our data, ClrB was identified as focal point for the synergistic activation regulation of cellulase expression by integrating cellulolytic regulators and their target genes, which refined our understanding of transcriptional-regulatory network as a “seesaw model” in which the coordinated regulation of cellulolytic genes is established by counteracting activators and repressors.

## Author Summary

Cellulolytic fungi have evolved into sophisticated lignocellulolytic systems to adapt to their natural habitat. This trait is important for filamentous fungi, which are the main source of cellulases utilized to degrade lignocellulose to fermentable sugars. *Penicillium oxalicum*, which produces lignocellulolytic enzymes with more diverse components than *Trichoderma reesei*, has the capacity to secrete large amounts of cellulases. Meanwhile, cellulase expression is regulated by a complex network involved in many transcription factors in this organism. To better understand how cellulase genes are systematically regulated in *P*. *oxalicum*, we employed molecular genetics to uncover the cellulolytic transcription factors on a genome-wide scale. We discovered the synergistic and tunable regulation of cellulase expression by integrating cellulolytic regulators and their target genes, which refined our understanding of transcriptional-regulatory network as a “seesaw model” in which the coordinated regulation of cellulolytic genes is established by counteracting activators and repressors.

## Introduction

Cellulolytic fungi have an inherent characteristic of cellulose deconstruction and can be used for bioconversion of insoluble plant cell wall polysaccharides into fermentable sugars [1–3]. The highly efficient production of their extracellular hydrolytic enzymes and other synergistic proteins [2,4], such as swollenin [5], plays a key role in reducing the cost of the biorefinery process [4]. However, incomplete knowledge of transcriptional regulatory networks for cellulolytic fungi has hampered the systematic improvement of cellulase production. These cellulolytic system genes are coordinately but differentially regulated in various cellulase producers [2,6]. Further characterization and manipulation of the cellulase regulatory network’s components will allow the rational engineering of cellulolytic fungi for improved enzyme production.

Transcriptional regulation of cellulolytic gene expression is central in controlling the carbohydrate hydrolysis process [6], and several positive or negative transcriptional factors of these degradative pathways were identified, such as the regulators encoded by the *creA*/*cre1*/*cre-1* [7–9], *xyr1*/*xlnr*/*xlr-1* [10–12], *aceI* [13], *aceII* [14], *ace3* [15], *clrB*/*clr-2*/*manR* [16,17], and *bglR* [18] genes. The overexpression of these activators or deletion of some repressors is efficient in enhancing the cellulase and hemicellulase expressions [19,20]. However, the degree of cellulase induction differentially responds to these diverse regulator abundances. The transcription factor CreA, an ortholog of Migl from *Saccharomyces cerevisiae* [21], is a pivotal regulator mediating carbon catabolite repression (CCR) in filamentous fungi [7–9], and its deletion results in the obvious increase of cellulase expression and secretion. A transcriptional regulatory cascade that controls the xylanolytic genes between CreA and XlnR is also built in *Aspergillus niger* in response to preferred carbon sources [22]. In addition, the *cre1* deletion mutant shows a conidiation formation defect [23]. Two novel zinc binuclear cluster transcription factors (CLR-1 and CLR-2) required for growth and enzymatic activity on cellulose were identified in *Neurospora crassa* [17]. The constitutive expression of *clr-2* by the control of the promoter from *ccg-1* is sufficient to drive cellulase gene expression when cultures are subjected to starvation [20]. In addition, the β-glucosidase regulator BglR and cellulase expression activator AceII were identified in *Trichoderma reesei* [18], but their orthologous encoding genes were absent in the *Penicillium oxalicum* genome [24]. Currently, the abilities to tune the expression abundance of just one transcription factor, as noted above, have profound effects on cellulase expression in these cellulolytic fungi [18–20,25]. However, whether such a simple mechanism could operate in the context of cellulolytic regulator combinations, including these characterized and novel transcription factors, remains unclear.

The *P*. *oxalicum* wild-type strain 114–2 was isolated from the soil in China more than 30 years ago [26]. A partially derepressed mutant JU-A10, which shows cellulolytic activity that is more than three times higher than that of its parent strain 114–2, was obtained after many rounds of mutagenesis and screening [26]. The mutant JU-A10 was further mutated to a cellulase hyper-producer JU-A10-T [26] and has been utilized in industrial processes for years. The clear genetic background provided by genome sequencing facilitated the rational improvement of these strains to enhance the expression of cellulolytic enzymes [24]. Currently, several structural genes associated with cellulase expression have been studied. The deletion of gene *bgl2* (encoding the major intracellular β-glucosidase) [27] or PDE_01641 (the ortholog of *N*. *crassa* NCU05137) [28] results in the increase of cellulase production in *P*. *oxalicum*. In addition, three cellodextrin transporters (CdtC, CdtD, and CdtG) were identified, and their overexpression obviously increases the extracellular cellobiohydrolase activities [29]. However, evidence from diverse cellulolytic fungi showed that engineering cellulolytic transcription factors might have more efficacy in upregulating cellulase expression than merely manipulating the expression of structural genes for the major cellulases [1,19,20,30]. Subsequent studies to identify several specific regulators and their roles in regulating cellulase gene expression were conceptually appealing in cellulolytic fungi.

In this study, twenty transcription factors putatively involved in cellulase expression pathways were identified from a single-gene disruptant library. The single overexpression or deletion of these genes triggered cellulase expression to varying degrees, and synergistic and tunable cellulase expressions were observed in the combinations of the identified individual transcription factors. Furthermore, the responsiveness of the induction of cellulase expression by activator and relief from potential carbon catabolite repression to the internal signal cascades by the lack of the major intracellular β-glucosidase Bgl2 was also well-established. The suggested mechanisms of synergistic effects on cellulase expression might be general properties in cellulolytic fungi and broadly enable engineering strategies for the protein hyper-producers.

## Results

### Screening for novel transcription factors involved in *P*. *oxalicum* cellulose deconstruction

To decipher the transcriptional-regulatory network that governs cellulase expression in *P*. *oxalicum*, we first sought to identify the transcription factors (TF) that play roles in cellulolytic gene expression systematically. A total of 522 genes encoding sequence-specific regulators were predicted according to the protein sequence domain [24]. For the amplification of the flanking sequences of these TF-disrupting cassettes, primers were designed to meet the following criteria: GC-content 45%–60%, Tm: 50°C –60°C, and a length of 20 base pairs. The lengths of the 5’ and 3’ flanking regions ranged from 1.0 kb to 1.5 kb for each gene. The chimeric primers (S1 Table) for the amplification of upstream and downstream flanking fragments carried 25 bases of homologous sequence overlapping with the ends of *ptra* marker sequence [31]. A final fragment that contains target gene flanking sequences surrounding *ptra* was created by double-joint PCR [32] and transformed into the *P*. *oxalicum* Δ*pku70* mutant via protoplast transformation [33]. Pku70 and its homologs are involved in the non-homologous end joining (NHEJ) repair of double-strand breaks in diverse eukaryotes [32]. Considering the high homologous recombination frequency in the *pku70* mutant (NHEJ-deficient background) [33], we selected three colonies per gene from these resulting transformants. The conidia from these primary transformants were purified by repeating the mono-spore isolation twice on the pyrithiamine resistance plates to obtain homokaryotic knockout mutants. The transcription factor gene replacement with *ptra* was verified by PCR-based screening. We found that the use of unpurified final amplicon of deletion cassettes resulted in almost 90% success in deleting the targeting genes in the Δ*pku70* mutant. Finally, a transcription factor mutant set, which bears a single deletion for 470 transcription factor genes in *P*. *oxalicum*, was successfully constructed.

The transcription factor deletion strains were screened and initially characterized for cellulose deconstruction on cellulose plates. According to the halos produced by the transformants on cellulose plates, 20 transcription factors that displayed putative roles in cellulase production were identified (Table 1). Twelve deletion strains exhibited increased cellulase activities and eight deletion strains exhibited decreased cellulase activities. These transcription factors represented negative and positive regulators of cellulose deconstruction, respectively. None of these transcription factors has been well characterized at the molecular level in *P*. *oxalicum*.

**Table 1:** Transcription factor genes affecting cellulase and xylanase production.

*P*. *oxalicum* Gene ID	Pfam families	Function	*N*. *crassa* Gene ID	E value	*T*. *reesei* Gene ID	E value	Name	Cellulase activity
PDE_05999	Zn_clus; Fungal_trans	Cellulose degradation regulator-2	NCU08042	e-131	26163	e-116	ClrB	----
PDE_05883	Zn_clus; Fungal_trans	Cellulose degradation regulator-2	NCU08042	5.00E-96	26163	2.00E-96	ClrB-2	---
PDE_03168	zf-H2C2_2	Carbon catabolite regulation	NCU08807	e-111	120117	e-111	CreA	++++
PDE_01988	zf-H2C2	A repressor of cellulase and xylanase expression	NCU09333	4.00E-98	75418	1.00E-93	Ace1	++
PDE_07674	Zn_clus; Fungal_trans	Xylan degradation regulator-1	NCU06971	0	122208	0	XlnR	--
PDE_03964	Zn_clus; Fungal_trans	Starch degradation regulator	NCU07788	2.00E-38	55105	9.00E-95	AmyR	+++
PDE_00153	Copper-fist	Uncharacterized	ND	—	122767	3.00E-32	—	+
PDE_07199	Homeobox	Hypothetical protein	NCU03070	5.00E-19	121074	5.00E-15	—	++
PDE_02584	zf-H2C2_2	C2H2 type zinc finger domain-containing protein	NCU07952	e-116	36391	e-123	—	+
PDE_09881	Zn2Cys6	Short aerial hyphae-3	NCU07535	6.00E-44	123445	4.00E-49	—	+
PDE_01512	Bromodomain	Porin-binding domain protein-1	NCU02354	e-179	62493	0	—	+
PDE_07134	zf-C2H2_4	Conidial separation-1	NCU02713	6.00E-23	120597	1.00E-21	—	+
PDE_04095	KilA-N	Ascospore maturation-1	NCU01414	e-132	74531	e-124	StuA	++
PDE_09961	HSF_DNA-bind	Heat-shock transcription factor-1	NCU08512	e-109	78688	e-107	—	-
PDE_09023	bZIP_1	Uncharacterized	NCU04058	2.00E-29	65315	1.00E-30	—	--
PDE_01706	zf-C2H2_4	Uncharacterized	ND	—	ND	—	—	-
PDE_08372	zf-H2C2_2	Developmental regulation	NCU03043	3.00E-66	58011	1.00E-66	FlbC	-
PDE_08462	zf-H2C2_2	Uncharacterized	NCU03421	5.00E-46	38080	2.00E-40	—	--
PDE_00988	Bromodomain	Uncharacterized	NCU02078	7.00E-47	122628	6.00E-44	—	+
PDE_03268	Zn_clus; Fungal_trans	Uncharacterized	NCU01478	4.00E-49	59760	6.00E-81	—	+

"ND" means that no homolog was detected.

"+/-" means the value for cellulase activity up-regulated or downregulated standard deviation in a strain deleting the gene.

**Fig 1:**
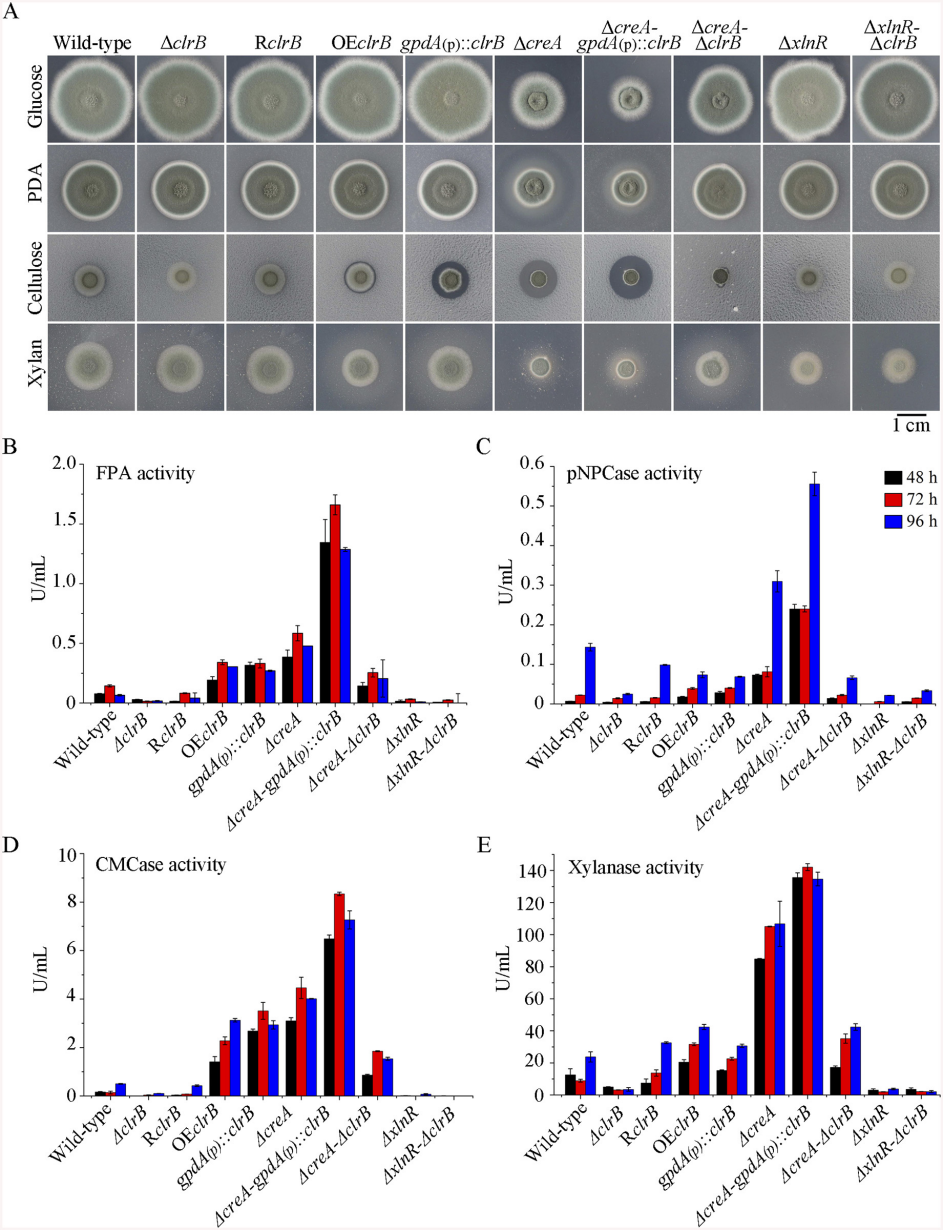
Phenotypes on different carbon sources and enzyme activities of culture supernatants from mutants containing *clrB*, *xlnR* and/or *creA* mutations. (A) The phenotypes for Δ*clrB*, R*clrB*, OE*clrB*, *gpdA*(p)::*clrB*, Δ*creA*, Δ*creA-gpdA*(p)::*clrB*, Δ*creA*-Δ*clrB*, Δ*xlnR*, and Δ*xlnR*-Δ*clrB* mutants versus wild-type strain grown on glucose, PDA, xylan or cellulose plates for 5 days. OE*clrB*: overexpression of ClrB by *clrB* native promoter; *gpdA*(p)::*clrB*: overexpression of ClrB by *A*. *nidulans gpdA* promoter. (B-E) Comparison of FPA, pNPCase activity, CMCase activity and xylanase activity from 48-h to 96-h old culture supernatants from Δ*clrB*, R*clrB*, OE*clrB*, *gpdA*(p)::*clrB*, Δ*creA*, Δ*creA-gpdA*(p)::*clrB*, Δ*creA*-Δ*clrB*, Δ*xlnR*, and Δ*xlnR*-Δ*clrB* mutants versus wild-type strain grown in 2% cellulose as a sole carbon source.

Among these transcription factor genes, PDE_05999, PDE_03168, PDE_07674 and PDE_03964 were previously known and encoded as ClrB, CreA, XlnR and AmyR regulators, respectively. The strongest effects on cellulose deconstruction observed in Δ*clrB*, Δ*creA*, Δ*xlnR* and Δ*amyR* mutants indicated that these three genes encode the major regulators of some lignocellulolytic enzymes (Fig 1A). To clarify the mechanisms of lignocellulose deconstruction in *P*. *oxalicum*, we initially focused on the characterization of these central lignocellulolytic regulators ClrB, CreA, XlnR, and AmyR, and then identified their target genes involved in plant cell wall deconstruction. Up to date, mating assays in *P*. *oxalicum* have not been performed to remove the *pku70* deletion through the crossing approach as *T*. *reesei* [34] and *N*. *crassa* [35] strains. We therefore constructed the corresponding mutants in the wild-type strain through the conventional transformation approach. Genomic DNA from putative transformants was analyzed by q-PCR (quantitative-PCR) and/or Southern blot (S1 Fig), and these transformants in which a single copy integration at the only a transcription factor gene locus were selected and further characterized.

### Transcriptional regulation of cellulase genes by ClrB and the characterization of ClrB regulon

The function of *clrB* (PDE_05999) was identified independently in our lab. The regulator protein sequence has 39% (BioEdit, E Value = 1e-131) of identity to the homolog of *N*. *crassa* and 56% of identify (E Value = 0) to that of *A*. *nidulans* [17] (Table 1). The *P*. *oxalicum clrB* gene encodes a protein of 780 amino acid residues. Two introns of 76 and 64 nucleotides, which follow the characteristics of the *clrB* homolog in *N*. *crassa*, were identified [17]. The deduced transcription factor ClrB contains normal characteristics of Zn(II)_2_Cys_6_ binuclear cluster DNA binding motif near the N-terminus (residues 40–71) and the middle homology domain (residues 351–453) that is related to fungal specific transcription factors, including XlnR/XYR1 [10,11] and yeast regulatory protein GAL4 [36]. In this study, several putative cellulolytic transcription factors, such as PDE_03268, PDE_03964, and PDE_09881, also contain normal characteristics of these zinc binuclear cluster proteins (Table 1).

**Fig 2:**
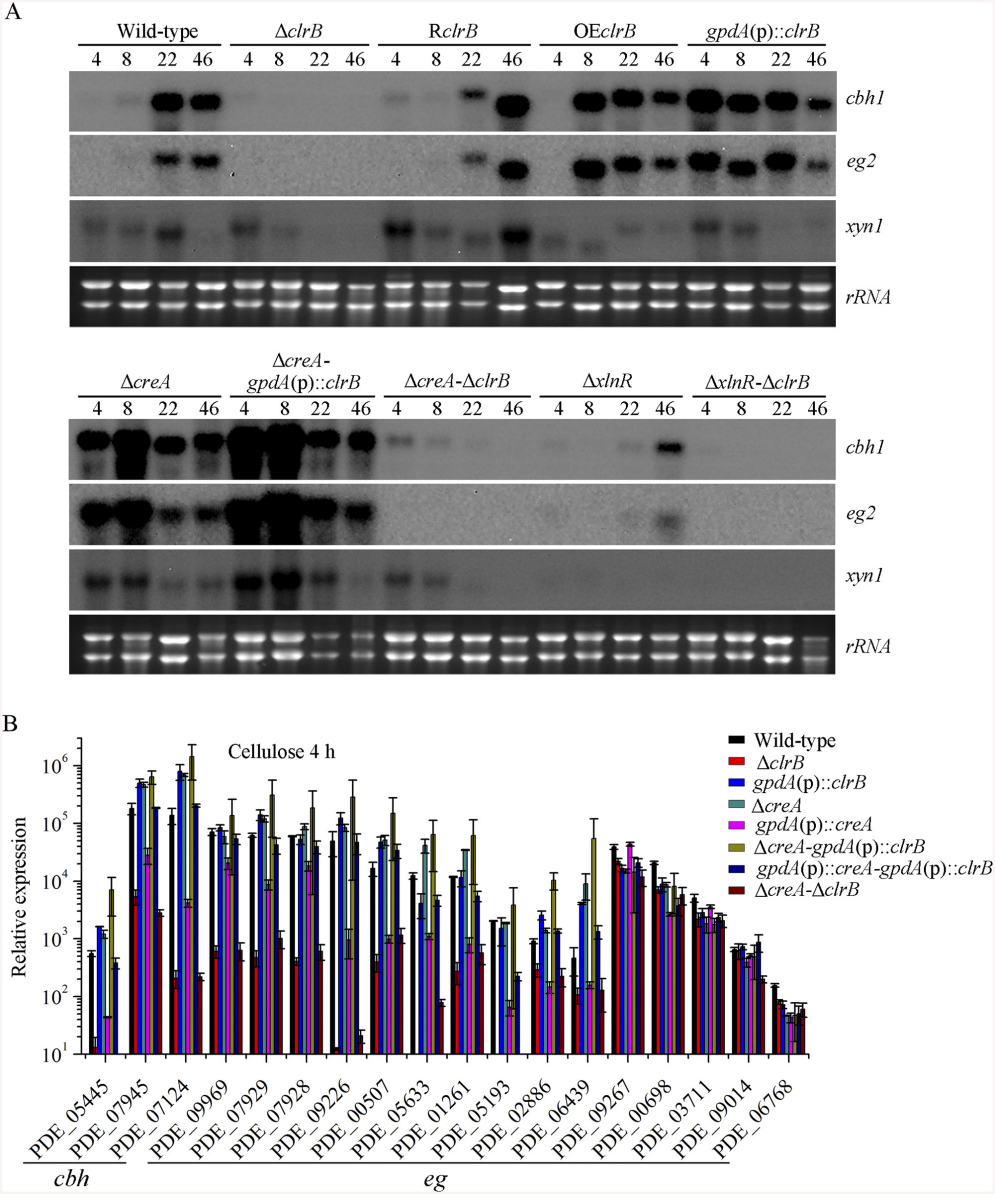
Regulation of cellulolytic gene expression by ClrB, XlnR and CreA. (A) Northern blot analysis of the transcription of cellulolytic genes in the mutants versus wild-type strain. The strains were pre-cultured on glucose for 22 hours and then were shifted to Vogel’s medium without carbon source for starvation cultivation for 2 hours, and then transferred to the same medium containing 2% (w/v) cellulose. 2 μg of total RNA was electrophoresed and blotted onto Hybond N+ nylon membrane. *cbh1*, *eg2* or *xyn1* mRNA was probed at different time points after shift to cellulose. (B) Under the same culture condition, gene expression levels of 3 cellobiohydrolase and 15 endoglucanase genes were determined in the mutants versus wild-type strain by q-PCR. All values were normalized using the actin gene value/10000. Values reported were means of three biological replicates of each strain. Error bars represent the standard deviation of 3 biological replicates.

To investigate the influence of ClrB on cellulase expression, we constructed a Δ*clrB* strain from *P*. *oxalicum* wild-type strain 114–2 (CGMCC 5302). The Δ*clrB* strain displayed significantly reduced growth on cellulose plate, but identical phenotype on glucose, xylan, or potato dextrose agar (PDA) plates relative to wild-type strain (Fig 1A). The Δ*clrB* mutant exhibited dramatically reduced cellulase activities when compared with the wild-type strain (Fig 1B–1D), similar to the recent findings with *clr-2*/*clrB* in *N*. *crassa*/*A*. *nidulans* [17]. Northern blot analysis was used to study the cellulolytic gene *cbh1* (PDE_07945), *eg2* (PDE_09226), and *xyn1* (PDE_08094) expressions in the wild-type and Δ*clrB* strains grown on cellulose. Fig 2A shows that the mRNA levels of *cbh1* and *eg2* in the Δ*clrB* mutant significantly decreased and could hardly be detected. As a result, a slight decrease in the *xyn1* transcription level was observed in the Δ*clrB* strain compared with that in the wild-type control, while a low transient increase expression of *xyn1* was observed at the fourth hour following the shift (Fig 2A). The introduction of a wild-type copy of *clrB* at the *clrB* locus (strain R*clrB*) completely restored the growth defects of the Δ*clrB* mutant in cellulose, as well as the cellulolytic enzyme activities of culture supernatants (Fig 1B–1D). These results demonstrated that ClrB might be in the central part of the transcriptional-regulatory network of cellulase expression by controlling the transcription of cellulolytic genes.

**Fig 3:**
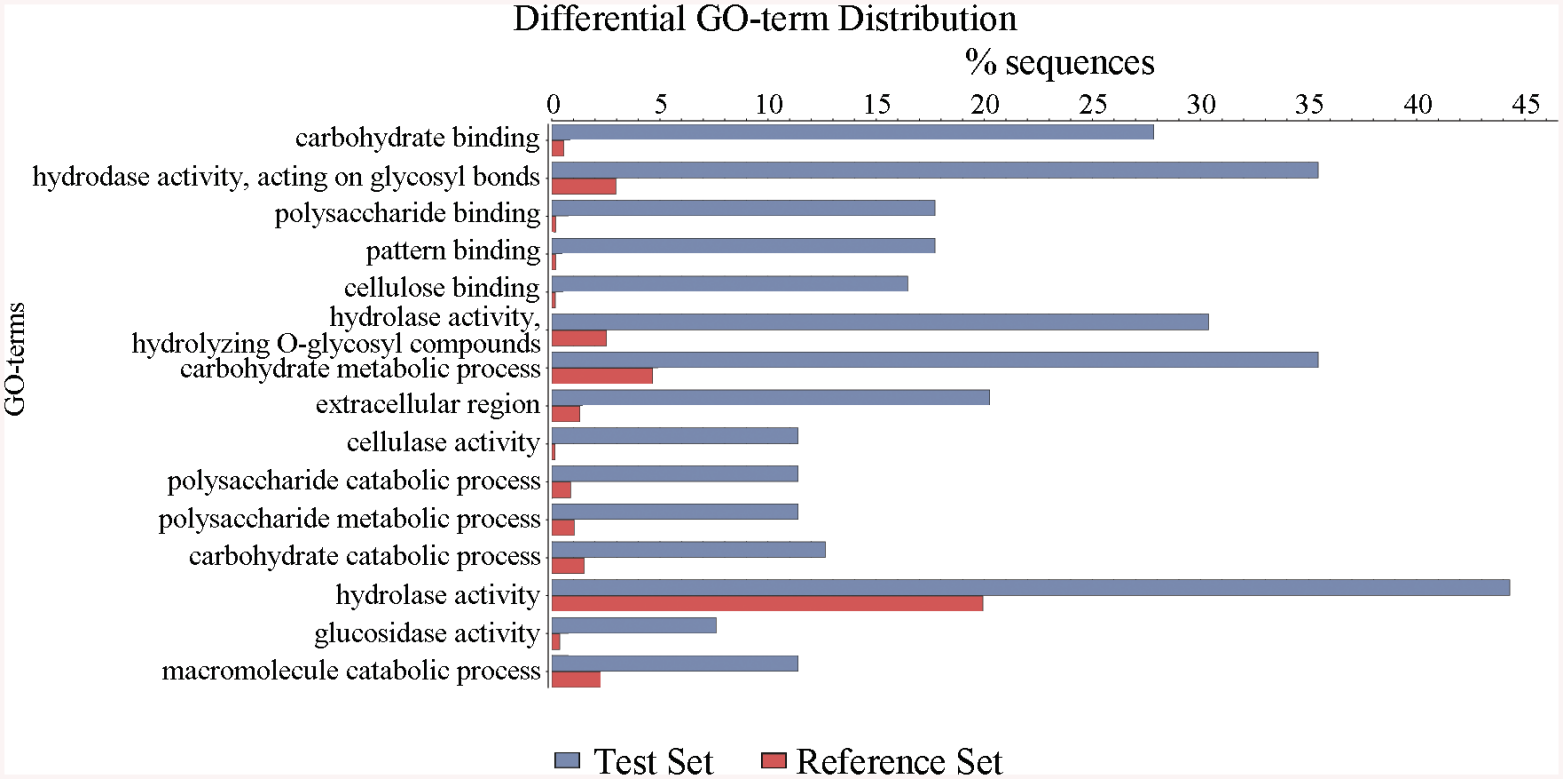
Defining the categories of genes potentially regulated by ClrB. The Δ*clrB* mutant and wild-type strains were pre-cultured on glucose for 22 hours and then shifted to Vogel’s media with no carbon source for 2 hours, and then transferred to Vogel’s medium containing 2% (w/v) cellulose for 4 hours. These differential expression genes were subjected to gene ontology enrichment analysis and percentages of genes varying significantly for ClrB regulon distributed within each functional category. The x-axis represents the percentage of genes in the corresponding GO class.

The *P*. *oxalicum* genome contains approximately 10,000 genes and is predicted to encode 18 cellulases, 51 hemicellulases, and other cellulolytic enzymes involved in plant biomass degradation [24]. Therefore, to build a comprehensive picture by which *P*. *oxalicum* responds to cellulose, we adopted RNA-Seq to measure genome-wide mRNA abundances in the *P*. *oxalicum* wild-type strain and Δ*clrB* mutant when exposed to Vogel’s minimal medium containing 2% cellulose for 4 hours. The three biological replicates of each strains showed a high Pearson correlation (S2 Fig). A total of 224 genes were differentially expressed between the Δ*clrB* and the wild-type strains on cellulose (S2 and S3 Tables). Of these genes, 103 genes showed lower transcription levels in the Δ*clrB* mutant than in the wild-type strain (S2 Table). These genes of decreased expression in *clrB* regulon were subjected to gene ontology enrichment analysis. Percentages of the genes distributed within each functional category are shown in Fig 3. Among these downregulated genes, 24 genes encoding transporters were enriched, including PDE_00607 (p = 7.99e-173), encoding cellodextrin transporter CdtC [29], and PDE_06576 (p = 2.96e-58), encoding putative maltose permease, which suggests that ClrB might also be involved in the cellodextrin and maltose metabolisms. In total, 32 genes encoding carbohydrate-active enzymes (CAZymes) were included, including 9 main cellulase genes and two of 11 β-glucosidases PDE_00579 (p = 1.98e-109) and PDE_04251 (p = 2.39e-42) (S2 Table), which demonstrates that the genes involved in plant cell wall deconstruction were significantly enriched. Only 6 of the 51 hemicellulase genes showed obvious reduction in transcription levels in the absence of ClrB, including PDE_02101 (p = 0.0033), PDE_06649 (p = 0.00046), PDE_01302 (p = 5.63e-12), PDE_09710 (p = 3.09e-09), PDE_05998 (p = 1.36e-37), and PDE_06023 (p = 1.87e-41) (S2 Table). These data demonstrated that ClrB might play a significant role in activating cellulase gene expression but has differential regulatory effects on cellulolytic and xylanolytic genes in the early inducing phase on cellulose (4 hours post-transfer).

In total, 121 genes showed higher transcription levels in the Δ*clrB* mutant than in the wild-type strain (S3 Table). Among these upregulated genes, only 7 genes encoding CAZy proteins, including two predicted hemicellulase genes PDE_07585 (p = 0.02) and PDE_08238 (p = 0.013), showed altered expressions. No cellulase, β-glucosidase, and xylanase genes were included. Genes in this set were enriched in oxidoreductase activity (p = 8.1e-6).

### Overexpression of *clrB* increased cellulase expression

To assess whether the overexpression of *clrB* enhanced the cellulase expression, two *clrB* overexpression recombinants were constructed under the control of its native promoter (strain OE*clrB*) and the *A*. *nidulans gpdA* promoter (strain *gpdA*(p)::*clrB*) in *P*. *oxalicum* wild-type strain [37], respectively. Both the OE*clrB* and *gpdA*(p)::*clrB* strains showed varied halos on 1% cellulose plates (Fig 1A) and showed almost 2.5- and 4.1-fold increases in filter paper enzyme activity (FPA), 2.5- and 4.0-fold increases in cellobiohydrolase (pNPCase) activity, and 8.7- and 16.5-fold increases in endoglucanase (CMCase) activity when grown on cellulose for 48 hours, respectively (Fig 1B–1E). Northern blot analyses also showed that the mRNA levels of *cbh1* and *eg2* in the *gpdA*(p)::*clrB* mutant were much higher than those in the wild-type strain on cellulose (Fig 2A).

To further test whether cellulase production was tightly responsive to *clrB* transcriptional abundance, we reconstructed the *PDE_02864*(p)::*clrB* expression cassette in which the *clrB* open reading frames and 3’ untranslated region were under the control of the novel promoter from the *PDE_02864* encoding 40S ribosomal protein S8. The *gpdA*(p)::*clrB*-*PDE_02864*(p)::*clrB* strain was constructed and showed even higher cellulase expression than those in the single *gpdA*(p)::*clrB* and *PDE_02864*(p)::*clrB* mutants on cellulose (S3 Fig). These results demonstrated that dose effect of *clrB* transcriptional abundance is important for the high expression for cellulases, and tunable cellulase expression may be controlled by the ClrB concentration under cellulose conditions.

### ClrB and XlnR additively regulated cellulolytic gene expression

In addition to ClrB, *P*. *oxalicum* XlnR is another cellulolytic activator in the Zn_2_Cys_6_ binuclear cluster motif superfamily that was identified previously along with the homologous *N*. *crassa* XLR-1 [12] and *T*. *reesei* XYR1 [10]. To demonstrate whether XlnR showed a differential role in cellulolytic gene expression regulation, the Δ*xlnR* and *gpdA*(p)::*xlnR* mutants with constitutive expression for *xlnR* under the control of the *A*. *nidulans gpdA* promoter in the wild-type strain were constructed. The Δ*xlnR* mutant showed a slightly decreased growth on both cellulose and xylan media, but not on glucose or PDA plates (Fig 1A). Northern blot results also showed that weak expressions for *cbh1* and *eg2* transcripts and invisible *xyn1* were observed in the Δ*xlnR* strain compared with that in the wild-type strain in the cellulose-containing medium (Fig 2A). These data indicated that *P*. *oxalicum* XlnR is a general transcription factor that regulates cellulase and xylanase expressions but not like *T*. *reesei* XYR1, which is the essential regulator that governed both cellulolytic and xylanolytic gene expressions [10].

**Fig 4:**
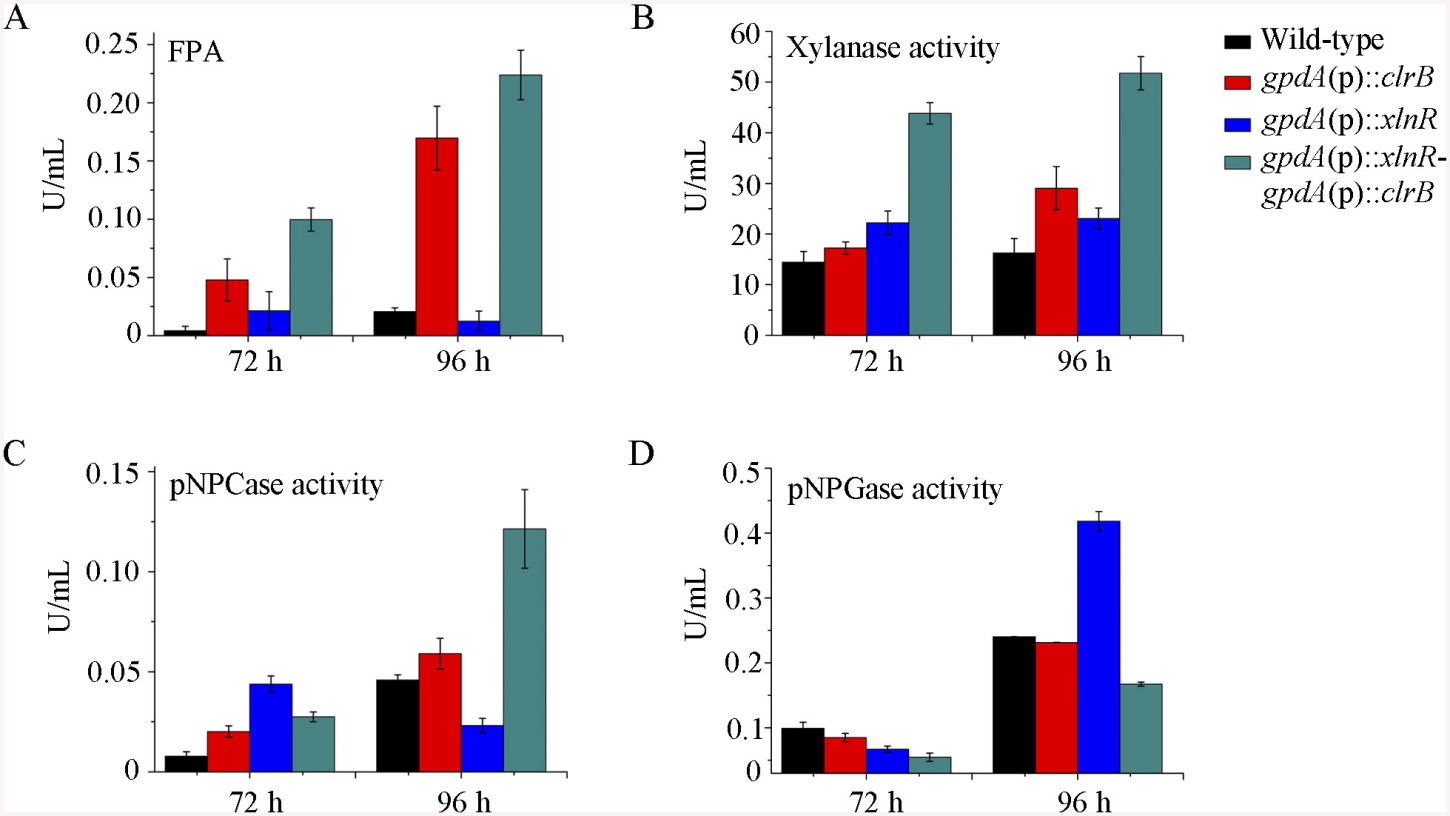
Enzyme activities of culture supernatants from mutants containing overexpression of *clrB* and/or *xlnR* on cellulose. (A-D) FPA, xylanase activity, pNPCase activity, and pNPGase activity in the culture supernatants from wild-type strain. The mutants grown on cellulose as a sole carbon source for 72 and 96 hours, respectively. Error bars represent the standard deviation of 3 biological replicates.

To further analyze whether synergistic or additive effects for these two major cellulolytic activators ClrB and XlnR existed, the *clrB* was also overexpressed in the *gpdA*(p)::*xlnR* mutant, and the *gpdA*(p)::*clrB*-*gpdA*(p)::*xlnR* mutant that contains simultaneously overexpressed ClrB and XlnR was obtained. The *gpdA*(p)::*clrB*-*gpdA*(p)::*xlnR* strain showed 1.3-, 1.7- and 2.1-fold increased expressions in FPA, xylanase, and pNPCase activities compared with that in the *gpdA*(p)::*clrB* strain after shift to cellulose for 96 hours (Fig 4A–4C), but decreased production in the pNPGase activity (Fig 4D). Conversely, lack of both ClrB and XlnR (Δ*clrB-*Δ*xlnR*) led to a greater abrogation of cellulase and xylanase expression than each absence mutation under cellulose growth conditions (Figs 1B and 2A). These data revealed that ClrB and XlnR had additive effects on positively regulating the cellulase and hemicellulase gene expressions, and the high-abundance transcripts of ClrB and XlnR could facilitate the induction of cellulase expression under cellulose growth conditions.

### CreA repressed the expression of cellulolytic genes and had a severe effect on morphology

PDE_03168 (CreA), as a homolog of the major carbon catabolite repressors in phylogenetically diverse fungi [7–9], played a negative role in the degradation of plant cell wall polymers. In this study, the Δ*creA* mutant of *P*. *oxalicum* consumed cellulose faster than the wild-type strain (Fig 1A), similar to the findings in *T*. *reesei cre1* [23] or *N*. *crassa cre-1* deletion strains [38]. The Δ*creA* strain grown on cellulose exhibited significantly increased cellulase activities compared with its parent strain, and showed almost 7.2-, 2.2-, 8.0-, and 4.4-fold increases in FPA, pNPCase, CMCase, and xylanase activities after shifting to cellulose for 96 hours, respectively (Fig 1B–1E). The Δ*creA* mutant exhibited higher steady state amounts of *cbh1* and *eg2* mRNA than that in the wild-type strain by Northern blot analysis (Fig 2A) and q-PCR experiments (Fig 2B) under cellulose growth conditions. However, the *gpdA*(p)::*creA* mutant that contains an overexpression of *creA* showed significantly lower cellulase gene expression than that in the wild-type strain when grown on cellulose (Fig 2B), which indicates that cellulolytic gene expression under CCR mediated by the CreA in *P*. *oxalicum* was responsive to the *creA* transcript abundances.

**Fig 5:**
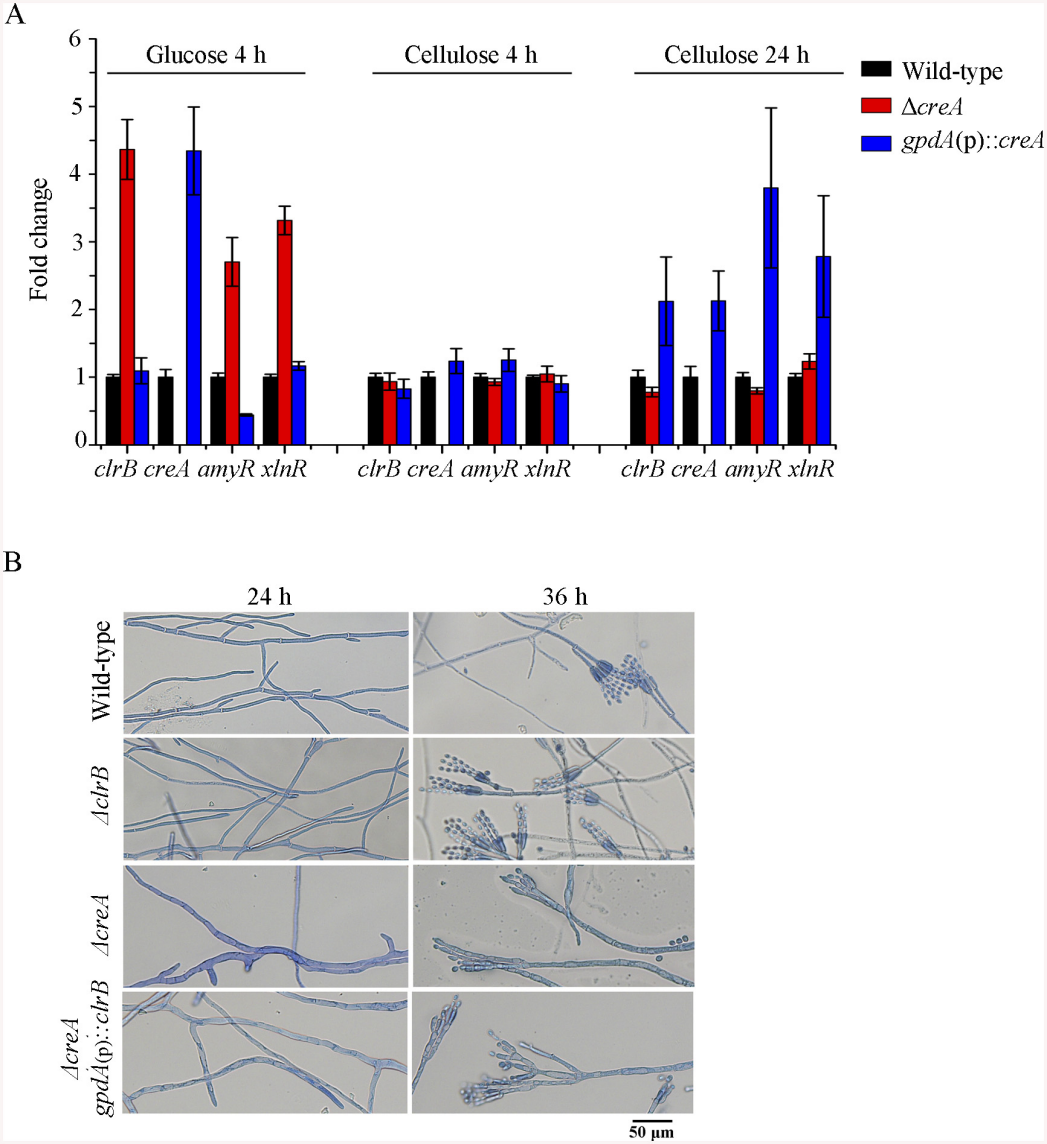
CreA regulated *clrB*, *clrB-2*, *xlnR*, and *amyR* expression and caused defects in microscopic phenotypes. (A) Transcription expression determination for the mutants versus wild-type after a shift to 2% glucose for 4 hours or 2% cellulose for 4 and 24 hours by q-PCR. (B) Microscopic phenotypes of wild-type, Δ*clrB*, Δ*creA*, and Δ*creA-gpdA*(p)::*clrB* strains. The pictures were taken corresponding to hyphae growing onto the cover glass on glucose. Hyphae were stained by lactophenol cotton blue. Scale bar = 50 μm in panel.

Although the Δ*creA* mutant produced higher cellulase expression than its parental strain *P*. *oxalicum* 114–2, we could not entirely exclude the possibilities that the increase of cellulolytic enzyme in Δ*creA* mutant might be the partial cause of the enhancement of cellulolytic activators for ClrB and XlnR. To test these hypotheses, q-PCR was performed and the expression levels for *clrB* and *xlnR* in the Δ*creA* mutant showed near to that in the wild-type strain on cellulose but obvious increase under glucose-repressing conditions (Fig 5A). These data indicated that CreA repressing the expression of *clrB* and *xlnR* was associated with the carbon source used in the medium, and CreA and ClrB, as well as CreA and XlnR might form a transcriptional cascade that regulates the cellulolytic gene expression in *P*. *oxalicum*.

Previously, we reported that a cellulase hyper-producer *P*. *oxalicum* mutant JU-A10, which bears a shift mutation at *creA* locus, has an extremely severe effect on morphology, including short and thicker hyphae [1]. The *creA* deletion in *P*. *oxalicum* wild-type strain 114–2 showed smaller colonies and reduced hyphae growth (Fig 1A). Under the microscope, the Δ*creA* mutant on glucose plates exhibited considerably robust hyphae (Fig 5B). Thus, some hyphae morphology mutation in *P*. *oxalicum* mutant JU-A10 [1] might be specific results caused by the shift mutation at the *creA* location in the JU-A10 mutant by chemical mutagenesis. Similarly, the *T*. *reesei* Δ*cre1* mutant also displayed shorter and more robust hyphae than its parental strain [23]. We hypothesized that the morphology mutation caused by *creA* homologs may be general in filamentous fungi. However, whether hyphae morphology mutation was related to the increase in protein production and cellulase expression in these mutants containing deletion of *creA* needs to be further investigated in the future.

### Simultaneous overexpression of ClrB and lack of CreA caused strong synergistic effects on cellulase expression

Cellulase genes were responsive to both major opposing regulators ClrB and CreA. First, whether the low cellulase expression exhibited by the Δ*clrB* mutant could be recovered when knocking out the CreA encoding gene remains unknown. To bypass this problem, we deleted the *creA* in the Δ*clrB* strain and obtained the Δ*creA*-Δ*clrB* mutant. Northern blot, presented in Fig 2A, shows that Δ*creA*-Δ*clrB* mutant exhibited a slightly higher amount of *cbh1* mRNA than that in the Δ*clrB* strain. The Δ*creA*-Δ*clrB* mutant also showed 3.1-, 0.5-, 3.1-, and 1.8-fold increases in FPA, pNPCase, CMCase, and xylanase activities relative to wild-type strain after shifting to cellulose for 96 hours, respectively (Fig 1B). In contrast to the Δ*clrB* mutant, the Δ*creA*-Δ*clrB* strain produced visible halo in the cellulose medium plate when cultured for 9 days (S4 Fig). These data indicated that the lack of CreA partially rescued the cellulase expression defect in the *ΔclrB* mutant. Conversely, the *gpdA*(p)::*creA*-*gpdA*(p)::*clrB* strain that contains simultaneous overexpressions of CreA and ClrB also showed increases in the transcription expression levels for most of the cellulase genes compared with that in the *gpdA*(p)::*creA* mutant (Fig 2B).

The strong cellulase production observed in the Δ*creA* and *gpdA*(p)::*clrB* mutants indicated that these two genes encoded the major transcription factors that oppositely regulate cellulolytic gene expression. Although, the triple-mutant RE-10 (Δ*bgl2*-Δ*creA*-*gpdA*(p)::*clrB*) was recently obtained [39], it is still not known why high cellulase expression in which occurs. Therefore, we deleted *creA* in the *gpdA*(p)::*clrB* strain to determine whether their functions in cellulase production were synergistic. To our knowledge, no reports have been described to perform this to determine their synergistic effects on cellulase induction expression in other cellulolytic fungi. Interestingly, the strain with the combination of *creA* deletion and overexpression of *clrB* displayed a much larger halo around its colony than that of the Δ*creA* and *gpdA*(p)::*clrB* mutants on cellulose plate (Fig 1A). The differences in the FPA, pNPCase, CMCase, and xylanase activities were even more pronounced (11.6-, 11.6-, 58.6-, and 15.9-fold higher, respectively) between the Δ*creA*-*gpdA*(p)::*clrB* and wild-type strains (Fig 1B). The Δ*creA-gpdA*(p)::*clrB* mutant exhibited higher steady-state amounts of *cbh1*, *eg2*, and *xyn1* mRNA than that of the Δ*creA* and *gpdA*(p)::*clrB* strains under cellulose growth conditions by Northern blot analysis (Fig 2A). Q-PCR results also indicated that all three cellobiohydrolase and 10 endoglucanase genes showed strong synergistic increases in transcription levels with the exception of five endoglucanase genes for PDE_009267, PDE_00698, PDE_03711, PDE_09014, and PDE_06768 when grown on cellulose (Fig 2B). In addition, three of the 11 β-glucosidase genes (PDE_00579, PDE_04251, and PDE_04859) showed similar induction patterns as the above thirteen cellulase genes in the Δ*creA-gpdA*(p)::*clrB* mutant on cellulose (S5 Fig). These data showed that the simultaneous overexpression of ClrB and lack of CreA have synergistic effects on cellulolytic and xylanolytic gene expressions.

### Transcriptome analysis of the combinatorial control by ClrB and CreA

To build a comprehensive picture by which *P*. *oxalicum* responds to cellulose, the genome-wide mRNA abundance in *P*. *oxalicum* wild-type strain was first measured on Vogel’s medium with no carbon or 2% glucose for 4 hours as an alternative reference. A total of 581 genes were differentially expressed between Avicel and no carbon cultures at the fourth hour (|log2(fold change)| > 1 and probability ≥ 0.8), 155 and 117 of which showed greater and lower expression levels on cellulose than that under either glucose growth or no carbon conditions, respectively (S4 and S5 Tables). We refer to this gene set, including 272 genes, as a “cellulose regulon” for *P*. *oxalicum*. The cellulose regulon encompassed 10 of 18 predicted cellulase genes, 30 of 51 predicted hemicellulase genes, and 3 of 11 predicted β-glucosidase genes PDE_02736 (p = 1.65e-07), PDE_00579 (p = 9.24e-174), and PDE_04251 (p = 1.02e-80) (S4 and S5 Tables). To further elucidate the regulation mechanisms for the opposing regulators CreA and ClrB in the gene expression of *P*. *oxalicum* under cellulose growth conditions, three biological replicates of Δ*creA*, Δ*creA*-Δ*clrB*, and Δ*creA-gpdA*(p)::*clrB* mutants were also subjected to transcriptional profiling at the same condition. The three biological replicates of each mutant showed a high Pearson correlation (S6 Fig) and demonstrated the reliability of RNA-Seq. The gene ontology enrichment analyses for these 117 decreased genes of cellulose regulon showed no statistically significant results with the threshold (FDR < 0.05).

**Fig 6:**
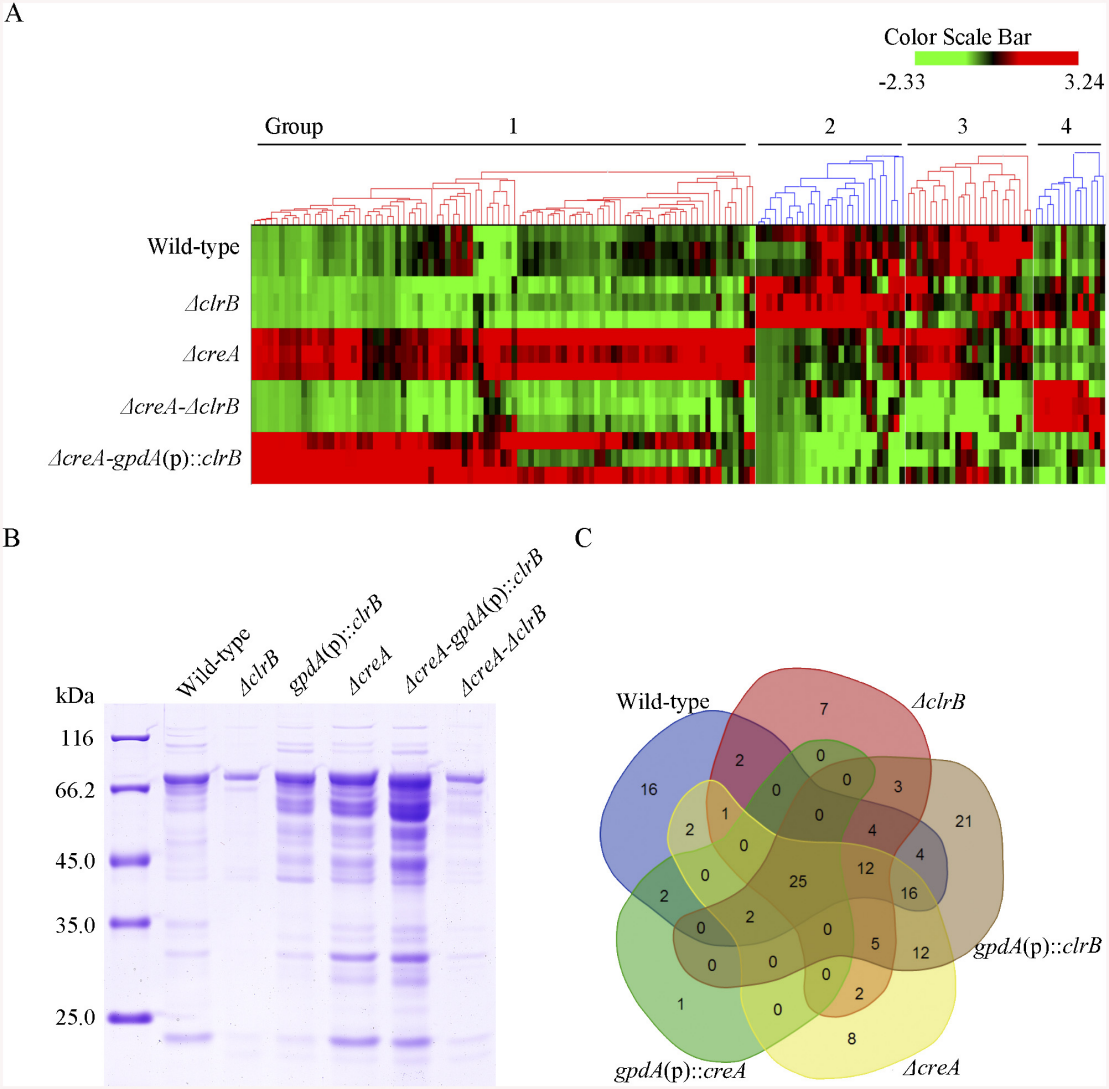
Heatmap of cellulose regulons and protein profiles of culture supernatants from *P*. *oxalicum* strains containing mutations of *clrB* or/and *creA* genes. (A) The hierarchical clustering of expression patterns for the 155 increased genes of cellulose regulon in the mutants versus wild-type strain. Hierarchical clustering analysis was performed using software HCE3.5. Heat maps showing log (RPKM) from minimum (bright green) to maximum (red) revealed four gene sets. (B) SDS-PAGE of proteins from unconcentrated culture supernatants of wild-type strain and *clrB* or/and *creA* mutants when cultured on glucose for 22 hours then shifted to cellulose for 96 hours. (C) Venn diagram from analysis of secretomes of culture supernatants from *P*. *oxalicum* mutants. The loading samples were normalized to same concentration with buffer.

The hierarchical clustering of expression patterns for the 155 increased genes of cellulose regulon in the wild-type strain and the Δ*clrB*, Δ*creA*, Δ*creA*-Δ*clrB*, and Δ*creA-gpdA*(p)::*clrB* mutants displayed four classes of genes with similar expression patterns. A heat map that depicts the relative expression levels of selected genes from groups 1 to 4 is shown in Fig 6A.

Group 1 consisted of 91 genes (S4 Table). Most of the genes in this set exhibited strong synergistic induction effects on transcriptional expression in the Δ*creA*-*gpdA*(p)::*clrB* mutant (Fig 6A). In this cluster, we detected a significant enrichment of genes involved in hydrolase activities (p = 1.5e-30). This group included 9 of 18 cellulase genes (PDE_00507, PDE_09969, PDE_07929, PDE_07928, PDE_05633, PDE_09226, PDE_07124, PDE_07945, and PDE_01261), two β-glucosidase genes (PDE_00579 and PDE_04251), and three sugar transporter genes for cellodextrin transport-1 (PDE_00607), major facilitator superfamily (MFS) maltose transporter (PDE_06576) and monosaccharide transporter (PDE_04857), all of which showed significantly low levels in the Δ*creA*-Δ*clrB* strain versus Δ*creA* mutant, but similar transcription activities between the Δ*creA*-Δ*clrB* and Δ*clrB* mutants, which indicates that the expression levels for these enriched cellulolytic genes were repressed by CreA but ClrB dependent under inducing conditions. Cellodextrin transport-2 genes (PDE_007257 and PDE_00753) were also CreA repressed and ClrB induced but not ClrB dependent. A total of 16 out of the 51 hemicellulase genes (PDE_06649, PDE_02101, PDE_00016, PDE_04478, PDE_01302, PDE_09278, PDE_06023, PDE_04182, PDE_08094, PDE_02514, PDE_00752, PDE_09710, PDE_06067, PDE_05998, PDE_02418, and PDE_07897) were enriched in this subset, and most of which were significantly repressed by CreA, but only four genes (PDE_05998, PDE_06023, PDE_01302, and PDE_09710) were ClrB induced. Noticeably, the expression levels for PDE_05998 (beta-mannosidase) and PDE_06023 (beta-1, 4-mannanase) were ClrB dependent. Interestingly, three genes involved in starch degradation, encoding starch binding domain-containing protein (PDE_01354), glucoamylase (PDE_05527), and α-amylase (PDE_01201) were also downregulated in the Δ*clrB* mutant and upregulated in the Δ*creA* mutant, which suggests that the expression for these amylase genes were tightly co-regulated with the cellulase genes under cellulose growth conditions. A significant enrichment of genes (PDE_09832, PDE_04496, and PDE_09715) involved in the sporulation process (p = 8.2e-5) was also detected. In addition, 21 genes that encode hypothetical proteins were within this dataset (23%). The above data suggested that this cluster might represent the most central components associated with cellulose deconstruction, which indicates that this large synergistic activation by engineering CreA and ClrB might be specific to these cellulolytic target genes.

Group 2 consisted of 27 genes (S4 Table). The expression levels of this set were partially induced in the Δ*clrB* mutant and repressed in the Δ*creA* mutant, but were significantly downregulated in the Δ*creA-gpdA*(p)::*clrB* strain compared with that in the wild-type strain (Fig 6A). Although GO-term analyses revealed that no statistically significant results with the threshold (FDR < 0.05), genes that encode β-glucosidase (Bgl1, PDE_02736), endo-β-1, 4-glucanase (PDE_09267), and two β-xylosidases (PDE_07334 and PDE_08037) were included in this dataset.

Group 3 consisted of 23 genes (S4 Table), and the expression levels of which were partially repressed in the Δ*creA* and Δ*clrB* mutants, and were cumulatively repressed in the Δ*creA-*Δ*clrB* mutant, but were partially recovered in the Δ*creA*-*gpdA*(p)::*clrB* mutant (Fig 6A). The GO-term analyses of the dataset of 23 genes showed a significant enrichment in the carbohydrate metabolic process (p = 3.6e-7). Five hemicellulase genes that encode PDE_06306, PDE_03572, PDE_07080, PDE_03573, and PDE_08036 and one α-glucosidase (PDE_00400) were also within this group.

Group 4 consisted of 13 genes (S4 Table). The group genes were slightly induced in the Δ*clrB* mutant and showed no induction in the Δ*creA* mutant, but were significantly induced in the Δ*creA-*Δ*clrB* mutant compared with that in the wild-type strain (Fig 6A). One gene that encodes β-xylosidase (PDE_00049) was within this dataset, and eight genes encoded hypothetical proteins.

### Secretome analysis of the combinatorial control by ClrB and CreA on cellulose

Secreted proteins are expected to play a crucial role in cellulose degradation because of the nature of cellulolytic system for the deconstruction of plant cell walls. However, whether the cellulolytic protein concentration in secretome potentially correlates with their relative mRNA levels, and how this induction stimulus under cellulose conditions causes *P*. *oxalicum* to manipulate its secretome to facilitate cellulose degradation remain unclear. Thus, extensive secretome surveys using label-free LC-MS/MS were conducted to analyze the secretomes under cellulose conditions systematically. Herein, a supernatant from 4-day old wild-type culture grown on cellulose was digested with trypsin and analyzed by LC-MS-MS. For this purpose, 157 nonredundant proteins (P-value < 0.01) were identified based on a single or several peptide entries from the *P*. *oxalicum* protein database, the pI of which was concentrated in a pH range of 4–7, and 86 proteins were predicted to be secreted based on SignalP computational analysis (SignalP 4.1 Server, http://www.cbs.dtu.dk/services/SignalP/) (S6 Table). Proteins with predicted activities on carbohydrates in the *P*. *oxalicum* wild-type secretome dataset existed, including 10 of 18 predicted cellulases, one β-glucosidase Bgl1, and 10 of 51 predicted hemicellulases (S6 Table).

Subsequently, a question was posted with regard to the extent to which ClrB can be attributed to the regulation of extracellular protein abundances in the cellulolytic system. The total protein in the Δ*clrB* mutant culture supernatant was only 30% of that in the wild-type strain (Fig 6B). To characterize the secretome changes in response to the crucial regulator ClrB further, a total of 104 predicted secreted proteins in the *gpdA*(p)::*clrB* strain (S6 Table) and 61 predicted secreted proteins in the Δ*clrB* mutant (S6 Table) were identified after the shift to 2% cellulose for 4 days, respectively. These observations demonstrate that ClrB significantly increased the number of extracellular proteins on cellulose. The Δ*clrB* secretome dataset under cellulose growth condtions included only 5 of 18 predicted cellulases and 5 of 51 predicted hemicellulases in *P*. *oxalicum* genome. The *gpdA*(p)::*clrB* secretome dataset included 13 of 18 predicted cellulases and 10 of 51 predicted hemicellulases. A comparison between the secretomes of Δ*clrB* and those of *gpdA*(p)::*clrB* grown on cellulose showed that only 49 proteins overlapped (S6 Table), including 5 of 18 cellulases and 4 of 51 hemicellulases. These data indicate that ClrB enhanced various cellulolytic enzymes, including their secretion strength. More importantly, the observed changes of cellulolytic proteins in Δ*clrB* and *gpdA*(p)::*clrB* strains highly correlated with their corresponding mRNA abundances and broadly mirrored the ClrB-specific positive roles for the transcript expression of cellulolytic genes (S6 Table).

Concurrently, to evaluate the CreA-influenced extracellular proteins, we first used sodium dodecyl sulfate polyacrylamide gel electrophoresis (SDS-PAGE) to analyze the secretomes of Δ*creA* and *gpdA*(p)::*creA* cultures under cellulose growth conditions (Fig 6B). The protein pattern of Δ*creA* mutant showed more bands than that of *gpdA*(p)::*creA* mutant on SDS-PAGE (Fig 6B). The total protein concentration in Δ*creA* mutant culture supernatant was 2.6-fold higher than that in the wild-type strain (Fig 6B). We further investigated the extracellular proteins influenced by CreA by adopting the label-free LC-MS/MS analysis to identify and quantify the proteins. A total of 85 and 30 predicted secretion proteins in Δ*creA* and *gpdA*(p)::*creA* mutants were identified when grown on cellulose (S6 Table), respectively. The SDS-PAGE and LC-MS/MS analysis results for culture secretomes revealed that protein secretion, including protein abundance and distribution, was dramatically repressed by CreA under cellulose growth conditions.

The secretome of Δ*creA*-*gpdA*(p)::*clrB* mutant was investigated under cellulose conditions to further identify the regulon with ClrB and CreA synergistic effects at the secretome levels. The total amount of the secreted protein in Δ*creA-gpdA*(p)::*clrB* culture supernatant displayed a 7.5-fold increase compared with that in the wild-type strain (Fig 6B). Label-free LC-MS/MS analysis was used, and 97 predicted secretion proteins were identified in Δ*creA-gpdA*(p)::*clrB* mutant (S6 Table). These proteins included 10 of 18 cellulases, and 15 of 51 hemicellulases. No β-glucosidase was detected in Δ*creA-gpdA*(p)::*clrB* mutant.

To assess differences more accurately in protein distribution, the secretomes from Δ*clrB*, *gpdA*(p)::*clrB*, Δ*creA*, *gpdA*(p)::*creA*, and Δ*creA-gpdA*(p)::*clrB* mutants were combined to locate targets that were the basal components in *P*. *oxalicum* secretomes under cellulose growth conditions (Fig 6C and S6 Table). In these datasets, we identified that 25 proteins overlapped (S6 Table), including two cellobiohydrolases (i.e., PDE_07945 and PDE_07124), three endoglucanases (i.e., PDE_09969, PDE_07929, and PDE_09226), four hemicellulases (i.e., PDE_02101, PDE_06023, PDE_04182, and PDE_08094), two extracellular membrane proteins (i.e., PDE_08075 and PDE_02536) that contain common in fungal extracellular membranes domain, three amylases (i.e., PDE_01201 (alpha-amylase Amy13A), PDE_01354 (protein with starch binding domain), and PDE_09417 (glucoamylase GluA/Amy15A)), and five hypothetical proteins (i.e., PDE_03934, PDE_06089, PDE_07106, PDE_09289, and PDE_00667). All these proteins existed in the wild-type strain secretome under the same culture condition (S6 Table). After a 4-h shift from no carbon source, the transcription expression levels of 14 of these proteins increased in cellulose versus those in glucose (S6 Table).

### Bgl2 expression level was ClrB-dependent and deletion of *bgl2* facilitates the synergistic induction expression of cellulase genes in *P*. *oxalicum*

**Fig 7:**
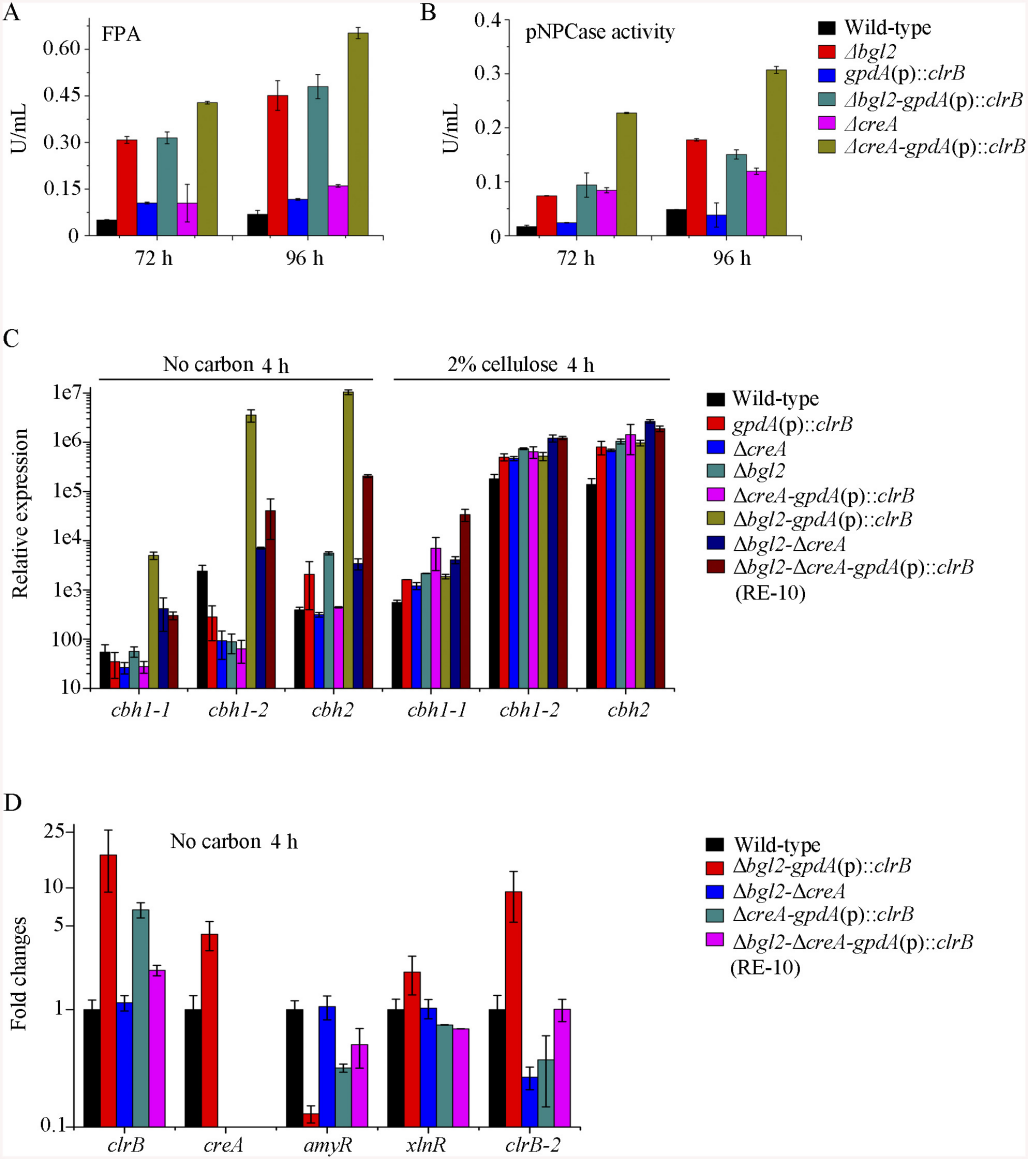
Lack of Bgl2 and overexpression of ClrB or lack of CreA additively induced cellulase expression. (A, B) Lack of Bgl2 in *gpdA*(p)::*clrB* or Δ*creA* mutant additively enhanced FPA and pNPCase activity under cellulose growth conditions. (C) q-PCR measurements of three *cbh* gene expression in mutants versus wild-type and each single mutation strains shifted to media lacking carbon source versus on cellulose for 4 hours. Gene expression levels were normalized to actin gene as a control. (D) The transcription levels for *clrB*, *clrB-2*, *creA*, *xlnR* and *amyR* in the mutants versus wild-type strain under no carbon conditions by q-PCR. Expression levels were normalized to the wild-type.

The above data imply that β-glucosidase gene *bgl2* (PDE_00579) was the major object regulated by ClrB and CreA at the level of transcription (S5 Fig). However, lack of CreA in the Δ*clrB* mutant could not recapitulate *bgl2* expression level (S5 Fig), suggesting that *bgl2* expression level was strictly ClrB-dependent under induction conditions. Considering the upregulation of cellulolytic genes in Δ*bgl2* mutant [27], we speculated that the expression levels for cellulolytic genes were further enhanced via the overexpression of *clrB* or deletion of *creA* in a *bgl2* deletion background. In support of this hypothesis, we first constructed Δ*bgl2*-*gpdA*(p)::*clrB* and Δ*bgl2*-Δ*creA* mutants. The cellulase gene transcription levels and cellulase activities, which were greater than those in each single mutation strain on cellulose (Fig 7A and 7B, and S7A and S7B Fig), as well as the Δ*bgl2*-Δ*creA* strains exhibited even more cellulase productions than Δ*bgl2*-*gpdA*(p)::*clrB* mutant on cellulose (Fig 7A and 7B, and S7A and S7B Fig). These results suggest that the decrease of intracellular β-glucosidase activity may facilitate the transcriptional induction of cellulolytic genes.

Cellulase gene expression depends on the presence of the inducers and on the positive regulation of activators in cellulolytic fungi [2]. Recent studies indicated that the constitutive expressions of *N*. *crassa clr-2* [20] and *T*. *reesei xyr1* [40] could recapitulate the response to cellulose when incubated without carbon. To assess whether the constitutive expression of *clrB*, deletions of *bgl2* or *creA*, or combination of these genetic manipulations was sufficient for the induction of cellulase genes independent of inducers, the cellulase expression levels in Δ*bgl2*, *gpdA*(p)::*clrB*, Δ*creA*, Δ*bgl2*-*gpdA*(p)::*clrB*, Δ*bgl2*-Δ*creA*, and Δ*bgl2*-*gpdA*(p)::*clrB*-Δ*creA* mutants were evaluated as opposed to the wild-type strain when cultures were shifted from a glucose medium to a carbon-free medium for 4 hours. The findings revealed that Δ*bgl2*-*gpdA*(p)::*clrB* mutant exhibited even more transcriptional abundances on carbon-free medium than on cellulose (Fig 7C and S7C Fig), whereas the “starvation response” for cellulase expression also occurred in *Δbgl2*-*ΔcreA* and RE-10 mutants (Fig 7C and S7C Fig). However, such a response was significantly low compared with that in Δ*bgl2*-*gpdA*(p)::*clrB* mutant (Fig 7C and S7C Fig). Consistent with these results, the pNPCase and CMCase activities were more than 10-fold higher in Δ*bgl2*-*gpdA*(p)::*clrB* strain than those in each single mutant and wild-type strain under carbon-free conditions (S8A and S8B Fig). The *creA*, *amyR* and *xlnR* transcription abundances in Δ*bgl2*-*gpdA*(p)::*clrB*, Δ*bgl2*-Δ*creA*, Δ*bgl2*-*gpdA*(p)::*clrB*-Δ*creA* and wild-type strains were assayed to test whether CreA, AmyR and XlnR mediated the synergistic induction in Δ*bgl2*-*gpdA*(p)::*clrB* strain when subjected to starvation. The findings indicated that *amyR* had a 7.7-fold decrease, whereas *creA* had a 4.3-fold increase and *clrB* had a 17.5-fold increase in Δ*bgl2*-*gpdA*(p)::*clrB* mutant versus that in the wild-type strain (Fig 7D). These data signify that AmyR may share a key role in the “starvation response” for cellulolytic genes and provide a novel insight into the cellulase gene regulatory mechanisms during energy abstinence.

### Improvement of *P*. *oxalicum* cellulase production via reconstruction of expression regulation network (RERN)

**Fig 8:**
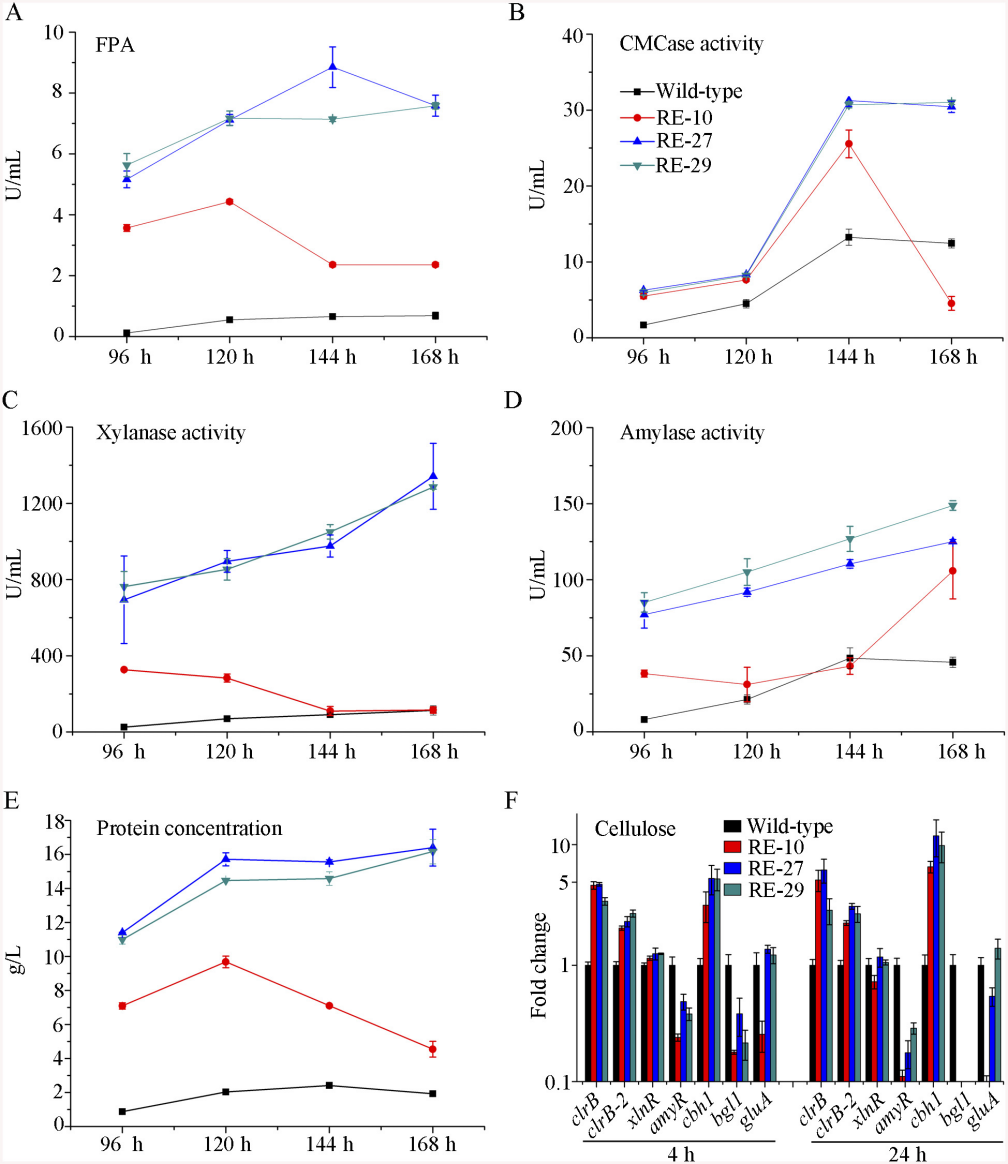
Overexpression of *clrB* or *xlnR* in triple-mutant RE-10 enhanced hydrolase expression under inducer-inducing conditions. (A-E) The FPA, pNPCase activity, xylanase activity, amylase activity and total secreted protein concentration of culture supernatants from RE-27 (Δ*bgl2*-Δ*creA*-*gpdA*(p)::*clrB*-*PDE_02864*(p)::*clrB*) and RE-29 (Δ*bgl2*-Δ*creA*-*gpdA*(p)::*clrB*-*PDE_02864*(p)::*xlnR*) mutants versus the wild-type and triple-mutant RE-10 (Δ*bgl2*-Δ*creA*-*gpdA*(p)::*clrB*) were separately evaluated when grown on wheat bran medium in flasks. (F) q-PCR measurements of *clrB*, *clrB-2*, *xlnR*, *amyR*, *cbh1*, *bgl1* and *gluA* expression in RE-27 and RE-29 mutants versus the wild-type and RE-10 strains on cellulose. Expression levels were normalized to the wild-type.

Given the dose-controlled or additive regulation of cellulase genes by ClrB and XlnR presented in *gpdA*(p)::*clrB*-*PDE_02864*(p)::*clrB*, *gpdA*(p)::*xlnR*, and *gpdA*(p)::*clrB*-*gpdA*(p)::*xlnR* mutants (Fig 4A–4D and S3 Fig), and the synergistic transcriptional induction of cellulolytic genes in Bgl2-deficient background (Fig 7A and 7B, and S7A and S7B Fig), we assessed whether the dose effects of ClrB and XlnR transcriptional abundance were feasible in further enhancing the cellulase expression in triple-mutant RE-10 [39]. We examined this hypothesis by reconstructing two overexpression cassettes (i.e., *PDE_02864*(p)::*clrB-sur* and *PDE_02864*(p)::*xlnR-sur*), in which the *sur* cassette (conferring resistance to sulfonylurea) was used as a resistance marker. These overexpression cassettes for *clrB* and *xlnR* were separately transformed into RE-10 [39]. The quadruple mutants RE-27 (Δ*bgl2*-Δ*creA*-*gpdA*(p)::*clrB*-*PDE_02864*(p)::*clrB*) and RE-29 (Δ*bgl2*-Δ*creA*-*gpdA*(p)::*clrB*-*PDE_02864*(p)::*xlnR*) were obtained, and their cellulase expression abilities were separately evaluated on cellulose and wheat bran media. Although all these experiments were performed in flasks, both RE-27 and RE-29 mutants showed more cellulolytic and xylanolytic enzyme activities and secretion abilities than RE-10 (Fig 8A–8C and 8E, and S9A–S9F Fig). When grown on a medium with 2% of cellulose as a sole carbon source for 120 h, RE-27 mutant displayed 62.3%, 34.8%, 288.5% and 28.0% greater FPA, pNPCase activity, xylanase activity and total secreted protein level, but 26.3% lower pNPGase activity, respectively, than RE-10 (S9A–S9E Fig). Similarly, the RE-29 mutant showed 55.3%, 44.1%, 255.2% and 20.6% greater FPA, pNPCase activity, xylanase activity and total secreted protein level, but 39.8% lower pNPGase activity, respectively (S9A–S9E Fig). We also observed a significant decrease in *amyR* expression level for both RE-27 and RE-29 mutants as compared to the wild-type strain on cellulose by q-PCR (Fig 8F). The findings further signify that AmyR may share a key role in the regulatory network for cellulolytic genes. When grown on a wheat bran medium, RE-27 mutant exhibited FPA (8.85±0.66 U/mL), CMCase activity (31.25±0.77 U/mL), xylanase activity (1341.97±172.94 U/mL), amylase activity (125.07±1.32 U/mL) and total secreted protein concentration (16.40±1.08 g/L), and RE-29 mutant also displayed FPA activity (7.58±0.34 U/mL), CMCase activity (31.03±0.29 U/mL), xylanase activity (1285.93±11.12 U/mL), amylase activity (148.82±3.19 U/mL) and total secreted protein concentration (16.16±0.72 g/L), respectively (Fig 8A–8E). These data signify that the dose-controlled regulation mechanisms of the cellulolytic regulators are a promising strategy for cellulolytic fungi to develop enzyme hyper-producers via the RERN technology.

### AmyR repressed cellulolytic gene expression and *amyR* expression was regulated by ClrB

**Fig 9:**
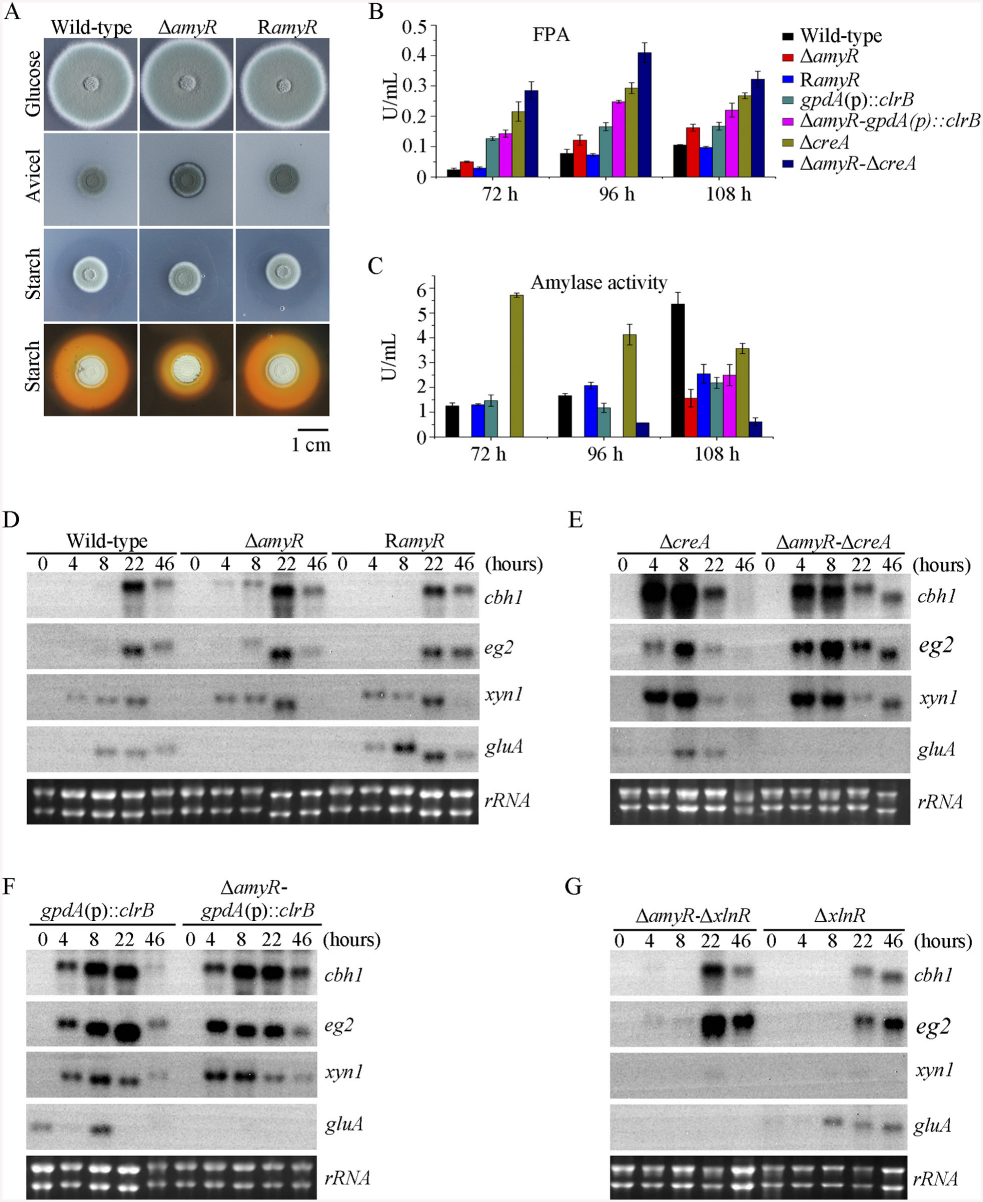
Lack of AmyR decreased amylase activity and increased cellulase expression on cellulose. (A) Deletion of *amyR* resulted in visible halo on 1% cellulose plate and decreased starch-degrading halo on starch plate, respectively. (B, C) Lack of AmyR and overexpression of ClrB or lack of CreA additively induced cellulase expression and decreased amylase expression on cellulose. (D-G) Northern blot analysis of the transcription of *cbh1*, *eg2*, *xyn1* and *gluA* genes in Δ*amyR*-Δ*creA*, Δ*amyR*-*gpdA*(p)::*clrB* and Δ*amyR*-Δ*xlnR* mutants versus each single mutation strains on cellulose. The mutants were pre-cultured on glucose for 22 hours and then shifted to Vogel’s media with no carbon source for 2 hours, and then transferred to Vogel’s medium containing 2% (w/v) cellulose for 4, 8, 22 or 46 hours. 2 μg of total RNA was electrophoresed. *cbh1*, *eg2*, *xyn1* or *gluA* mRNA was probed at different time points after shift to cellulose.

In the above *P*. *oxalicum* cellulose regulon and basal secretome components, some enzymes involved in starch degradation were tightly associated with the cellulolytic protein expression on cellulose. The “starvation response” in Δ*bgl2*-*gpdA*(p)::*clrB* mutant also dramatically decreased at the *amyR* expression level under carbon-free conditions. Therefore, *P*. *oxalicum amyR* (PDE_03964), an *Aspergillus oryzae amyR* homolog [41], was considered tightly associated with cellulolytic enzyme production. The strain with the deletion of *amyR* exhibited visible varying halos on cellulose and starch plates, as well as an identical phenotype on glucose relative to its parental strain (Fig 9A). As such, this strain demonstrated its differential roles in amylase and cellulase expressions. Moreover, this condition suggests that Δ*amyR* mutant has no defects in glucose uptake, sensing, or metabolism. The FPA in an *amyR* knockout mutant was about 1.6-fold higher than that in the wild-type strain of *P*. *oxalicum* (Fig 9B), while the *amyR* deletion reduced amylase under cellulose growth conditions (Fig 9C). Δ*amyR* mutant also displayed higher amounts of *cbh1* and *eg2* mRNA than that in the wild-type strain according to the results of northern blot (Fig 9D). Nonetheless, such mutant was deficient for transcribing the major glucoamylase gene *gluA* (PDE_09417) when grown on cellulose. This observation implies that AmyR was the main activator for amylase expression, and it repressed cellulase expression in response to the utilization of cellulose sources.

We constructed Δ*amyR-gpdA*(p)::*clrB* and Δ*amyR*-Δ*creA* mutants to investigate whether AmyR plays a negative role in the synergistic/additive transcriptional activation of cellulolytic genes. As predicted, both Δ*amyR-gpdA*(p)::*clrB* and Δ*amyR*-Δ*creA* mutants produced more cellulase activities than the strains that contain each individual mutation (Fig 9B) and showed higher transcription levels for cellulase genes under cellulose growth conditions (Fig 9E and 9F). The additive regulation for cellulase gene expression also existed in Δ*amyR*-Δ*xlnR* and Δ*amyR*-Δ*bgl2* mutants on cellulose (Fig 9G and S10A Fig). Therefore, the deletion of *amyR* in triple-mutant RE-10 (Δ*bgl2-*Δ*creA*-*gpdA*(p)::*clrB*) might further enhance cellulase expression under cellulose conditions. This premise also holds true in RE-27 and RE-29 mutants. Correspondingly, we constructed Δ*amyR*-Δ*bgl2*-Δ*creA*-*gpdA*(p)::*clrB* quadruple mutant (strain RE-30). However, the resulting strain RE-30 did not obtain greater cellulase expression than its parental triple-mutant RE-10 (S10B and S10C Fig), but AmyR still contributed to the functions of activating amylase expression in RE-10 on cellulose (S10D Fig). These results demonstrate that the deletions of *amyR* in RE-10 mutants were less effective for inducing cellulase expression than those in wild-type, *gpdA*(p)::*clrB*, Δ*creA*, and Δ*bgl2* strains on cellulose.

**Fig 10:**
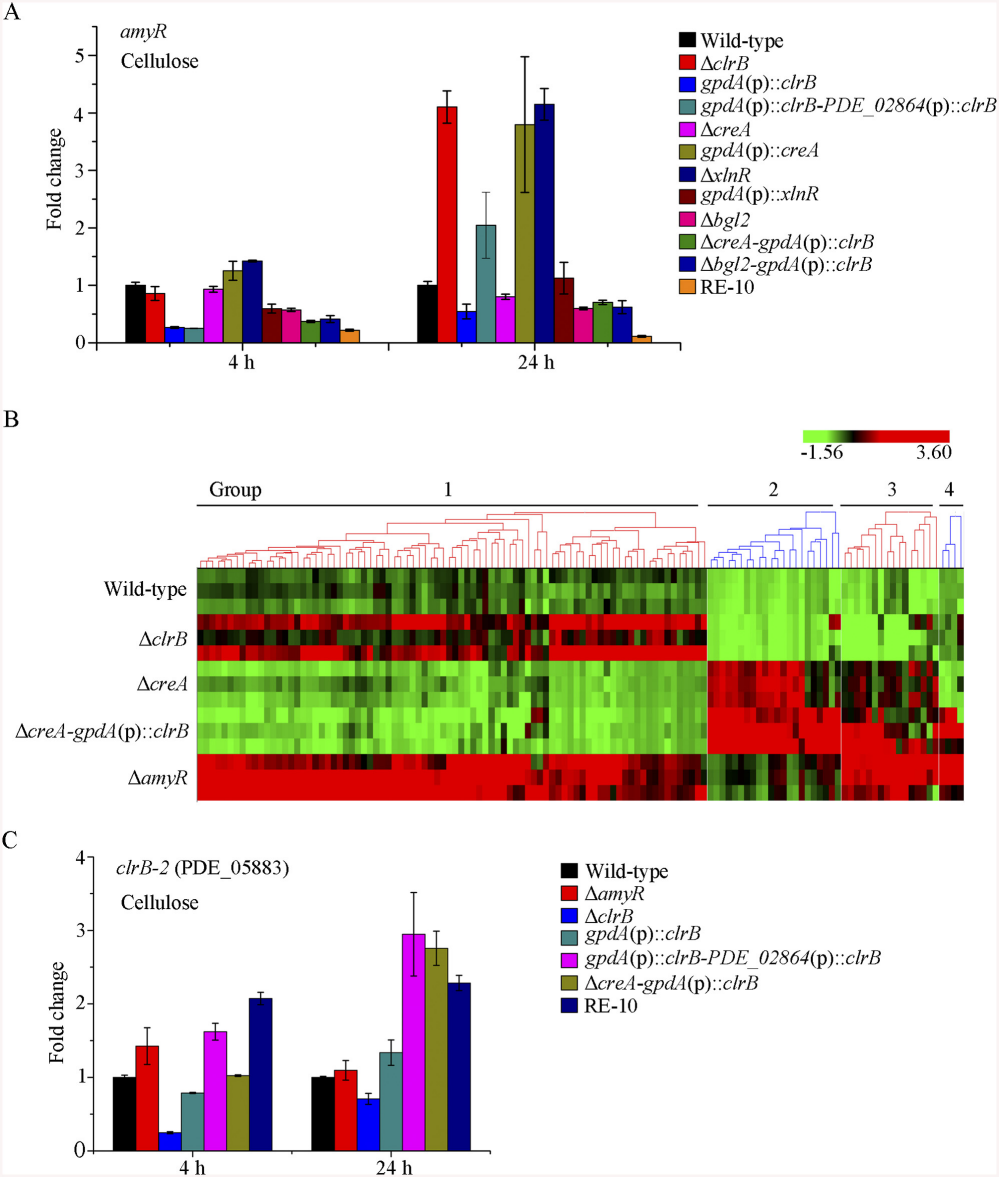
Expression of *amyR* was regulated by ClrB and CreA, and hierarchical clustering of RPKM for *amyR* regulon genes on cellulose. (A) The transcription levels for *amyR* in wild-type strain and mutants containing *bgl2*, *creA*, *clrB* or *xlnR* deletion were determined on cellulose by q-PCR. (B) Comparative analysis of RNA-seq data from Δ*amyR* and wild-type strains. Hierarchical clustering of RPKM for 131 AmyR-regulon genes was performed in Δ*amyR* mutant versus that in wild-type, Δ*clrB*, Δ*creA* and Δ*creA-gpdA*(p)::*clrB* mutants. (C) q-PCR measurements of PDE_05883 (*clrB-2*) in wild-type and the mutants after shift to cellulose for 4 and 24 hours. Expression levels were normalized to the wild-type.

The fact that RE-30 mutant produced cellulolytic enzymes near RE-10 implies that AmyR played a significantly different regulation activity for cellulase expression in RE-10 mutant than the wild-type strain under cellulose growth conditions. Considering the low expression of *amyR* in Δ*bgl2*-*gpdA*(p)::*clrB* mutant under carbon-free conditions (Fig 7D), we hypothesized that the transcriptional abundance for *amyR* was also downregulated in RE-10 on cellulose. As predicted, we first observed a significant decrease in Δ*creA-gpdA*(p)::*clrB* strain in RNA-seq data (RPKM: 124.5±5.8 in Δ*creA*, 20.0±3.1 in Δ*creA-gpdA*(p)::*clrB* for *amyR versus* 211.1±2.3 in the wild-type). The q-PCR experiments also revealed that the *amyR* expression was synergistically downregulated in the RE-10 mutant (Figs 7D and 10A). These data suggest that ClrB and CreA were supposed to participate in the control of the transcriptional response of *amyR* gene upon exposure to cellulose because the expression of *amyR* was decreased in *gpdA*(p)::*clrB* and Δ*creA* mutants, and increased in Δ*clrB* and *gpdA*(p)::*creA* mutants (Fig 10A). The deletion of *bgl2* also resulted in the decreased expression of *amyR*, which may also facilitate the decreased expression of *amyR* in Δ*creA-gpdA*(p)::*clrB* (Fig 10A). In other words, no differential expression for cellulase expression between RE-30 and RE-10 mutants may be tightly related to the dramatic deregulation of *amyR* in RE-10 (Figs 7D and 10A). By contrast, the synergistic increase of cellulase induction in Δ*creA-gpdA*(p)::*clrB* and RE-10 mutants may be partially a consequence of the decreased expression of *amyR*. The extent to which and how AmyR is involved in cellulase expression regulated by ClrB and CreA is still uncertain.

To gain insight into the molecular mechanism that underlies the AmyR-regulated cellulolytic gene expression on cellulose, we evaluated the global changes in Δ*amyR* mutants with three biological replicates by RNA-Seq. Consequently, 71% of the reads were mapped to the *P*. *oxalicum* 114–2 reference genome. The biological replicates of each Δ*amyR* mutant showed a high Pearson correlation (S6D Fig) and demonstrated the reliability of RNA-Seq. Given a |log2(fold change)|>1 and probability≥0.8 as the threshold, we determined that 131 genes (S7 Table) were upregulated and 579 genes (S8 Table) were downregulated in response to the deletion of *amyR* compared with the wild-type, respectively.

We then compared the RNA-Seq data for the 579 upregulated genes with that from the wild-type strain and Δ*amyR*, Δ*clrB*, Δ*creA*, and Δ*creA-gpdA*(p)::*clrB* mutants. The hierarchical clustering of these genes revealed nine groups of genes with similar expression patterns (S11 Fig). The expression levels for groups 1 and 2 increased in Δ*creA* and Δ*creA-gpdA*(p)::*clrB* mutants. Group 1 consisted of 195 genes. Within this subset, the proteins with dolichyl-diphosphooligosaccharide-protein glycotransferase activity (p = 3.0e-8) were enriched. Similarly, five genes in this group involved in starch degradation (i.e., PDE_04151, PDE_09417, PDE_05527, PDE_01201, and PDE_01354) were enriched. This case demonstrates that AmyR is the main activator for amylase gene expression. Group 2 composed of 122 genes. The GO-term analysis of these genes displayed a significant enrichment in the molecular function categories of the structural constituent of ribosome (p = 2.6e-101) and rRNA binding (p = 1.8e-5). These results signify that AmyR may play an important role in the translation process (p = 1.8e-38). Groups 3 to 6, 8, and 9 showed no statistically significant results with cutoff (FDR<0.05) via GO-term analyses. Group 7 contained 67 genes, in which 20 genes were enriched in the carboxylic acid metabolic process (p = 9.02e-12).

We also compared the RNA-Seq data for the 131 upregulated genes with that from the wild-type strain and Δ*amyR*, Δ*clrB*, Δ*creA*, and Δ*creA-gpdA*(p)::*clrB* mutants. The hierarchical clustering of these genes revealed four groups of genes with similar expression patterns (Fig 10B).

Group 1 included 84 genes (S7 Table), which were induced in Δ*clrB* mutant but repressed in Δ*creA* mutant, particularly in Δ*creA-gpdA*(p)::*clrB* mutant. The GO enrichment analysis revealed the induced expressions of the subsets of genes involved in the cellular amino acid metabolic (p = 5.4e-10) and organic acid biosynthetic processes (p = 1.6e-5). One gene cluster (from PDE_01212 to PDE_01220) encoding unclassified proteins was also involved in this dataset.

Group 2 consisted of 22 genes that were induced in Δ*creA* mutant (S7 Table), especially in Δ*creA-gpdA*(p)::*clrB* mutant. The GO function annotation of this subset genes revealed that the genes for hydrolase activity (p = 8.2e-12) constitute the largest group, including nine cellulase genes (i.e., PDE_07124, PDE_07945, PDE_05193, PDE_05633, PDE_09226, PDE_07929, PDE_00507, PDE_07928, and PDE_01261), five hemicellulase genes (i.e., PDE_06649, PDE_02101, PDE_09278, PDE_06023, and PDE_08094), β-glucosidase-encoding genes (i.e., PDE_00579 and *bgl2*), swollenin (i.e., PDE_02102), acetylesterase (i.e., PDE_05194), ABC multidrug transporter (i.e., PDE_07165), and formyltetrahydrofolate deformylase (i.e., PDE_07944). This group also contained cellodextrin transport-1-encoding genes (i.e., PDE_00607), a tetratricopeptide repeat protein (i.e., PDE_08095), and a hypothetical protein (i.e. PDE_06089).

Group 3 comprised 16 genes that were induced in Δ*creA* mutant (S7 Table), particularly in Δ*creA-gpdA*(p)::*clrB* and Δ*amyR* mutants. This gene set was categorized using GO terms. The results illustrated that the genes involved in the carbohydrate metabolic process (p = 1.06e-6) were enriched, including hemicellulases-encoding genes (i.e., PDE_01302, PDE_09710, and PDE_05998), endoglucanase (PDE_09969), β-glucosidase (PDE_04251), α-mannosyltransferase (PDE_09901), and α-xylosidase (PDE_06944). More importantly, a putative cellulose degradation regulator (PDE_05883) was observed within this set. The transcriptional expression for PDE_05883 was ClrB-dependent and repressed by CreA and AmyR. Moreover, this expression showed an additive increase in Δ*creA-gpdA*(p)::*clrB* under cellulose conditions (RPKM: 6.9±0.8 in Δ*clrB*, 35.3±1.4 in Δ*creA*, 3.4±1.1 in *ΔcreA-ΔclrB*, 62.9±9.6 in Δ*creA-gpdA*(p)::*clrB*, and 51.4±3.8 in Δ*amyR* for PDE_05883 *versus* 16.1±0.6 in the wild-type strain). The variations in the expression levels of PDE_05883 in these mutants were further identified via q-PCR experiments (Fig 10C). PDE_05883 encodes a conserved fungal Zn_2_Cys_6_ binuclear cluster domain with a significant amino acid homology with *N*. *crassa* cellulase essential regulator CLR-2 (NCU08042) for cellulose degradation. PDE_05883 shares 37% identity with CLR-2 in *N*. *crassa* (BioEdit, Expect = 5e-096) and a 45% identity with *P*. *oxalicum* ClrB (BioEdit, Expect = 5e-0163). Therefore, the gene of PDE_05883 was named as *clrB*-*2*, and the corresponding protein was called ClrB-2. The *P*. *oxalicum* closest homolog ClrB (BioEdit, Expect = e-131) of *N*. *crassa* CLR-2 was identified in this study. The function of ClrB involved in cellulose degradation was also extensively characterized in the preceding discussion. Nonetheless, only limited information has been reported about the molecular mechanism of this ClrB-2. In sum, the above findings provide an additional objective assessment of the role of AmyR in regulating cellulase expression. Likewise, the preceding analyses suggest that the combinatorial cross-regulation of ClrB, CreA, AmyR, and PDE_05883 defining a regulatory network of cellulase expression must be further characterized.

Group 4 consisted of four genes that were repressed in Δ*creA* mutant (S7 Table), but were significantly induced in Δ*creA-gpdA*(p)::*clrB* and Δ*amyR* mutants. These genes involved β-1, 6-glucanase-encoding genes (PDE_02004), succinate semialdehyde dehydrogenase (PDE_05599), 5-nitroimidazole antibiotic resistance protein (PDE_08743), and 4-aminobutyrate aminotransferase (PDE_09301).

### Direct interactions of the transcription factors ClrB, XlnR, CreA, and AmyR

**Fig 11:**
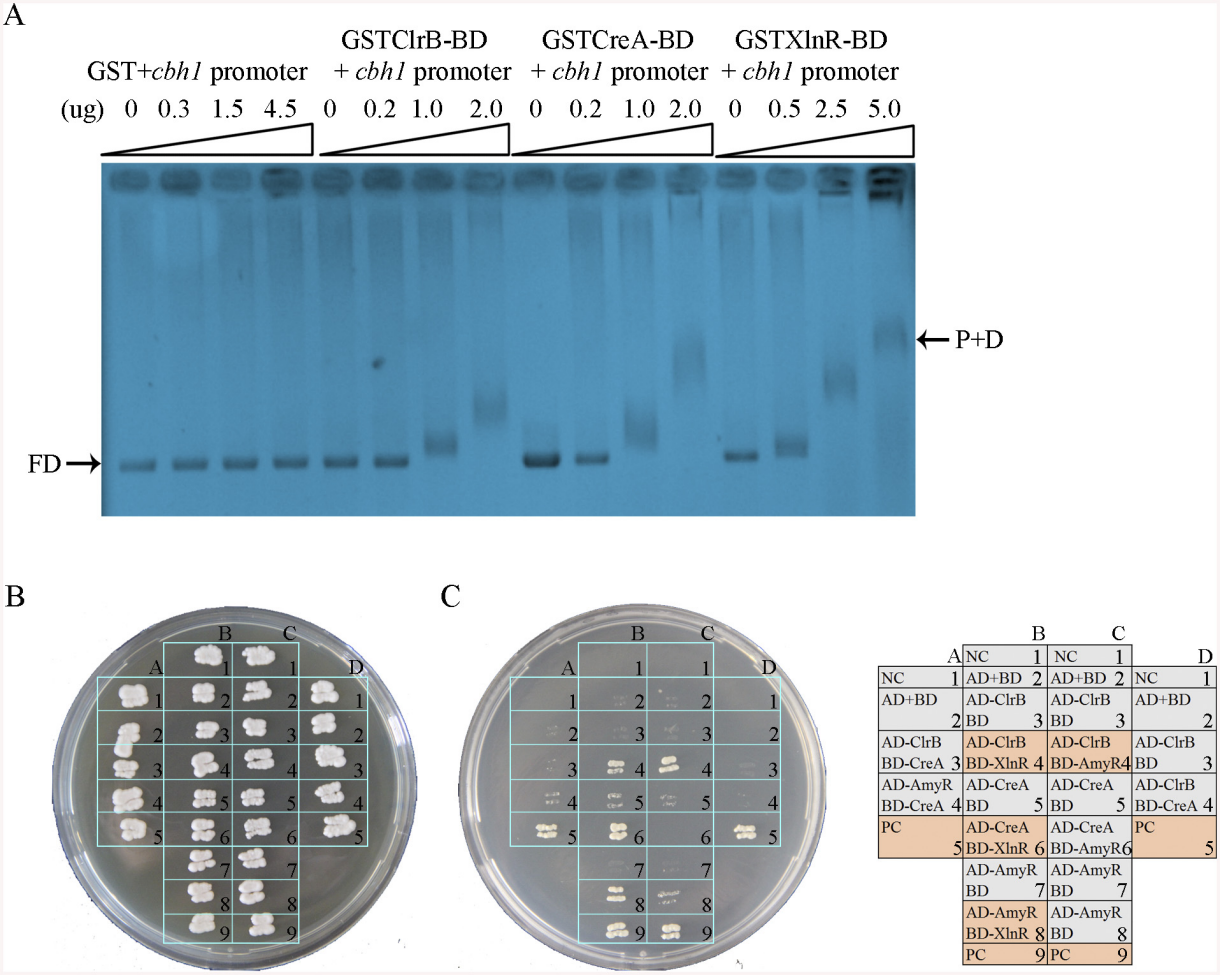
Electrophoretic mobility shift assay and protein interaction among ClrB, CreA, AmyR and XlnR. (A) Bandshifts showing GST-fusion ClrB-DNA-binding domain (GST ClrB-BD), GST-fusion CreA-DNA-binding domain (GST CreA-BD) and GST-fusion XlnR-DNA-binding domain (GST XlnR-BD) binding to *cbh1* promoter, compared to the GST negative control. FD, free DNA; P+D, protein-DNA complexes. (B) Yeast two-hybrid (Y2H) assay to detect interactions of ClrB with CreA, XlnR and AmyR on SD base medium including every essential amino acid. (C) Cells that contained positive direct interactions proteins for ClrB and XlnR (column, B4), CreA and XlnR (column, B6), AmyR and XlnR (column, B8) as well as ClrB and AmyR (column, C4) *in vitro* on quadruple dropout medium: SD/-Ade/-His/-Leu/-Trp. NC, negative control. PC, positive control.

The results of the transcriptome analyses, Northern blot, and q-PCR experiments specified above demonstrate that the core cellulolytic genes are tightly regulated by ClrB, CreA, AmyR, and XlnR transcription factors under inducing conditions. The fine-tuned regulation mechanisms allowed us to hypothesize that these central transcription factors may directly bind to the promoters of their core targets. This case was expected because the CreA and XlnR homologs in *T*. *reesei* [42] have been determined to be capable of binding to cellulase gene promoters, corresponding to *cbh1* in *P*. *oxalicum*. To support this hypothesis, GST-tagged ClrB (GST-ClrB), CreA (GST-CreA), and XlnR (GST-XlnR) binding domains were separately expressed in *Escherichia coli* and were purified. The nucleotide sequences of the putative target gene corresponded to 2 kb *cbh1* promoter fragment. The abilities of the recombinant proteins to bind to *cbh1* were assessed via electrophoretic mobility shift assay (EMSA). When the concentration of GST-ClrB, GST-CreA, or GST-XlnR fusion proteins increased, slower migrating shifted bands were evident (Fig 11A). However, no shifted band could be observed only with a high-concentration GST (negative control). These findings signify that ClrB, CreA, and XlnR could directly bind to *cbh1* promoter region, and the number of binding sites may be more than one.

An important question is whether the direct interactions associated with these regulators play important roles in regulating their target genes by assembling into active transcription complexes, in addition to their direct binding to DNA segments, as observed in *T*. *reesei* [42,43]. To investigate this possibility, the full-length open reading frames (ORFs) of the transcription factors ClrB, CreA, AmyR, and XlnR were PCR amplified using cDNA from *P*. *oxalicum* 114–2 as the templates. All amplicons were cloned into plasmid pGAD-T7 and separately obtained fusion proteins (i.e., AD-ClrB, AD-CreA, AD-XlnR, and AD-AmyR). Similarly, the full-length *creA*, *amyR*, and *xlnR* were cloned into the partner plasmid pGBK-T7 and resulted in BD-CreA, BD-AmyR, and BD-XlnR. Protein–protein interaction assay was performed. The results showed that the strains with interactions between ClrB and AmyR, XlnR and ClrB, XlnR and AmyR, and XlnR and CreA could grow on synthetic drop-out (SD) plates that lack Leu, Trp, and His (QDO, Clontech) (Fig 11B). Likewise, the results revealed that XlnR interacted directly with ClrB, CreA, and AmyR as well as with ClrB and AmyR *in vitro*.

## Discussion

Several conserved and essential transcription factors for cellulase and hemicellulase genes have been recently described in cellulolytic fungi; yet, the regulatory patterns of these factors have not been thoroughly analyzed [10,15–17,23]. In this study, a single-gene disruptant mutant library of the transcription regulators in *P*. *oxalicum* was constructed, and 20 transcription factors that play a pivotal role in the activation or inhibition of cellulose deconstruction were identified. Of these screened transcription factors, ClrB, CreA, XlnR, and AmyR were selected for further analyses. The cellulase expression regulated by additive cellulolytic effectors was observed. The results correspondingly provided comprehensive information for deciphering and redesigning a cellulase expression regulation network for rational engineering of cellulase hyper-producers.

### ClrB

In this study, a master transcription factor ClrB was exceptionally identified in *P*. *oxalicum* transcription factor mutant set screening. This key transcription factor positively regulated the cellulolytic gene expression, and its deletion strain exhibited dramatically reduced cellulase activities (Fig 1B). However, this factor was not strictly required for xylanase gene expression. The homology search of *P*. *oxalicum* ClrB within some fungal proteomes showed that the homologs of ClrB (i.e., Clr-2 in *N*.*crassa*, ClrB in *A*. *nidulans*, and ManR in *A*. *oryzae*) [16,17] were recently determined and were required to induce major cellulases, some major hemicellulases, and mannanolytic enzyme gene expression. The search of *T*. *reesei* protein databases via a Basic Local Alignment Search Tool using a ClrB/Clr-2 query revealed that a protein (Trire2: 26163) with low sequence identity existed. However, the homology search of *N*.*crassa* Clr-2 within *P*. *oxalicum* proteome illustrated that the protein sequences for ClrB (BioEdit, Expect = 5e-0163) and PDE_05883 (BioEdit, Expect = 5e-096) have 45% and 37% identity with Clr-2 sequence [17], respectively. These phenomena for two Clr-2 homologs in *P*. *oxalicum* proteome were not observed in *N*. *crassa* and *T*. *reesei* proteomes. This case suggests that differential-inducing mechanisms for cellulase expression may exist among cellulolytic fungi.

### XlnR

The homologs of regulator XlnR were the most conserved in cellulolytic fungi [10–12]. However, *P*. *oxalicum* XlnR does not have the same transcriptional inducing ability for cellulolytic and xylanolytic genes as in others [10–12]. Significant differences in these regulatory patterns for cellulase genes were observed in *P*. *oxalicum* Δ*xlnR* and *T*. *reesei* Δ*xyr1* mutants [10]. The lack of XlnR homolog in *T*. *reesei* eliminated cellulase expression, but not in *P*. *oxalicum* Δ*xlnR* mutant (Fig 2A and 2B). The deletion of *P*. *oxalicum xlnR* slightly reduced the transcript levels of some cellulases and abolished the major xylanase expression under induction conditions (Fig 2A), which were similar to that in *N*. *crassa* Δ*xlr-1* mutant [12]. These findings suggest that the transcriptional regulation of lignocellulose-degrading enzymes mediated by XlnR homologs may be highly conserved in various filamentous fungi, but may also have interesting differences.

### CreA

CreA/CRE1/CRE-1 is a wide-domain master regulator of carbon metabolism identified in filamentous fungi [7–9]. This regulator allows an organism to utilize a preferred carbon source but hinders it from metabolizing complex carbon sources, including cellulose [7–9]. In this study, the function of CreA homologs in repressing cellulolytic and xylanolytic gene expressions was conserved among cellulolytic fungi (Fig 2A and 2B). CreA homologs generally play an important role among cellulolytic fungi by linking CCR to developmental programs, including the conidia formation and hyphal morphology in *T*. *reesei* [23] and *P*. *oxalicum* (Fig 5B). This study determined that some transcription factors (i.e., PDE_07199, PDE_04095, and PDE_08372) are involved in both developmental programs and cellulase induction expression (Table 1). These regulators are conserved in cellulolytic fungi (Table 1). Therefore, the possible existence of an intimate crosstalk among certain developmental processes, such as sporulation and cellulase production pathways, is mediated by some regulators in ascomycete fungi.

### AmyR

By using RNA-seq data, we showed that expression of *amyR* was synergistically decreased in the Δ*creA*-*gpdA*(p)::*clrB* mutant. The lack of AmyR significantly induced cellulase expression and decreased the expression for amylase genes involved in starch degradation (Fig 9A–9C). As such, AmyR may control the balance between starch and cellulose utilization by inducing and/or repressing cellulolytic and amylolytic gene expressions in *P*. *oxalicum*, respectively. The multiple-sequence alignment analysis showed that *P*. *oxalicum* AmyR shares a weak homology with *N*. *crassa* COL26 (NCU07788, 23% sequence identity, E value = 2e-038 by BioEdit) [44] and *T*. *reesei* BglR (Trire2: 52368, 24% sequence identity, E value = 3e-031 by BioEdit) [18], but is highly homologous to *T*. *reesei*, a functionally uncharacterized Zn(II)_2_Cys_6_-type fungal-specific transcription factor (Trire2: 55105, 38% sequence identity, E value = 9e-095 by BioEdit). Interestingly, the regulatory functions of AmyR gene were distinct from those of *N*. *crassa* COL26 [44] and *T*. *reesei* BglR [18]. During its initial response to cellulose, *P*. *oxalicum* Δ*amyR* mutant exhibited induction and did not decrease the cellulolytic gene expression as in *N*. *crassa* Δ*col*-*26* mutant [44]. Moreover, *N*. *crassa* COL26 obviously repressed *cre-1* transcription to promote the relief of CCR [44], but the *creA* transcript abundance only slightly increased in *P*. *oxalicum* Δ*amyR* mutant. *N*. *crassa* Δ*col*-*26* mutant exhibited a severe growth defect on glucose, but not in *P*. *oxalicum* Δ*amyR* mutant. Although both *T*. *reesei* BglR [18] and *P*. *oxalicum* AmyR mutants displayed an elevated cellulase expression under inducing conditions, they demonstrated distinct regulatory trends to β-glucosidase expression. This difference might be related to the functional studies of BglR in *T*. *reesei* mutant PC-3-7 containing *bgl2* mutation and other uncharacterized mutations [18]. Nevertheless, *T*. *reesei* 55105 (Trire2), which is much more homologous to *P*. *oxalicum* AmyR than *T*. *reesei* BglR (Trire2: 52368), may be a candidate regulator involved in cellulase expression regulation.

### Cascade regulation for cellulolytic genes

**Fig 12:**
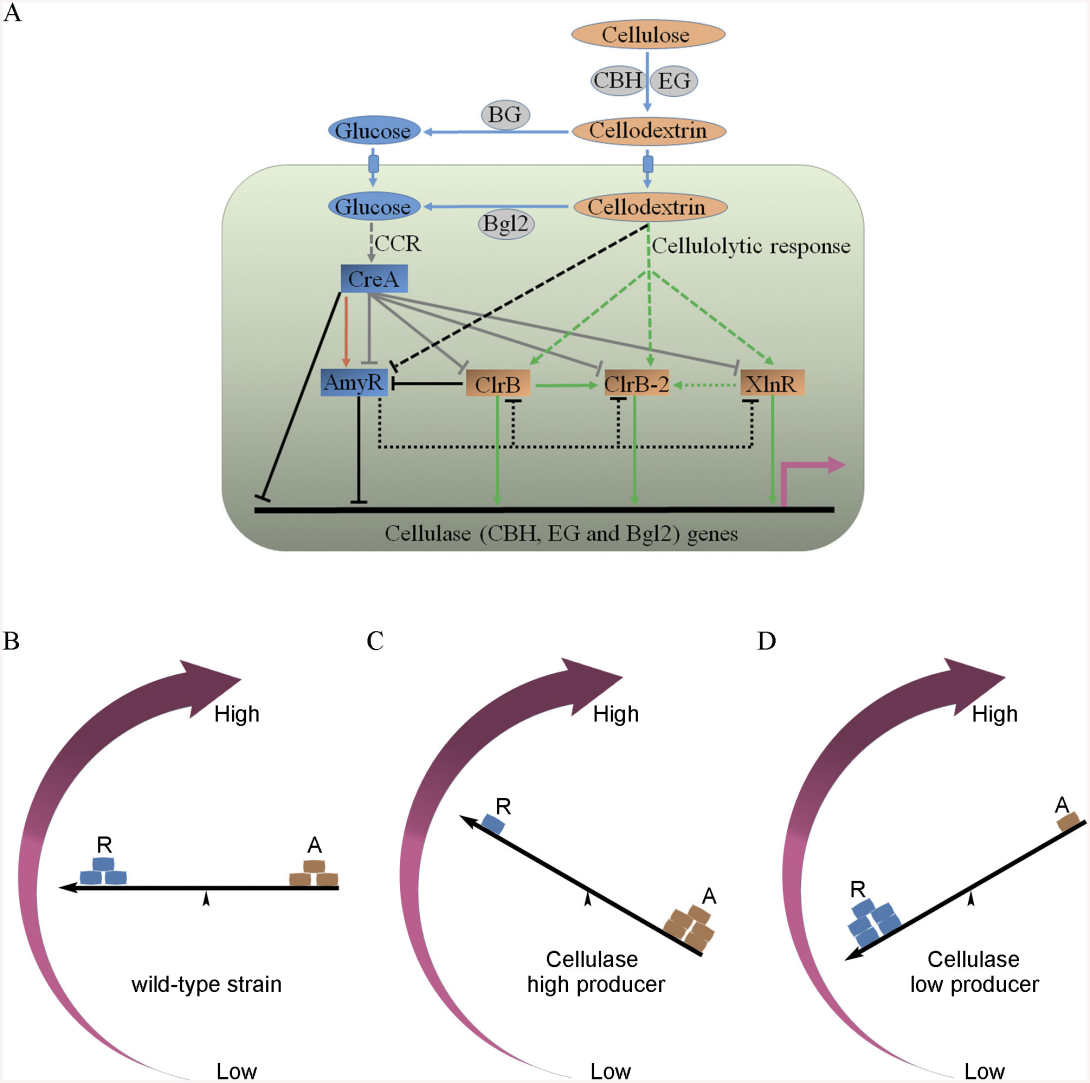
Model of regulation of cellulase expression by ClrB, CreA, AmyR, XlnR and ClrB-2 in *P*. *oxalicum*. (A) Under cellulose growth conditions, cellulolytic inducers (cellodextrins) were liberated from cellulose by cellulase. Lack of the major intracellular beta-glucosidase Bgl2 facilitates the accumulation of intracellular cellodextrins, which can trigger signaling cascades that include expression of cellulase genes repressed by CreA and AmyR and activated by ClrB, XlnR and ClrB-2. Cellulase gene expression was synergistically enhanced by simultaneous overexpression of ClrB and lack of CreA, and additively increased in Δ*amyR-gpdA*(p)::*clrB*, Δ*amyR*-Δ*creA* and *gpdA*(p)::*clrB*-*gpdA*(p)::*xlnR* mutants. However, transcriptional regulation for cellulolytic regulator genes is also an important and powerful part of the regulatory network of cellulase gene expression. In the early cellulolytic induction, ClrB functions to repress expression of *amyR*, whose expression level is downregulated in Δ*creA*-*gpdA*(p)::*clrB* mutant and further in Δ*bgl2*-Δ*creA*-*gpdA*(p)::*clrB* mutant, and thus is activated by CreA (brown arrow) and reduced in Bgl2-deficient background. AmyR may also play a role in repressing *clrB*, *xlnR* and *clrB-2* expression on cellulose (dotted line). In addition, the novel cellulolytic regulator gene *clrB-2* expression is induced by ClrB and may be enhanced by XlnR. Moreover, transcriptional expression for ClrB, AmyR, XlnR and ClrB-2 genes is also repressed by CCR mediated by CreA in the presence of glucose (gray plunger). For each regulatory step, arrow means positive control, and plunger means negative control. (B) A “seesaw model” understanding the regulation of the cellulolytic system in transcription levels for the cellulase genes in the *P*. *oxalicum* wild-type strain. “R” means repressor, and “A” means activator. “←” means promoters for cellulase genes. (C) A “seesaw model” shows the transcription levels for cellulase genes increase by enhancing the positive regulation of activators or/and reducing the negative control of repressors (i.e. Δ*creA*, Δ*amyR*, *gpdA*(p)::*clrB*, *gpdA*(p)::*clrB*-*PDE_02864*(p)::*clrB*, *gpdA*(p)::*xlnR*, *gpdA*(p)::*xlnR*-*gpdA*(p)::*clrB*, Δ*creA*-*gpdA*(p)::*clrB*, RE-10, RE-27 and RE-29). A “seesaw model” in (D) shows the transcription levels for cellulase genes reduce by decreasing the positive regulation of activators or/and increasing the negative regulation of repressors (i.e. Δ*clrB*, Δ*xlnR*, Δ*clrB-*Δ*xlnR* and *gpdA*(p)::*creA*).

The cellulase expression in *P*. *oxalicum* is a highly coordinated process regulated by a suite of cellulolytic transcription factors (i.e., ClrB, CreA, XlnR, AmyR and ClrB-2) and other novel uncharacterized regulators. In this study, the cellulolytic regulators ClrB, XlnR, AmyR and ClrB-2 were significantly regulated at the transcriptional levels during their growth on glucose, but slightly at the early phase for under cellulose growth conditions (Figs 5A and 12A). The CreA tightly regulated the expression of *clrB*, *xlnR*, *amyR* and *clrB-2* in response to environmental carbon. These data suggested that CreA might have a cascade regulation because it repressed the activator genes for AmyR, ClrB, ClrB-2 and XlnR as well as the structural genes whose expression was upregulated by ClrB, XlnR and ClrB-2 (Fig 12A). This “double-lock” regulation of cellulolytic genes mediated by regulator homologues in cellulolytic fungi might be general, which could facilitate the fast conversion of carbon metabolism from favored carbon sources to cellulose and hemicellulose utilization. This cascade regulation mechanism mediated by *P*. *oxalicum* CreA was similar to the pathway described Cre1-mediated double repression of *xyr1* and *xyn1* in *H*. *jecorina* [10]. Some similar situations with regard to the repression of the transcription of *A*. *nidulans* ethanol and xylan regulons have been previously reported [45,46]. The deletion of *creA* resulted in the slightly decreased expression of *amyR* (Fig 10A), whose absence further led to the upregulation of cellulase genes (Fig 12A). Similarly, the overexpression of ClrB led to a decreased expression of *amyR*, whose expression level was synergistically downregulated in RE-10 mutant, but increased in *gpdA*(p)::*clrB*-*PDE_02864*(p)::*clrB* mutant (Fig 10A). These data implied that regulatory function of ClrB and CreA on *amyR* expression may be important for cascade regulation for cellulolytic genes under cellulose growth conditions.

Another key finding of this study is that the transcriptional expression of ClrB-2, a novel regulator, is responsive to ClrB, XlnR, CreA and AmyR, which implied that ClrB-2 may mediate the cascade transcriptional regulation for cellulolytic genes by ClrB, XlnR, CreA and AmyR (Figs 5A, 10C and 12A). We did not systematically determine how cellulolytic genes could transform in *clrB*-*2* mutant yet. Nonetheless, the variable expression levels on cellulose in these regulator mutants suggest that ClrB-2 is one of the most interesting target in *P*. *oxalicum* cellulolytic regulatory networks. Whether ClrB, CreA, XlnR, and AmyR converge to exert their partial regulatory function via ClrB-2 on cellulolytic gene expression must be urgently elucidated.

### Synergistic and dose-controlled regulation by cellulolytic regulators

The synergistic and collective regulations of cellulase expression by cellulolytic regulators are still elusive and largely uncharacterized in cellulolytic fungi. Thus far, no research has systematically investigated whether or how the most central cellulolytic factors CreA and ClrB homologs perform the synergistic regulation of cellulase genes. In this study, Δ*creA*-*gpdA*(p)::*clrB* strain yielded strong synergistic effects on cellulase expression (Figs 1A–1E, 2A and 2B). This observation indicates that the full induction of cellulase genes requires not only an exclusive inducer-induction and activation by positive regulators, but also the release of negative transcription factors. In accordance to these synergistic regulatory mechanisms in *P*. *oxalicum*, we constructed significantly higher cellulase hyper-producer RE-27 and RE-29 than the triple-mutant RE-10 (Fig 8A and 8B). We believe that the RERN technology presented here will be a valuable contribution in transforming some non-industrial model species (e.g., *N*. *crassa* and *A*. *nidulans*) into more industrially relevant species.

A very interesting finding in this study is that the cellulase expression increased evidently accompany with the increase of the copy number and the efficiency of its promoter of *clrB* or *xlnR* gene (Fig 8A and 8B, and S3A–S3D Fig), indicating that a tunable cellulase expression may be controlled by the activator concentration under cellulose conditions. These data signify that the cellulase expression is not only dependent on the presence of the activators ClrB and XlnR, but also severely dependent on their dose effects of ClrB and XlnR transcriptional abundances.

The other issue that must be explored is how cross-correlations occur between the cellulolytic regulators. To evaluate the role of CreA in CreA-mediated repression of cellulase and hemicellulase gene expressions, we developed three possible hypotheses. a) Putative CreA binding sites overlap with the putative ClrB or XlnR binding sites, and CreA could preferentially bind to the sites and/or block their binding to target promoters by competition. A recent study identified the presence of putative cis-regulatory elements recognized by both XYR1 and CRE1 and spaced in XYR1- and CRE1-dependent cellulase gene promoters [42]. b) CreA could stably associate with ClrB or XlnR components to form a heterocomplex, thereby making ClrB and XlnR completely non-functional for the induction of cellulolytic and xylanolytic genes. In this study, CreA and XlnR were assumed to directly interact with each other according to the yeast two-hybrid assay (Fig 11B and 11C). c) The activity of transcription factor is influenced by intracellular protein post-translational modifications, such as phosphorylation. These modifications may influence the ability of the transcription factor to bind to its binding sites [47]. In sum, the findings presented above support our proposition that biological relevances may exist between CreA and ClrB as well as between CreA and XlnR, which tightly regulate the expression of cellulase and hemicellulase genes.

### Cellulase regulatory network sensitive to inducers in the intracellular environments

Beta-glucosidases are the conserved components required in cellulose deconstruction, the number of which significantly varies among the genomes of cellulolytic fungi [24,48,49]. The lack of *P*. *oxalicum* intracellular Bgl2 contributes to the increase of cellulase expression [27], but not in *T*. *reesei* [50] and *N*. *crassa* [51]. These findings have raised the question as to how Bgl2 mediates the carbon metabolism involved in signal cascades in relation to the regulation of cellulase gene expression, which may reflect the general trend of cellobiose/cellodextrins-induced cellulase expression in cellulolytic fungi [27,50,51]. Some β-glucosidases in *T*. *reesei* [50] and *N*. *crassa*. [51] have been observed to play important roles in balancing cellobiose production and metabolism in intra- and extra-cellular environments. Given the general phenomenon that cellobiose induces cellulase expression in cellulolytic fungi [27,50,51], *P*. *oxalicum* Δ*bgl2*-*gpdA*(p)::*clrB* mutant showed a strongly elevated cellulase expression (Fig 7A and 7B), which could be partially ascribed to the signal induction cascade mediated by cellobiose/cellodextrins from cellulose (Fig 12A). The robust induction of cellulase expression in Δ*bgl2*-Δ*creA* mutant was remarkably greater than that in each deletion mutant on cellulose (Fig 7A and 7B). This observation supports the premise that the CCR mediated by CreA increased when the major predicted intracellular β-glucosidase was absent under cellulose growth conditions. Although the lack of Cre-1 in *N*. *crassa* triple β-glucosidase mutant showed higher concentrations of secreted active cellulases than that in wild-type strain on cellobiose, it did not facilitate protein production and cellulase induction on cellulose [51]. In sum, ClrB positively regulated the transcriptional expression of *bgl2* (S5 Fig), but its deletion conversely enhanced the signal cascade activation regulation by ClrB and/or the repression regulation of CCR mediated by CreA (Figs 7A, 7B and 12A). In addition, we also determined that the cellulase expression in Δ*bgl2-*Δ*amyR* double mutant was further enhanced compared with each individual deletion strain (S10A Fig). These results revealed that the functional regulations for cellulase expression by these cellulolytic regulators may be sensitive to inducers in intracellular environments. This finding implies that the combination of intracellular cellodextrin induction and redesigned cellulolytic transcription factor regulation in *P*. *oxalicum* may be general for the full induction of cellulase expression on cellulose.

### Inducer-free cellulase expression in cellulolytic fungi

Many recent studies have attempted to robustly produce cellulases without inducers, but the molecular mechanism of cellulase induction under non-inducing conditions has remained elusive in diverse cellulolytic species [20,40]. Recently published data also showed that the misexpression of *N*. *crassa clr-2* through P*ccg*-1 [20] and P*tcu1*-driven expression of *xyr1* [40] was sufficient for inducer-free cellulase expression. However, the cellulolytic gene transcript between *P*. *oxalicum gpdA*(p)::*clrB* and wild-type strains had no obvious differences under non-inducing conditions. Such case was similar in the XlnR activator in the *gpdA*(p)::*xlnR* strain under CreA-mediated CCR, and *cbh1* transcription was still remarkably in low-level expression in the Δ*creA* mutant on a carbon-free medium, which indicates the full induction requirement of cellulase gene expression, as evident in *T*. *reesei* [25] or *N*. *crassa* [51]. However, the cellulolytic gene transcription levels were obviously upregulated in the Δ*creA*-*gpdA*(p)::*clrB* mutant under repressing conditions. These findings implied that the lack of CreA contributed to the activating function for ClrB on cellulolytic genes even in glucose. Notably, the Δ*bgl2*-*gpdA*(p)::*clrB* strain exhibited an induction transcription of core cellulase genes for several orders of magnitude increase than the wild-type strain that shifted to a carbon-free medium (Fig 7C and S7C Fig). No bulk of cellodextrins was apparently transported into the cell to induce cellulase gene expression when subjected to starvation, but the induction abilities of these core cellulase regulons in the Δ*bgl2*-*gpdA*(p)::*clrB* mutant under non-inducing conditions were comparable to under cellulose conditions (Fig 7C and S7C Fig). The *amyR* expression level had a 7.7-fold decrease, *clrB* had a 17.5-fold increase, and *clrB*-*2* had a 9.3-fold increase in the Δ*bgl2*-*gpdA*(p)::*clrB* mutant versus wild-type strain under carbon-free conditions (Fig 7D), which implied that AmyR, ClrB, and ClrB-2 were possibly tightly involved in cellulase expression regulatory during energy abstinence, as well as that under cellulose growth conditions. These were novel findings that indicated the “starvation response” for cellulase genes in *P*. *oxalicum* and other diverse cellulolytic fungi.

### Conclusion

Cellulase formation apparently occurred because of consistent respective regulators, including the characterized or novel transcription factors identified in *P*. *oxalicum* in this context. Cellulase synergistic and dose-controlled regulatory systems mediated by diverse cellulolytic effectors were observed in the system mutation of this study for *P*. *oxalicum* regulators. Using this model (Fig 12A), the accumulation of intracellular cellodextrins can trigger signaling cascades that include expression of cellulase genes repressed by CreA and AmyR and activated by ClrB and XlnR. However, our data also support that the transcriptional regulation for CreA, AmyR, ClrB and XlnR genes is a powerful part of the regulatory network of cellulase gene expression. In the early cellulolytic induction, ClrB functions to repress expression of *amyR*, whose expression level is activated by CreA and reduced in Bgl2-deficient background (Fig 12A). Moreover, transcriptional expression for ClrB, AmyR, XlnR and ClrB-2 genes is also significantly repressed by CCR mediated by CreA in the presence of glucose (Fig 12A). The data established ClrB as a focal point to regulate cellulase expression by integrating other regulators and the target genes of these regulators, which refined our understanding on transcriptional regulatory network as a “seesaw model” in which coordinated regulation of cellulolytic genes was established through activators and repressors counteraction (Fig 12B–12D). These observations also suggested the hypotheses that the rational design of cellulase or high-value protein super producers might be guided in the future for the combinatorial effects of diverse cellulolytic effectors.

## Materials and Methods

### *Penicillium oxalicum* strains, media, and growth conditions

All the strains used in this study are listed in the S9 Table and are grown on Vogel’s medium that contain 2% glucose (mass/volume percent), unless otherwise noted. The *P*. *oxalicum* wild-type strain 114–2 (CGMCC 5302) was used as parental strain throughout this study. Hygromycin B, pyrithiamine, phosphinothricin, and sulfonylurea were added to the media with final concentrations of 200, 300, 1.6, and 4 μg/mL used for transformant selection, respectively. Vogel’s 50x salts (1,000 mL) was used: 125 g Na_3_Citrate•2H_2_O, 250 g KH_2_PO_4_, 100 g NH_4_NO_3_, 10 g MgSO_4_•7H_2_O, 5 g CaCl_2_•2H_2_O, 0.25 mg biotin, and 5 ml trace element solution (5 g Citric acid•H_2_O, 5 g ZnSO4•7H_2_O, 1 g Fe(NH_4_)_2_(SO_4_)_2_•6H_2_O, 0.25 g CuSO_4_•5H_2_O, 0.05 g MnSO_4_•1H_2_O, 0.05 g H_3_BO_3_, and 0.05 g Na_2_MoO_4_•2H_2_O, which were dissolved in distilled water; the resulting total volume was 100 mL). The wheat bran medium (mass/volume percent) was composed of corn cob residue (2.0%), Avicel (0.6%), wheat bran (4.6%), soybean cake powder (1.0%), (NH_4_)_2_SO_4_ (0.2%), NaNO_3_ (0.28%), urea (0.1%), KH_2_PO_4_ (0.3%), and MgSO_4_ (0.05%). Vogel’s medium that contained 1 M sorbitol was used in all the transformation experiments. Vogel’s medium with 2% Avicel was used as a sole carbon source to induce cellulase expression for cellulase activity assays, q-PCR, Northern blot, or RNA-seq analyses. The fluid mediums of the *P*. *oxalicum* strains were all cultivated in Erlenmeyer flasks at 30°C in constant light and 200 rpm agitation rate. The cultivation was performed on plates by adding agar in the fluid medium as a solidifying agent at 30°C in constant light.

### Protoplast preparation and transformation

*P*. *oxalicum* protoplast prepared according to modified methods, as described by Gruber et al. [52]. Seven to eight PDA plates were prepared, and 50 μL of fresh spore solution was streaked out on every cellophane-covered PDA plate. The plates were incubated at 30°C for 11–12 hours. Three milliliters of this protoplasting solution [0.075 g lysing enzymes up to 25 mL solution A (1.2 M sorbitol and 0.1 M KH_2_PO_4_, and pH = 5.6] were pipetted into a sterile petri dish, and one cellophane disc with freshly grown mycelium was added. Subsequently, 3 mL of protoplasting solution was readded. The petri dish was incubated at 30°C for 150 min. The final protoplast suspension was filtered into a sterile 50 mL centrifuge tube through a lens cleaning tissue in a glass funnel. The suspension was centrifuged for 10 min at 2000 rpm and 4°C in a swing-out rotor. The supernatant was cautiously decanted, and the pellet was resuspended in 10 mL of solution B (1 M sorbitol, 50 mM CaCl_2_, 10 mM TrisHCl, and pH = 7.5). The suspension was recentrifuged for 10 min at 2000 rpm and 4°C, and then the supernatant was cautiously decanted and the protoplasts were resuspended in 0.5 mL of solution B. The protoplasts were stored in ice. The following components: 200 μL protoplast suspension, 10 μL DNA fragment, and 50 μL of solution C (25% PEG 6000, 50 mM CaCl_2_, 10 mM TrisHCl, and pH = 7.5), were added into a 10 mL centrifuge tube and were mixed gently, and then the mixture was incubated on ice for 20 min. Two milliliters of solution C (room temperature) were added to the transformation mixture, which was mixed gently. The mixture was incubated for 5 min at room temperature, and then 4 mL of solution B was added and was mixed gently. All the transformation mixture was added to 30 mL Vogel’s medium (55°C) that contained hygromycin B, pyrithiamine, phosphinothricin, or sulfonylurea, and was then mixed shortly and was poured onto the bottom of the Vogel’s medium. Transformants would be visible for 3–4 days for hygromycin B, pyrithiamine, or phosphinothricin, and for 6–8 days for sulfonylurea at 30°C.

### High throughput generation of 470 regulator deletion mutants

The *P*. *oxalicum* genome is available in DDBJ/EMBL/GenBank (under the accession number AGIH00000000) or at http://genome.jgi.doe.gov/Penox1/Penox1.home.html. Transcription factors were identified and annotated according to InterPro IDs in the Fungal Transcription Factor Database [53]. The transcription factor deletion strains, where each coding region of the transcription factor was substituted with a selective marker *ptra* gene [31], were constructed from the *P*. *oxalicum* strain Δ*pku70*::*hph* [33] as follows. Deletion cassettes conferring resistance to pyrithiamine hydrobromide were constructed according to the double-joint PCR strategy [32]. Primer pairs for (x)-F1+(x)-ptraR and (x)-ptraF+(x)-R1 (S1 Table) were designed using the Primer 5 software, and the pairs were used to amplify the upstream and downstream fragments for 1000–1500 bp on either side of the encoding regions for each single target gene. The *ptra* selectable marker cassette was PCR-amplified from the pME2892 plasmid [54] with the PtraF1+PtraR1 (S1 Table) primer pair and was PCR product-purified. The upstream and downstream fragments contained 25 bp homology to the *ptra* cassette sequence. The primer pair (x)-F2+(x)-R2 (S1 Table) was used to produce final deletion cassettes through double-joint fusion PCR [the program used to fuse the three fragments: 94°C 2 min for 10 cycles (94°C for 30 s, 58°C for 10 min, and 72°C for 3 min), 72°C 5 min] [32]. PCR experiments were performed in final volumes of 50|μL that contained 1 unit of Trans HIFI DNA polymerase, 0.2|mM dNTP, and 0.4|μM of each primer. The program used to amplify the fused knockout cassettes was as follows: one cycle of 94°C (120 s), 30 cycles of 94°C (30 s), 58°C (30 s), and 72°C (1 min for every 1 kb of amplified product), followed by a final 10|min at 72°C. All the PCRs were performed in a Bio-Rad DNA Engine Peltier Thermal Cycler.

Each knockout cassette was independently transformed into *P*. *oxalicum* strain Δ*pku70*::*hph* protoplasts [33]. The mature transformant conidia from the Vogel’s medium slant was streaked onto a Vogel’s medium plate that contained pyrithiamine hydrobromide. At least three Ptra-resistant colonies obtained from each assay were analyzed through diagnostic PCR to confirm the deletion using the primer pairs (x)-F1+ptraYZR or ptraYZF+(x)-R1 (S1 Table). Within the pair, the primer (x)-F1 or (x)-R1 (S1 Table) was located outside the transforming deletion fragment of the genome, and the primer ptraYZR or ptraYZF (S1 Table) was unique to the *ptra* sequence. These obtained strains constituted a stock of the *P*. *oxalicum* transcription factor deletion mutant.

### Gene complementation

The complementation cassette that conferred resistance to hygromycin B was used to transform into the corresponding mutant to identify that the interesting phenotype(s) observed for the transcription factor gene deletion mutants was indeed caused by the deletion of a relevant gene. The upstream and downstream fragments contained 25 bp homology to the *hph* cassette sequence. *clrB* and *amyR* wild-type allele complementation cassettes were obtained by amplifying the upstream fragments (encompassing the 1.5 kb promoter, the open reading frame, and 0.5 kb 3’ untranslated region) using the primer pairs clrB-F1+clrBHPH-R and AmyR-F1+AmyRHPH-R (S1 Table), respectively. The 1.5 kb downstream flanking segments of the 3’ untranslated region from the *P*. *oxalicum* genome DNA were amplified through the primer pairs clrBHPH-F+clrB-R1 and AmyRHPH-F+AmyR-R1 (S1 Table). The 1.8|kb *hph* gene fragment was amplified from the pSilent-1 plasmid [55] with the primers Hphs-F and Hphs-R (S1 Table). These three PCR fragments were ligated through Double-joint PCR and were amplified through the nest primer pairs clrB-F2+clrB-R2 and AmyR-F2+AmyR-R2 (S1 Table). The resulting *clrB* and *amyR* complementation cassettes were transformed into Δ*clrB*::*ptra* and Δ*amyR*::*ptra* mutants, and the complementation strains R*clrB* and R*amyR* of these cassettes were obtained, respectively.

### Construction of *gpdA*(p)::*clrB*, *gpdA*(p)::*creA*, *gpdA*(p)::*creA*-*gpdA*(p)::*clrB*, and *gpdA*(p)::*xlnR* mutants

The *clrB* promoter was replaced with the *gpdA* (glyceraldehyde-3-phosphate dehydrogenase) promoter from *A*. *nidulans* [37]. Moreover, 1,314 bp of the *gpdA* promoter was amplified from the plasmid pAN7-1 [56] using the primers PgpdA-F1 and PgpdA-R1 (S1 Table). Subsequently, 2,008 bp *ptra* selectable marker cassette was PCR-amplified with the primer pair PtraF1+PtraR1 (S1 Table) and was PCR product-purified. Furthermore, 3,148 bp of the *clrB* open reading frame and 3’ untranslated region were amplified with the primer pair clrB-Fa+clrB-Ra (S1 Table), and this fragment overlapped with the *gpdA* promoter and *ptra* fragment by 25|bp at the ends of this fragment. These 6375 bp PCR products were then ligated in the order of *gpdA*(p)::*clrB*-*ptra* by splicing through double-joint PCR with the nest primers PgpdA-F2 and PtraR1 (S1 Table).

A similar strategy was used to construct the *creA* overexpression cassette under the influence of the *P*. *oxalicum gpdA* promoter. Moreover, 1749 bp of the *gpdA* promoter was amplified from the *P*. *oxalicum* 114–2 genome DNA through the primers PGP-F1 and PGP-R (S1 Table). In addition, 1890 bp of the *hph* cassette was amplified from the plasmid pSilent-1 [55] with the primers Hphs-F and Hphs-R (S1 Table). Moreover, 1743 bp of the *creA* open reading frame and 3’ untranslated region was amplified using the primer pair GPcre-F+GPcre-R (S1 Table), and this fragment overlapped with the *gpdA* promoter and *hph* fragment by 25|bp at the ends of this fragment. These PCR products were then ligated in the order of *gpdA*(p)::*creA*-*hph* by splicing through double-joint PCR with the primers PGP-F2 and HPH-R1 (S1 Table). The resulting 5248 bp *gpdA*(p)::*creA*-*hph* overexpression cassette was transformed into *P*. *oxalicum* wild-type and *gpdA*(p)::*clrB*-*ptra* strain protoplasts, and the *gpdA*(p)::*creA* and *gpdA*(p)::*creA*-*gpdA*(p)::*clrB* mutants were obtained, respectively.

Similarly, the *xlnR* promoter was replaced with the *gpdA* promoter from *A*. *nidulans*. Moreover, 1314 bp of the *gpdA* promoter was amplified from the plasmid pAN7-1 [56] through the primer pair PgpdA-F1 and PgpdA-R1 (S1 Table). In addition, 1890 bp of the *hph* cassette was amplified from the plasmid pSilent-1 [55]. The *xlnR* open reading frames and 3’ untranslated region were amplified with the primer pair XlnR-Fa+XlnR-RH2 (S1 Table), and this fragment overlapped with the *gpdA* promoter and the *hph* fragment by 25|bp at the ends of this fragment. These PCR products were then ligated in the order of *gpdA*(p)-*xlnR*-*hph* by splicing through double-joint PCR with the primers PgpdA-F2 and Hphs-R1 (S1 Table). The resulting 6675 bp *gpdA*(p)::*xlnR*-*hph* overexpression cassette was transformed into *gpdA*(p)::*clrB* strain protoplasts.

The resulting *gpdA*(p)::*clrB*-*ptra*, *gpdA*(p)::*creA*-*hph*, and *gpdA*(p)::*xlnR*-*hph* overexpression cassettes were used to transform *P*. *oxalicum* wild-type strain protoplasts. The *gpdA*(p)::*creA*-*ptra*, *gpdA*(p)::*clrB-ptra*, and *gpdA*(p)::*xlnR*-*hph* overexpression mutants were selected on Vogel’s medium plate that contained hygromycin B or pyrithiamine hydrobromide.

### Construction of the Δ*creA*, Δ*creA-gpdA*(p)::*clrB*, and Δ*creA-*Δ*clrB* mutants

Double-joint PCR was performed to construct the *creA* knockout cassette, with the *hph* cassette flanked by 1.5 kb upstream (CreA-F1+Crehph-R) (S1 Table) and 1.5 kb downstream (Crehph-F+CreA-R1) (S1 Table) of the *creA* ORF. Moreover, 1.8 kb of the *hph* cassette was amplified from the plasmid pSilent-1 [55]. The Δ*creA*::*hph* final deletion cassette fragment was obtained through the primer pair (CreA-F2+CreA-R2) (S1 Table) with the three fragments above used as a PCR template. The Δ*creA*::*hph* cassettes were transformed into *P*. *oxalicum* wild-type, *gpdA*(p)::*clrB*-*ptra*, and Δ*clrB* strain protoplasts. The selection of the Δ*creA*, Δ*creA-gpdA*(p)::*clrB*, and Δ*creA-*Δ*clrB* transformants was consistent with the previous study.

### Construction of the Δ*xlnR*, Δ*xlnR*-Δ*clrB*, and *gpdA*(p)::*clrB*-*gpdA*(p)::*xlnR* mutants

The Δ*xlnR* cassettes conferred resistance to hygromycin B. The *hph* selectable marker cassette was PCR-amplified with the primers Hph-F1 and Hph-R1 (S1 Table) from the plasmid pAN7-1 [56]. The upstream and downstream flanking fragments were amplified with the primer pairs XlnR-F1+XyrHph-R and HphXyr-F+XlnR-R1 (S1 Table), which contained 25 bp homology to the *hph* cassette sequence. The primer pair XlnR-F2 and XlnR-R2 (S1 Table) was used to produce final deletion cassettes through a double-joint fusion PCR. The resulting Δ*xlnR*::*hph* knockout cassette was used to transform wild-type protoplasts, and the *P*. *oxalicum* Δ*xlnR* mutant was obtained. The Δ*xlnR*::*hph* knockout cassette was used to transform the Δ*clrB* protoplasts, and then the Δ*xlnR*-Δ*clrB* mutant was obtained. The above *gpdA*(p)::*xlnR*-*hph* overexpression cassette was used to transform the *gpdA*(p)::*clrB*-*ptra* strain protoplasts, and the *gpdA*(p)::*clrB*-*gpdA*(p)::*xlnR* mutant was constructed.

### Construction of the Δ*bgl2*-Δ*creA* and Δ*bgl2-gpdA*(p)::*clrB* mutants

The upstream and downstream fragments of *bgl2* ORF were amplified with the primer pairs Bgl2-F1+Bgl2hph-R and Bgl2hph-F+Bgl2-R1 (S1 Table). Moreover, 1.8 kb of the *hph* cassette was amplified from the plasmid pSilent-1 [55]. The final Δ*bgl2*::*hph* fragment was obtained through the primer pair Bgl2-F2+Bgl2-R2 (S1 Table), with the three fragments above used as a PCR template. The Δ*bgl2*::*hph* cassette was used to transform the protoplasts of the Δ*creA* and *gpdA*(p)::*clrB* mutants, and then the Δ*bgl2*-Δ*creA* and Δ*bgl2-gpdA*(p)::*clrB* mutants were obtained, respectively.

### Construction of *PDE_02864*(p)::*clrB*-*hph* and *gpdA*(p)::*clrB-PDE_02864*(p)::*clrB* mutants

*PDE_02864*(p)::*clrB*-*hph* overexpression cassettes that conferred resistance to hygromycin B was constructed. The *hph* selectable marker cassette was PCR-amplified with the primers Hphs-F and HPH-R1 from the plasmid pSilent-1 [55]. The *PDE_02864* promoter, and *clrB* open reading frame and 3’ untranslated region were amplified with the primer pairs DF1+DClrB-R and ClrB-F+ClrBHPH-R (S1 Table) from the *P*. *oxalicum* genome DNA, respectively. The primer pair DF2+HPH-R1 (S1 Table) was used to produce the final *PDE_02864*(p)::*clrB*-*hph* overexpression cassette via double-joint fusion PCR. The resulting *PDE_02864*(p)::*clrB*-*hph* overexpression cassette was used to transform the wild-type and *gpdA*(p)::*clrB* strain protoplasts, and then the *PDE_02864*(p)::*clrB*-*hph* and *gpdA*(p)::*clrB-PDE_02864*(p)::*clrB* mutants were obtained, respectively.

### Construction of the Δ*amyR*-*gpdA*(p)::*clrB*, Δ*amyR*-Δ*creA*, Δ*amyR*-Δ*bgl2*, Δ*amyR*-Δ*xlnR* mutants

The upstream and downstream flanking fragments of the *amyR* encoding region were amplified with the primer pairs PDE_03964-F1+amyRHph-R and amyRHph-F+PDE_03964-R1 (S1 Table), respectively. The final Δ*amyR*::*hph* fragment was obtained through the nest primer pair PDE_03964-F2+PDE_03964-R2 (S1 Table) with the three fragments (*amyR* flanking sequences and *hph* encoding cassette) used as templates via double-joint PCR. The Δ*amyR*::*hph* cassette was used to transform the *gpdA*(p)::*clrB*-*ptra* protoplasts, and the Δ*amyR*-*gpdA*(p)::*clrB* mutant was obtained. The Δ*amyR*::*ptra* (Δ*PDE_03964*::*ptra*, from TF knock-out cassette set) knockout cassette was used to transform the Δ*bgl2*::*hph*, Δ*creA*::*hph*, and Δ*xlnR*::*hph* mutants, and the Δ*amyR*-Δ*creA*, Δ*amyR*-Δ*bgl2*, Δ*amyR*-Δ*xlnR* mutants were obtained, respectively.

### Construction of the RE-30, RE-27 and RE-29 mutants

First, the Δ*amyR*::*sur* knockout cassette, and *PDE_02864*(p)::*clrB-sur* and *PDE_02864*(p)::*xlnR-sur* overexpression cassettes that conferred resistance to sulfonylurea were constructed. The *sur* selectable marker cassette was PCR-amplified with the primers Sur-F1 and Sur-R1 (S1 Table) from the plasmid pCB1536 [57]. The upstream and downstream flanking fragments for the Δ*amyR-sur* knockout cassette were amplified with the primer pairs PDE_03964-F1+amyRsur-R and amyRsur-F+PDE_03964-R1 (S1 Table), which contained 25 bp homology to the *sur* cassette sequence, respectively. The primer pair PDE_03964-F2+PDE_03964-R2 (S1 Table) was used to produce the final deletion cassette Δ*amyR-sur* through a double-joint fusion PCR. The resulting Δ*amyR-sur* knockout cassette was used to transform the RE-10 (Δ*bgl2*-Δ*creA*-*gpdA*(p)::*clrB*) protoplasts, and the *P*. *oxalicum* and RE-30 (Δ*amyR*-Δ*bgl2*-Δ*creA*-*gpdA*(p)::*clrB*) mutants were obtained. The *clrB* promoter was replaced with the *PDE_02864* (encoding 40S ribosomal protein S8) promoter from *P*. *oxalicum*. The *PDE_02864* promoter sequence was amplified with the primer pair DF1+DP-R. In addition, the *clrB* and *xlnR* open reading frames, and the 3’ untranslated regions were amplified with the primer pairs DClrB-F+ClrBsur-R and DXlnR-F+XlnRSur-R, respectively. The primer pair DF2+Sur-R1 (S1 Table) was used to produce the final *PDE_02864*(p)::*clrB*-*sur* and *PDE_02864*(p)::*xlnR-sur* overexpression cassettes via double-joint fusion PCR. The resulting *PDE_02864*(p)::*clrB-sur* and *PDE_02864*(p)::*xlnR-sur* overexpression cassettes were used to transform the RE-10 protoplasts, and then the RE-27 (Δ*bgl2*-Δ*creA*-*gpdA*(p)::*clrB*-*PDE_02864*(p)::*clrB*) and RE-29 (Δ*bgl2*-Δ*creA*-*gpdA*(p)::*clrB*- *PDE_02864*(p)::*xlnR*) mutants were obtained.

### Southern blot analysis

Fungal genomic DNA was isolated, as described previously [58]. The Primer 5 software was used to identify the appropriate restriction enzymes for the Southern blot analysis of the gene replacement mutants. The fragments used for the probes were amplified with the primers presented in the S1 Table. A DIG-High Prime labeling kit was used to label the knockout cassette flank fragment probes. The homokaryons and integration patterns of the transforming cassettes in the genome were confirmed through Southern blot analysis, as described in the manipulations.

### Phenotypic observations

Colony morphology and conidiation were analyzed after inoculating the Vogel’s medium plates that contained 2% glucose, 2% xylan, 2% starch, or 1% cellulose as sole carbon source or potato dextrose agar (PDA) medium at 30°C for 5 days. The halo sizes that varied among the *P*. *oxalicum* strains were measured on cellulose or starch plates by adding Triton X-100 to a final concentration of 0.5%. Iodine solution (6 g of KI, 0.6 g of I_2_ in 100 mL of H_2_O) was used to indicate visualize starch-degrading colonies through the hydrolysis halo at room temperature for 10 min, which determined if the amylase expression was affected in the *P*. *oxalicum* strain. *P*. *oxalicum* hyphae and conidia were microscopically examined through lactophenol cotton blue staining (0.05 g cotton blue, 20 g phenol crystals, 40 mL glycerol, 20 mL lactic acid, and 20 mL distilled water).

### Enzyme activity and protein concentration assays

Cellulase was produced in a 500 mL flask that contained 100 mL of fluid medium through a two-step cultivation procedure. Strains were first grown at 30°C in 100 mL of medium that contained 2 g of glucose as a carbon source and were then regulated at pH 5.5 and 200 rpm for 20 hours. The cultures were collected through vacuum drum filtration during this second step, and 0.5 g vegetative mycelia was added to 100 mL of Vogel’s medium that contained 2% cellulose as carbon source or wheat bran medium at an initial pH of 5.5 at 30°C and 200 rpm. Culture supernatants (crude enzyme) were diluted with sodium acetate buffer solution (SABF, 0.2 M, pH 4.8). Enzymatic hydrolyses of the polysaccharides were also performed in SABF (0.2 M, pH 4.8). The filter paper enzyme (FPA), endoglucanase (CMCase), xylanase, and amylase activities of the culture supernatants (diluted samples) were assayed using a DNS reagent (10 g 3, 5-dinitrosalicylic acid, 20 g sodium hydroxide, 200 g sodium potassium tartrate, 2.0 g redistilled phenol, and 0.50 g sodium sulfite anhydrous per 1000 mL DNS reagent) against Whatman No. 1 filter paper, carboxymethylcellulose sodium salt (CMC-Na), xylan (from beechwood), and soluble starch. CMC-Na, xylan, or starch was dissolved in SABF to a final concentration of 1% (mass/volume percent, m/v %), and then the mixture was left overnight and was shaken well before using. The following components were added in a 2.0 mL reaction mixture: 0.5 mL diluted culture supernatants and 1.5 mL CMC-Na, xylan, or starch solution for CMCase, xylanase, or amylase activity assays, respectively; and 2.0 mL diluted culture supernatants and 50 mg Whatman No. 1 filter paper for FPA assay into 25 mL colorimetric tube. The mixture was mixed gently and the reaction mixture was incubated for FPA measurement in a 50°C water bath for 1 hour, for CMCase and xylanase activity measurements at 50°C for 30 min, and for amylase activity measurement at 40°C for 10 min. Three milliliters of DNS reagent were then added to stop the reaction. A blank tube (with boiled crude enzyme) was used as control to correct any reducing sugar present in the crude enzyme samples. The tubes were placed in boiling water for 10 min, 20 mL distilled water was added, 200 μL of reaction mixture was pipetted, and the absorbance was determined at 540 nm. The cellobiohydrolase (pNPCase) and β-glucosidase (pNPGase) activities were measured by using 4-Nitrophenyl β-D-cellobioside (pNPC) and 4-Nitrophenyl β-D-glucopyranoside (pNPG) as substrates, respectively. The pNPC or pNPG was dissolved in SABF to a final concentration of 1 mg/mL. Moreover, 50 μL of pNPC solution (containing 1 mg/mL D-Glucono-δ-lactone) or 50 μL of pNPG solution and 100 μL of diluted culture supernatants were mixed, and then the mixtures were incubated in a 50°C water bath for 30 min. The reaction was stopped by adding 0.15 mL of sodium carbonate solution (10%, m/v), then 200 μL of these reaction mixtures was pipetted, and the absorbance was measured at 420 nm. One unit of enzyme activity was defined as the amount of enzyme required to release 1 μmol of glycoside bonds of the substrate per minute under defined assay conditions. Independent triplicate cultures were sampled and analyzed.

The total protein was determined using a Bradford assay kit according to the instructions of the manufacturer.

### Total RNA extraction

Freshly harvested conidia of the wild-type strain or mutants were inoculated with 10^6^ conidia/mL into 100 mL Vogel’s medium that contained 2% glucose, and then grown for 22 hours at 30°C. Mycelia were harvested via vacuum filtration, and then washed with Vogel’s medium without a carbon source, followed by 2 h growth in 100 mL Vogel’s medium without a carbon source. Subsequently, mycelia were harvested via vacuum filtration, and then transferred into Vogel’s medium that contained 2% cellulose for 4, 8, 22, and 46 hours, with 2% glucose for 4 hours, or into a medium without any carbon source for 4 hours. Mycelia were harvested via vacuum filtration, and then immediately finely ground under liquid nitrogen, and then 1 mL of TRIzol reagent was added per 50–100 mg powder. The total RNA was isolated according to the instructions of the manufacturer.

### Northern blot analysis of cellulase gene expression

The total amount (2 μg) of mRNA loaded was normalized by using rRNA as a loading control. The probes used for the Northern blot analysis were the partial cDNAs of *cbh1* (PDE_07945), *eg2* (PDE_09226), and *xyn1* (PDE_08094) that were cloned from the *P*. *oxalicum* genome DNA via PCR with primer pairs (S1 Table). The probes were labeled using a DIG Northern Starter kit, and Northern blot analysis was performed according to the instructions of the manufacturer.

### Quantitative reverse transcription PCR (q-PCR)

Putative targets were validated through q-PCR. Each strain was cultured independently from the Northern blot and RNA-seq experiments. cDNA was synthesized from the total RNA by applying a reagent kit with a gDNA eraser according to the instructions of the manufacturer. The obtained cDNA was applied for quantitative reverse transcription-PCR experiments. All q-PCR amplification was performed in 20 μl total volume with 7.4 μl distilled water, 0.8 μl of each primer (10 mM), 10 μl SYBR Premix Ex TaqII, and 1 μl template cDNA through the following program [59]: 95°C for 2 min, 40 cycles at 95°C for 10 sec, and 30 s at 61°C. The fluorescence signal was measured at the end of each extension step at 80°C. A melting curve program with a temperature gradient of 0.1°C per second from 65°C to 95°C was performed. The corresponding primers are shown in the S1 Table. The quantity and copy number of each target gene were calculated using a standard curve. Six 10-fold serial dilutions of purified DNA template (0.5 kb–1.0 kb) were prepared for the target genes to determine the standard curve of each target gene. The correlation coefficient (R2) for each standard curve was verified to be 0.99 or greater. The transcriptional expression of transcription factor genes were measured by q-PCR, and their expression levels were normalized to wild-type. Gene expression levels for cellulase genes were measured by q-PCR using actin (PDE_01092) as a control and normalized to expression levels by actin values/10000. Three biological replicates were performed on the same 96-well plate by using cultures grown in parallel. Data processing and statistical analyses were performed using Microsoft Excel.

### RNA sequencing and transcription expression analysis

A cDNA library prepared from mRNA was organized according to standard protocols. Quality control was implemented using the Real-Time PCR Systems. All the cDNA libraries were sequenced on the Illumina platform. Sequenced reads were mapped against predicted transcripts from the *P*. *oxalicum* 114–2 genome using the SOAP2 software for short oligonucleotide alignment (http://soap.genomics.org.cn/soapaligner.html) [60]. Transcript abundance (Reads Per Kb per Million reads, RPKM) [61] was estimated with RPKM = [# of mapped reads]/([length of transcript]/1000)/([total reads]/10^6). The differential gene expression was analyzed using DESeq software package [62] and NOIseq v2.10 (http://www.bioconductor.org/packages/release/bioc/html/NOISeq.html) with |log2(fold change)|>1 and probability≥0.8 as thresholds [63]. The biological replicates used for RNA-seq were highly reproducible. These datasets (RPKM) were subjected to hierarchical cluster analysis using the software HCE3.5 (http://www.cs.umd.edu/hcil/hce/) to determine the groups of genes with similar expression patterns for a different group of regulons. The Blast2go software v3.0 was used for the gene ontology analyses (https://www.blast2go.com/) [64]. Secreted proteins were predicted using SignalP 4.1 (http://www.cbs.dtu.dk/services/SignalP/). Secondary metabolism gene clusters were identified using annotated proteins in *P*. *oxalicum* [24]. Protein sequence alignments were performed among the *P*. *oxalicum*, *T*. *reesei*, and *N*. *crassa* proteomes using the BioEdit Sequence Alignment Editor software (http://www.mbio.ncsu.edu/BioEdit/bioedit.html).

### Secretome analysis using label-free LC-MS/MS

Culture supernatants were collected after shifting to cellulose for 96 hours by filtrating using 0.22 μm PES membrane, and then the supernatants were desalted with 10 kDa molecular cut-off membrane, and were precipitated by acetone and trichloroacetic acid (20:1). The obtained protein powders were dissolved in denaturation buffer (0.5 M Tris-HCL, 2.75 mM EDTA, 6 M Guanadine-HCL), and were then reduced using 1 M DTT at 37°C for 1 hour. The following alkylation was performed using iodoacetamide for 2 hours away from light, and the samples were desalted and collected using a Microcon YM-10 Centrifugal Filter Unit. The obtained protein samples were digested thoroughly using trypsin for 12 hours, and these peptide mixtures were desalted with a ZipTip C18 column. These collected secretome samples were further separated on a C18-reversed phase column and then directly mounted on the electrospray ion source of a mass spectrometer. The peptides were subjected to nanoelectrospray ionization, followed by tandem mass spectrometry (MS/MS) in an LTQ Orbitrap Velos Pro coupled with high-performance liquid chromatography. Intact peptides were detected in the Orbitrap at 60000 resolution. Peptides were selected for MS/MS using a collision-induced dissociation operating mode with 35% normalized collision energy setting. Ion fragments were detected in the LTQ Orbitrap. A data-dependent procedure that alternated between one MS scan, followed by 10 MS/MS scans, was applied for the 10 most abundant precursor ions above the 5000 threshold ion count in the MS survey scan with the following Dynamic Exclusion settings: 2 repeat counts, 30 s repeat duration, and 120 s exclusion duration. An electrospray voltage of 2.2 kV was applied. For the MS scans, the m/z scan range was 350 Da to 1800 Da. Mass spectrometry data processing was performed using the Mass-Lynx software (version 4.1, Waters). LC-MS/MS analysis data were identified by searching the *P*. *oxalicum* protein database (http://genome.jgi.doe.gov/Penox1/Penox1.home.html).

### Electrophoretic mobility shift assay

The DNA-binding domain of ClrB (1–163 amino acids) was PCR-amplified from the *P*. *oxalicum* 114–2 genome DNA using the primers ClrB-RTF and ClrB-RTR (S1 Table). The resulting amplicon was digested using *Eco*RI and *Bam*HI, and then inserted into the expression vector pGEX-4T-1 at the GST downstream with corresponding restriction sites. The correct fusion plasmid was confirmed via nucleotide sequencing, and then transformed into *E*. *coli* BL21 (DE3). Parental and recombinant strains were cultured in a lysogeny broth medium and were induced by 0.05 mM isopropyl-β-D-thiogalactopyranoside (IPTG) at 30°C and 150 rpm for 8 hours to induce the GST alone and the GST-ClrB binding-domain production. Protein purification was performed by using Glutathione Sepharose 4B beads after cell ultrasonic decomposition according to the product manual. The protein concentration was then measured using a Bradford Kit according to the instructions of the manufacturer. A 2 kb promoter of PDE_07945 was then amplified from the *P*. *oxalicum* 114–2 genome DNA using the primers PDE_07945-F and PDE_07945-R (S1 Table), and then purified using a gel extraction kit according to the instructions of the manufacturer. The DNA concentration was determined using a UV-Vis Spectrophotometer Q5000. GST alone or GST-ClrB binding-domain was mixed in the binding buffer (containing 100 mM Tris-HCl, 100 mM KCl, 10 mM EDTA, 2.5 mM DTT, and 20% Ficoll-400, supplementing 1 μg Poly(dI-dC) to avoid unspecific binding) with purified DNA (20 ng) at room temperature for 10 min for electrophoretic mobility shift assay. The protein-DNA mix was then separated via gel electrophoresis, stained by ethidium bromide, and then visualized. The protein and DNA complex were retardant relative to free DNA. Binding reaction was performed by gradient increasing the protein content to avoid artificial results. GST alone was used as negative control.

### Yeast two-hybrid system (Y2H)

The Matchmaker GAL4 two-hybrid system 3 was used for yeast two-hybrid assays. Full-length ORFs of the transcription factors ClrB, CreA, AmyR, and XlnR were PCR-amplified using the *P*. *oxalicum* 114–2 cDNA as templates (with primers listed in S1 Table). All the amplicons were cloned into the plasmid pGAD-T7 with the corresponding restriction sites, leading to AD-ClrB, AD-CreA, AD-XlnR, and AD-AmyR. Similarly, the full-length *creA*, *amyR*, and *xlnR* were cloned into the partner plasmid pGBK-T7, which generated BD-CreA, BD-AmyR, and BD-XlnR, respectively. All the fused plasmids were confirmed via nucleotide sequencing. The plasmids pGAD-T7 or AD-X coupling with pGBK-T7 or BD-X in pair were co-transformed into *S*. *cerevisiae* AH109 and were cultivated on an SD medium without Trp and Leu for 3 days at 30°C. Protein–protein interaction assay was performed on SD plates without Leu, Trp, and His, and on YPD plates to avoid artificial results. pGBKT7-53/pGADT7-T and pGBKT7-Lam/pGADT7-T pairs were used as internal positive and negative controls, respectively. Moreover, AD-ClrB, AD-CreA, AD-xlnR, and AD-AmyR paired with an empty pGBK-T7 introduced into AH109 were used to eliminate false positive results.

The RNA-seq data have been deposited in NCBI's Gene Expression Omnibus with accession number GSE69298 (http://www.ncbi.nlm.nih.gov/geo/query/acc.cgi?acc=GSE69298).

## Supporting Information

S1 Fig Genomic DNA from putative transformants was analyzed by Southern blot. Genomic DNA samples from putative transformants Δ*clrB* (A), Δ*amyR* (B), Δ*PDE_02584* (C), Δ*PDE_09881* (D), Δ*PDE_07134* (E), Δ*PDE_04095* (F), Δ*PDE_08372* (G), Δ*PDE_08462* (H), Δ*PDE_00988* (I), and Δ*PDE_03268* (J) mutants were analyzed by Southern blot.
(TIF)

S2 Fig The three biological replicates for RNA-seq from wild-type and Δ*clrB* strains for RNA-seq data showed a high Pearson correlation. The three biological replicates for RNA-seq from wild-type strain when grown on medium containing no carbon (A), 2% glucose (B) or 2% cellulose (C) for 4 hours. (D) The three biological replicates for RNA-seq from Δ*clrB* strain when grown on cellulose for 4 hours.
(TIF)

S3 Fig Enzyme activities of culture supernatants from mutants containing double overexpression of *clrB* on cellulose. The FPA (A), xylanase activity (B), pNPCase activity (C), and pNPGase activity (D) from *gpdA*(p)::*clrB*, *PDE_02864*(p)::*clrB* and *gpdA*(p)::*clrB*-*PDE_02864*(p)::*clrB* mutants versus the wild-type strain were separately evaluated when grown on cellulose in flasks.
(TIF)

S4 Fig Phenotypic observation for Δ*creA*-Δ*clrB* mutant. The Δ*creA*-Δ*clrB* strain produced visible halo in the cellulose medium plate in contrast to the Δ*clrB* mutant when cultured for 9 days on 1% cellulose plate.
(TIF)

S5 Fig Transcription expression of β-glucosidase genes in *P*. *oxalicum clrB* and/or *creA* mutants on cellulose. (TIF)

S6 Fig The three biological replicates for RNA-seq from Δ*clrB*, Δ*creA*-Δ*clrB*, Δ*creA*-*gpdA*(p)::*clrB* and Δ*amyR* strains showed a high Pearson correlation. The three biological replicates for RNA-seq from Δ*clrB* (A), Δ*creA*-Δ*clrB* (B), Δ*creA*-*gpdA*(p)::*clrB* (C), and Δ*amyR* (D) mutants when grown on cellulose for 4 hours.
(TIF)

S7 Fig Lack of Bgl2 and overexpression of ClrB or lack of CreA induced cellulase expression. (A, B) The transcription levels for endoglucanase gene (*eg*) and β-glucosidase genes (*bgl*) were determined in the mutants versus wild-type strain on cellulose by q-PCR. (C) The transcription levels for endoglucanase gene (*eg*) were determined in the mutants versus wild-type strain under carbon-free conditions for 4 hours by q-PCR.
(TIF)

S8 Fig Lack of Bgl2 and overexpression of ClrB or lack of CreA synergistically enhanced pNPCase activity (A) and CMCase activity (B) under no carbon conditions. (TIF)

S9 Fig Improvement of *P*. *oxalicum* cellulase production via reconstruction of expression regulation network (RERN). The FPA (A), pNPCase activity (B), pNPGase activity (C), xylanase activity (D) for the wild-type, triple-mutant RE-10 and quadruple-mutants RE-27 and RE-29 were separately evaluated when grown on cellulose in flasks. (E, F) SDS-PAGE of proteins from unconcentrated culture supernatants from wild-type, RE-10, RE-27 and RE-29 strains when cultured on glucose for 22 hours then shifted to cellulose medium or wheat bran media for 96 hours. Sixteen (E) and eight (F) microliters of supernatants were loaded, respectively.
(TIF)

S10 Fig The effect on cellulase expression by lack of AmyR in Δ*bgl2* and RE-10 mutants, respectively. (A) q-PCR measurements of three *cbh* gene expression in Δ*amyR*-Δ*bgl2* mutant versus wild-type and each single mutation strains when grown on cellulose for 4 hours. Gene expression levels were normalized to values/10000 of actin gene expression as a control. (B) The transcription levels for *cbh1*, *clrB* and *xlnR* in the RE-10 (Δ*bgl2-*Δ*creA*-*gpdA*(p)::*clrB*) and RE-30 (Δ*amyR*-Δ*bgl2*-Δ*creA*-*gpdA*(p)::*clrB*) mutants versus wild-type strain under cellulose growth conditions by q-PCR. (C, D) The pNPCase activity and amylase activity for the RE-10 and RE-30 mutants were separately evaluated when grown on cellulose in flasks. Expression levels were normalized to the wild-type.
(TIF)

S11 Fig Hierarchical clustering of RPKM for *amyR* regulon genes for 579 decreased expression on cellulose. (TIF)

S1 Table Primers used in this study. (XLSX)

S2 Table Gene list of the 103 genes showed decrease in Δ*clrB* as compared to wild-type on cellulose. (XLSX)

S3 Table Gene list of the 121 genes showed increase in Δ*clrB* as compared to wild-type on cellulose. (XLSX)

S4 Table Gene list of the 155 cellulose-regulon genes showed increase in wild-type on cellulose. (XLSX)

S5 Table Gene list of the 117 cellulose-regulon genes showed decrease in wild-type on cellulose. (XLSX)

S6 Table Identification and quantification of predicted secretory proteins in the supernatants from wild-type strain and Δ*clrB*, *gpdA*(p)::*clrB*, Δ*creA*, *gpdA*(p)::*creA*, and Δ*creA-gpdA*(p)::*clrB* mutants on cellulose for 96 hours. (XLSX)

S7 Table Gene list of the 131 genes showed increase in Δ*amyR* as compared to wild-type on cellulose. (XLSX)

S8 Table Gene list of the 579 genes showed decrease in Δ*amyR* as compared to wild-type on cellulose. (XLSX)

S9 Table Strains used in this study. (XLSX)
