# Supplementary figures and images for: Synergistic and Dose-Controlled Regulation of Cellulase Gene Expression in Penicillium oxalicum
Source: PLoS Genet. 2015 Sep 11;11(9):e1005509. doi: 10.1371/journal.pgen.1005509 (PMC4567317; doi:10.1371/journal.pgen.1005509)

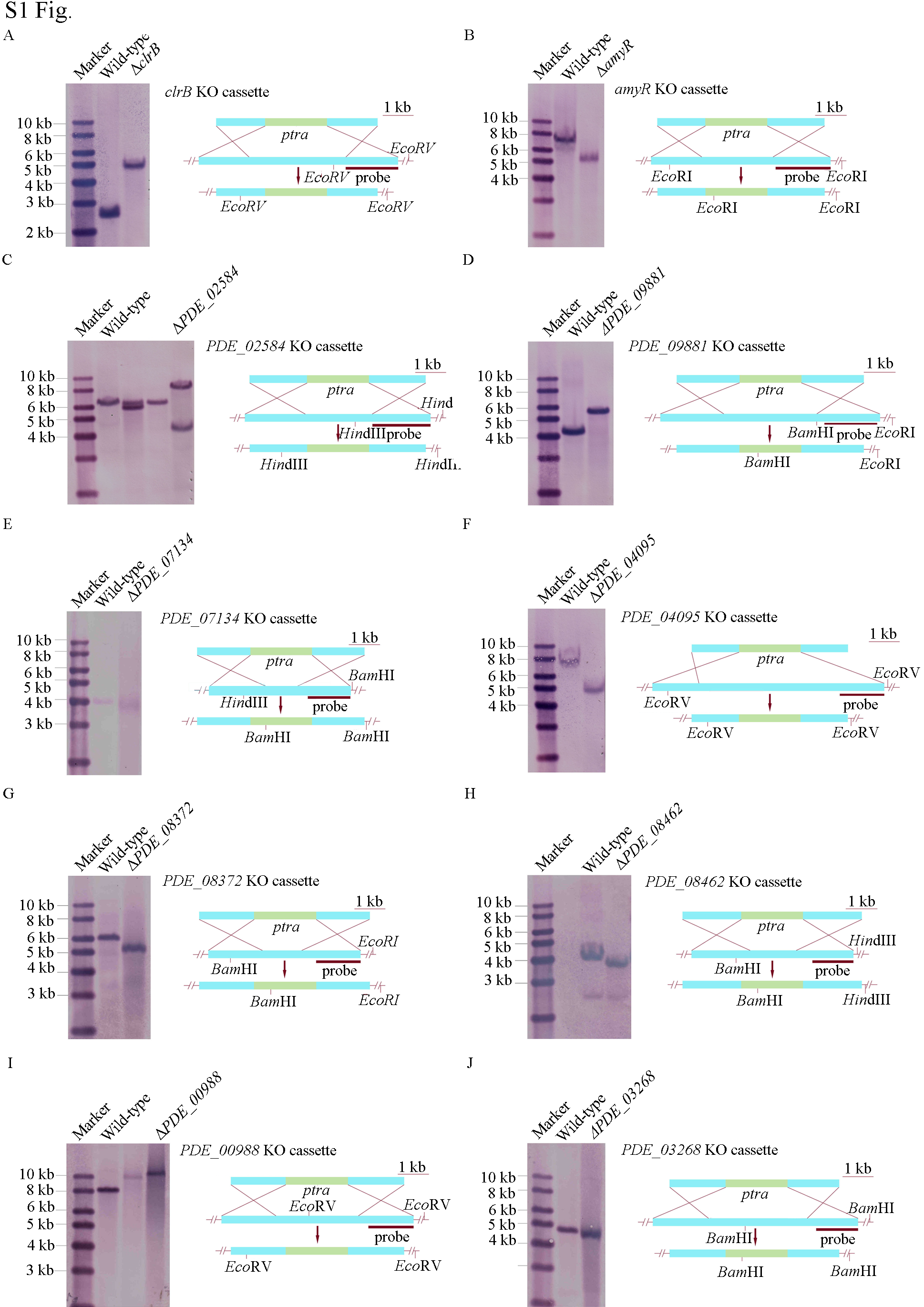

Supplement: S1 Fig — Genomic DNA samples from putative transformants ΔclrB (A), ΔamyR (B), ΔPDE_02584 (C), ΔPDE_09881 (D), ΔPDE_07134 (E), ΔPDE_04095 (F), ΔPDE_08372 (G), ΔPDE_08462 (H), ΔPDE_00988 (I), and ΔPDE_03268 (J) mutants were analyzed by Southern blot. (TIF) [file pgen.1005509.s001.tif]

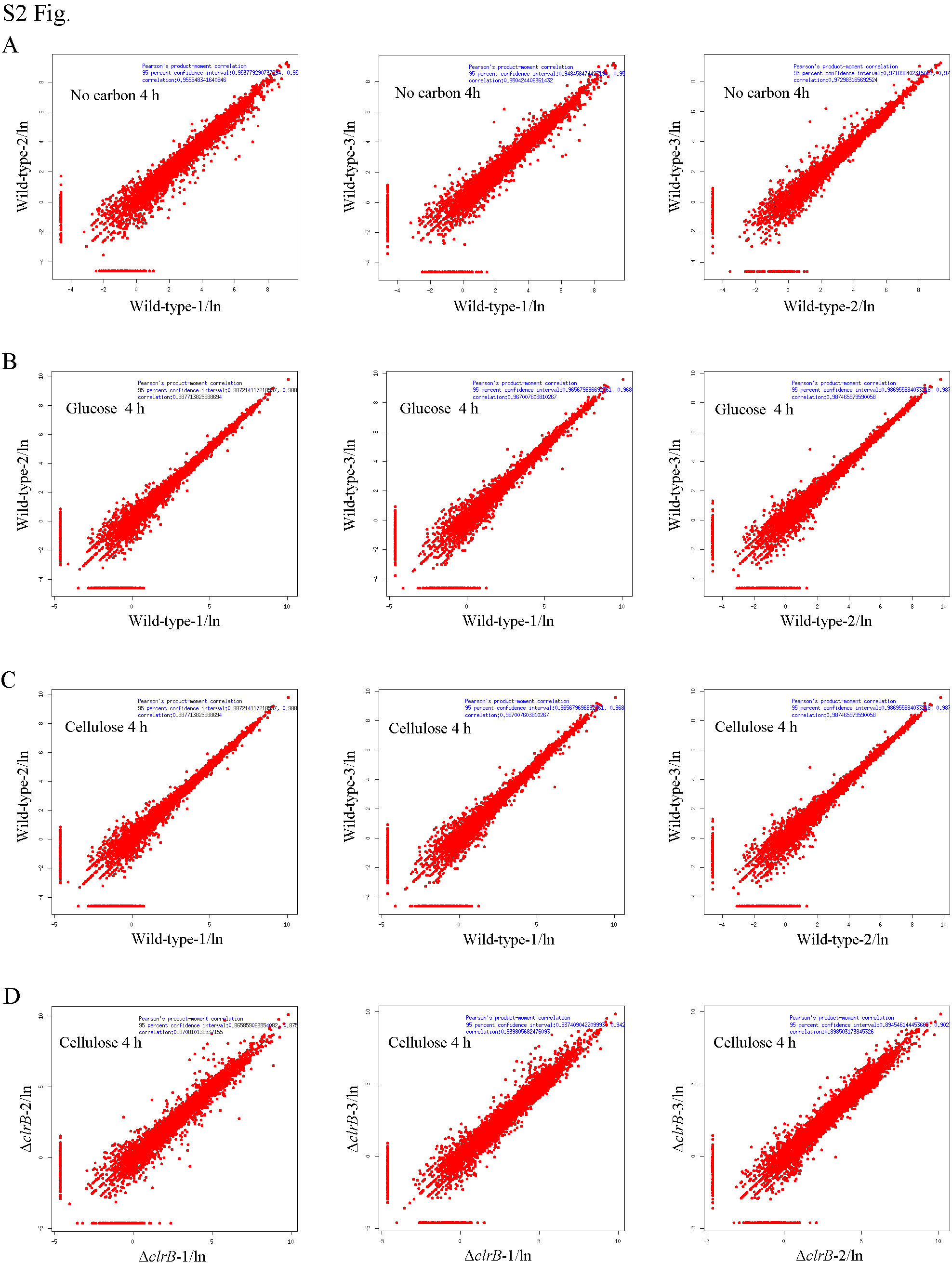

Supplement: S2 Fig — The three biological replicates for RNA-seq from wild-type strain when grown on medium containing no carbon (A), 2% glucose (B) or 2% cellulose (C) for 4 hours. (D) The three biological replicates for RNA-seq from ΔclrB strain when grown on cellulose for 4 hours. (TIF) [file pgen.1005509.s002.tif]

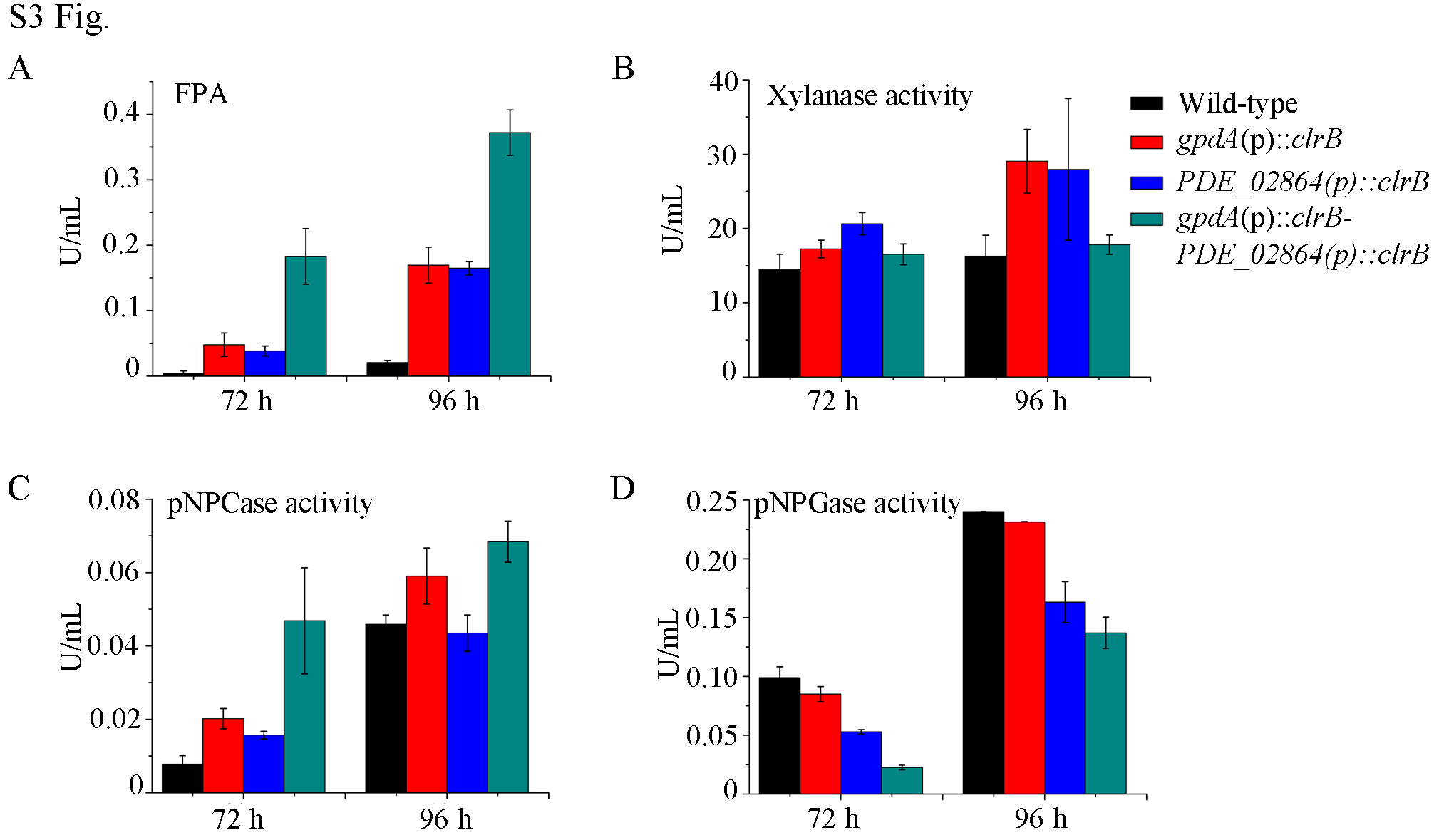

Supplement: S3 Fig — The FPA (A), xylanase activity (B), pNPCase activity (C), and pNPGase activity (D) from gpdA(p)::clrB, PDE_02864(p)::clrB and gpdA(p)::clrB-PDE_02864(p)::clrB mutants versus the wild-type strain were separately evaluated when grown on cellulose in flasks. (TIF) [file pgen.1005509.s003.tif]

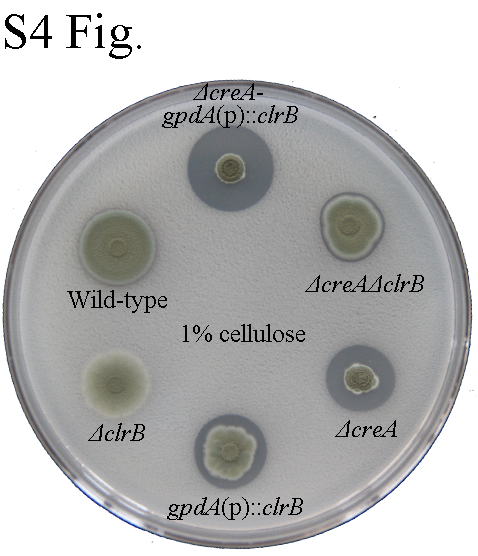

Supplement: S4 Fig — The ΔcreA-ΔclrB strain produced visible halo in the cellulose medium plate in contrast to the ΔclrB mutant when cultured for 9 days on 1% cellulose plate. (TIF) [file pgen.1005509.s004.tif]

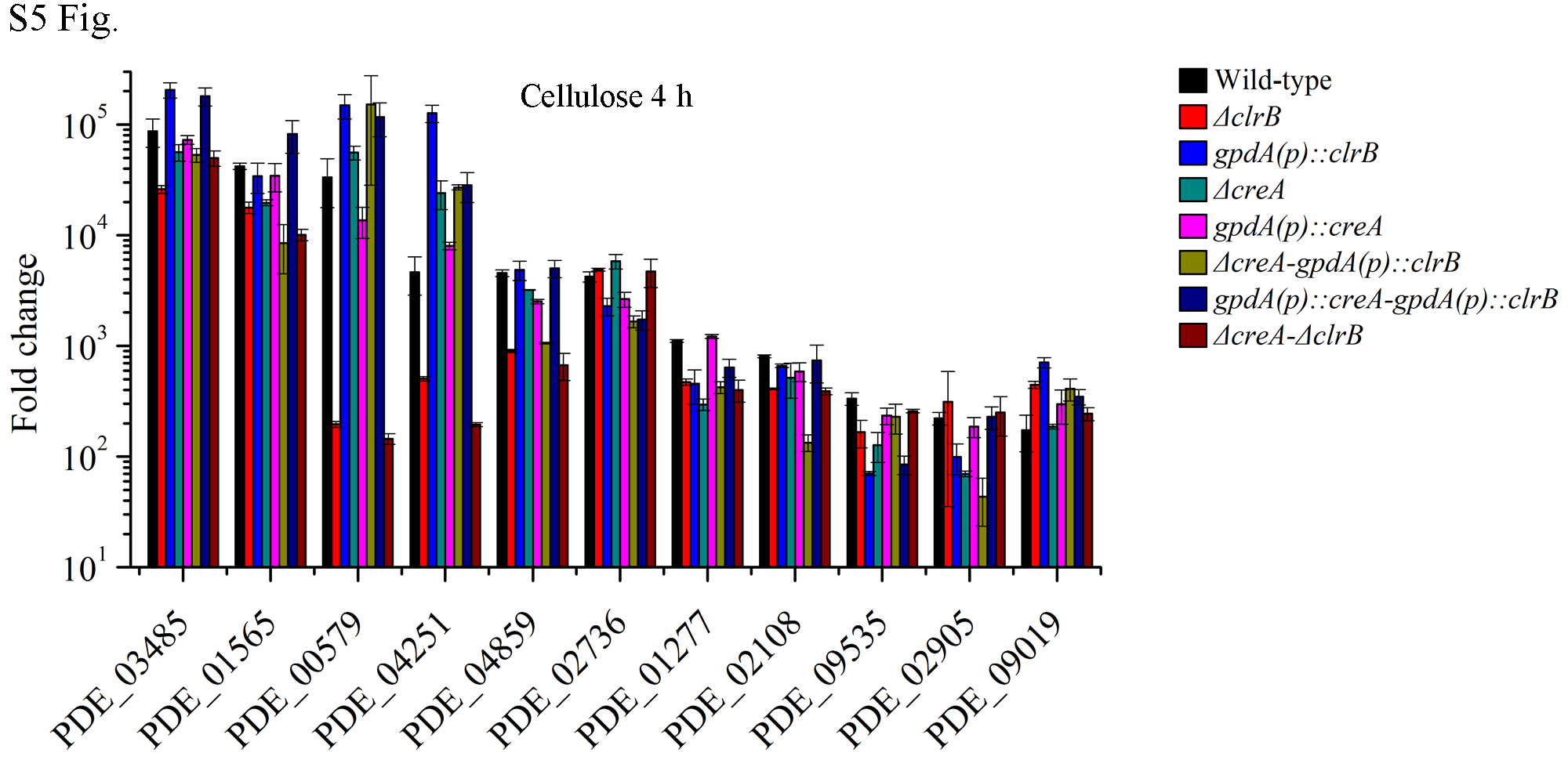

Supplement: S5 Fig — (TIF) [file pgen.1005509.s005.tif]

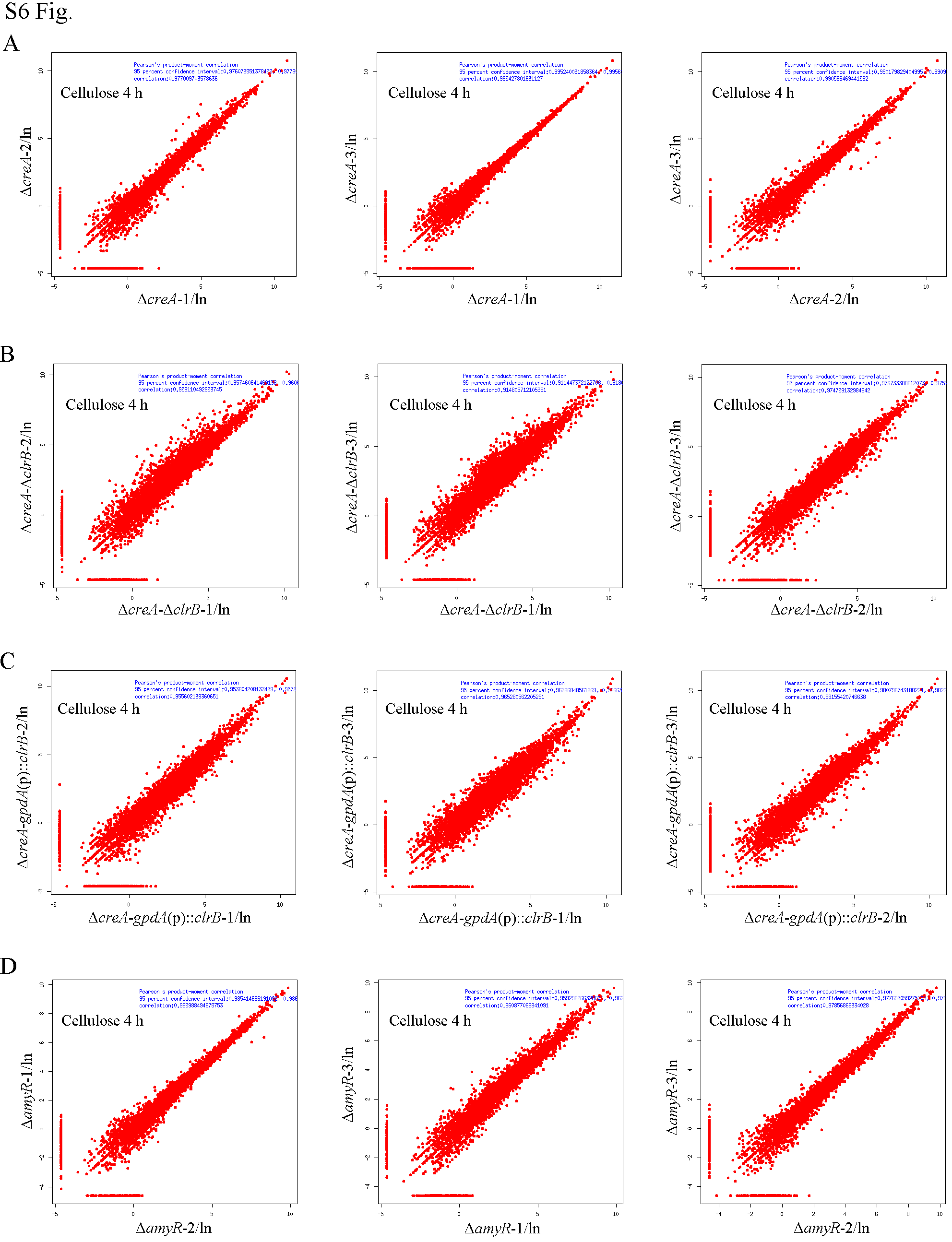

Supplement: S6 Fig — The three biological replicates for RNA-seq from ΔclrB (A), ΔcreA-ΔclrB (B), ΔcreA-gpdA(p)::clrB (C), and ΔamyR (D) mutants when grown on cellulose for 4 hours. (TIF) [file pgen.1005509.s006.tif]

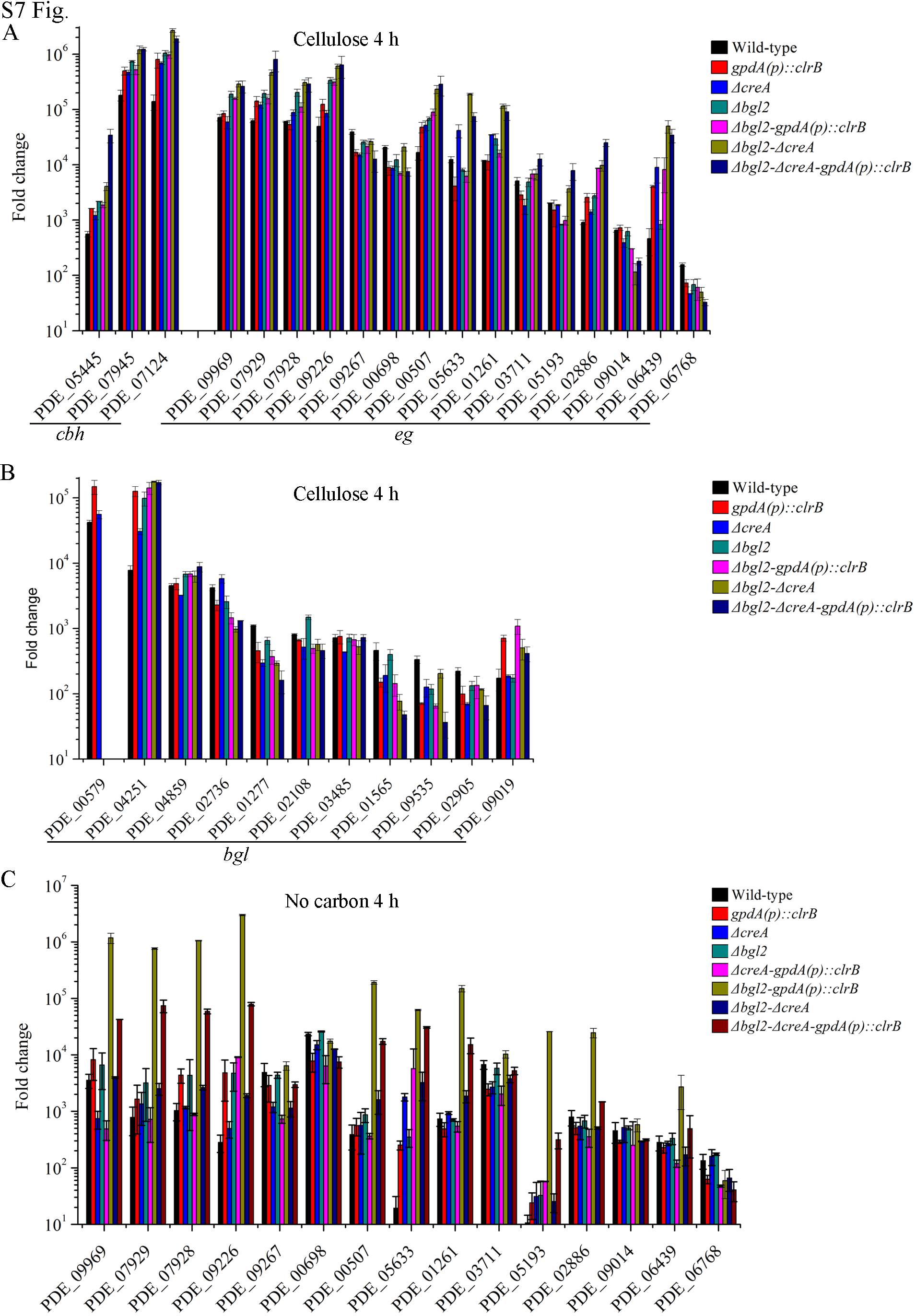

Supplement: S7 Fig — (A, B) The transcription levels for endoglucanase gene (eg) and β-glucosidase genes (bgl) were determined in the mutants versus wild-type strain on cellulose by q-PCR. (C) The transcription levels for endoglucanase gene (eg) were determined in the mutants versus wild-type strain under carbon-free conditions for 4 hours by q-PCR. (TIF) [file pgen.1005509.s007.tif]

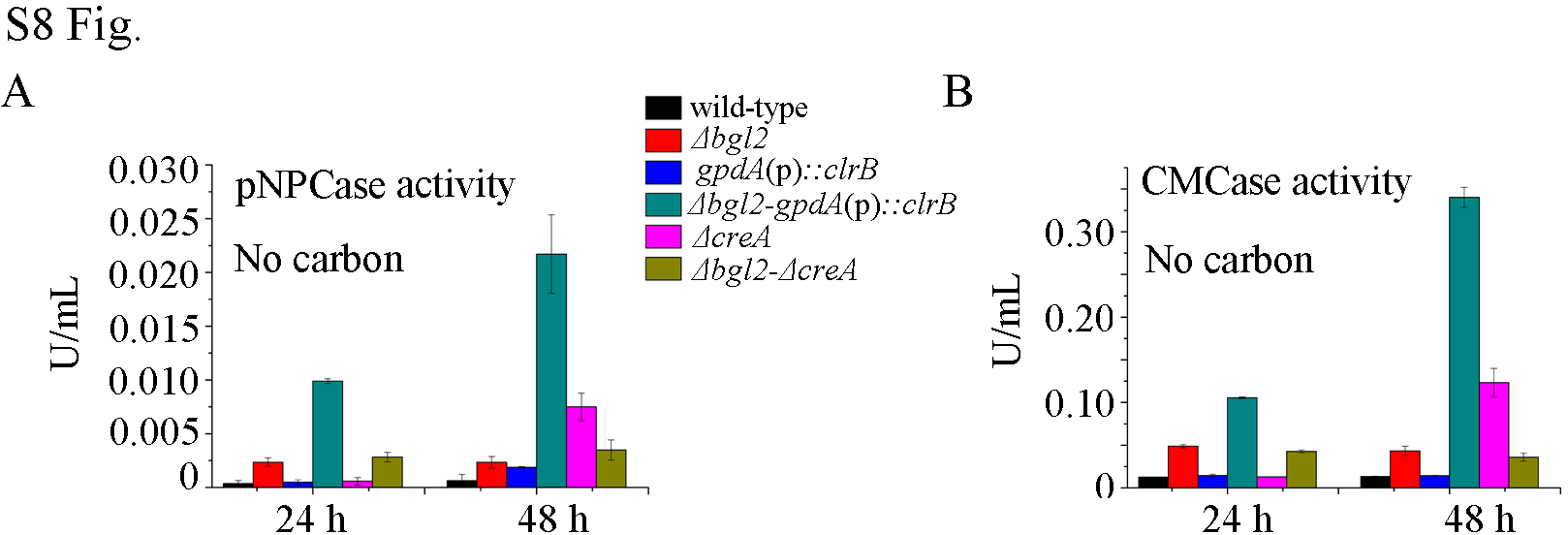

Supplement: S8 Fig — (TIF) [file pgen.1005509.s008.tif]

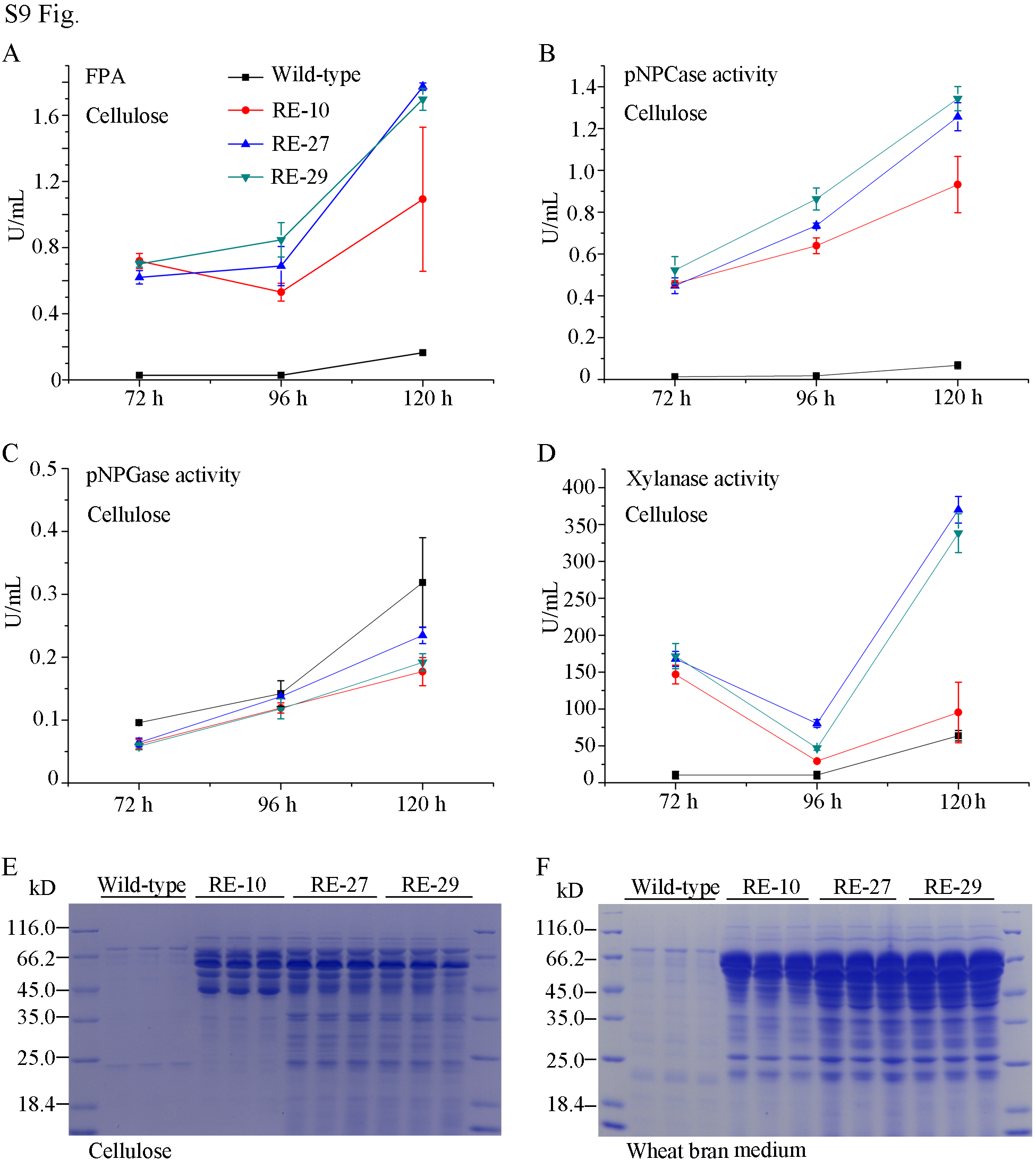

Supplement: S9 Fig — The FPA (A), pNPCase activity (B), pNPGase activity (C), xylanase activity (D) for the wild-type, triple-mutant RE-10 and quadruple-mutants RE-27 and RE-29 were separately evaluated when grown on cellulose in flasks. (E, F) SDS-PAGE of proteins from unconcentrated culture supernatants from wild-type, RE-10, RE-27 and RE-29 strains when cultured on glucose for 22 hours then shifted to cellulose medium or wheat bran media for 96 hours. Sixteen (E) and eight (F) microliters of supernatants were loaded, respectively. (TIF) [file pgen.1005509.s009.tif]

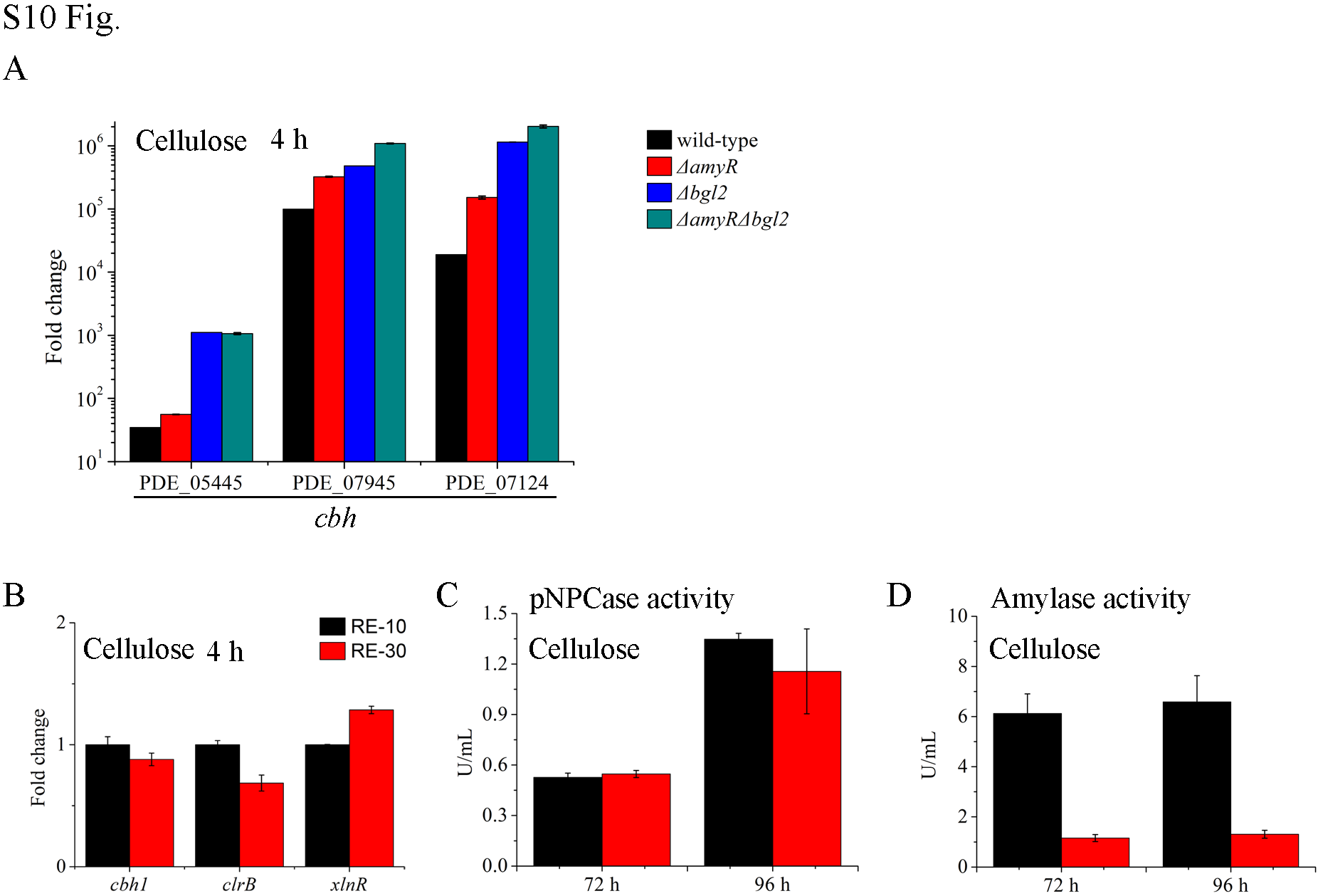

Supplement: S10 Fig — (A) q-PCR measurements of three cbh gene expression in ΔamyR-Δbgl2 mutant versus wild-type and each single mutation strains when grown on cellulose for 4 hours. Gene expression levels were normalized to values/10000 of actin gene expression as a control. (B) The transcription levels for cbh1, clrB and xlnR in the RE-10 (Δbgl2-ΔcreA-gpdA(p)::clrB) and RE-30 (ΔamyR-Δbgl2-ΔcreA-gpdA(p)::clrB) mutants versus wild-type strain under cellulose growth conditions by q-PCR. (C, D) The pNPCase activity and amylase activity for the RE-10 and RE-30 mutants were separately evaluated when grown on cellulose in flasks. Expression levels were normalized to the wild-type. (TIF) [file pgen.1005509.s010.tif]

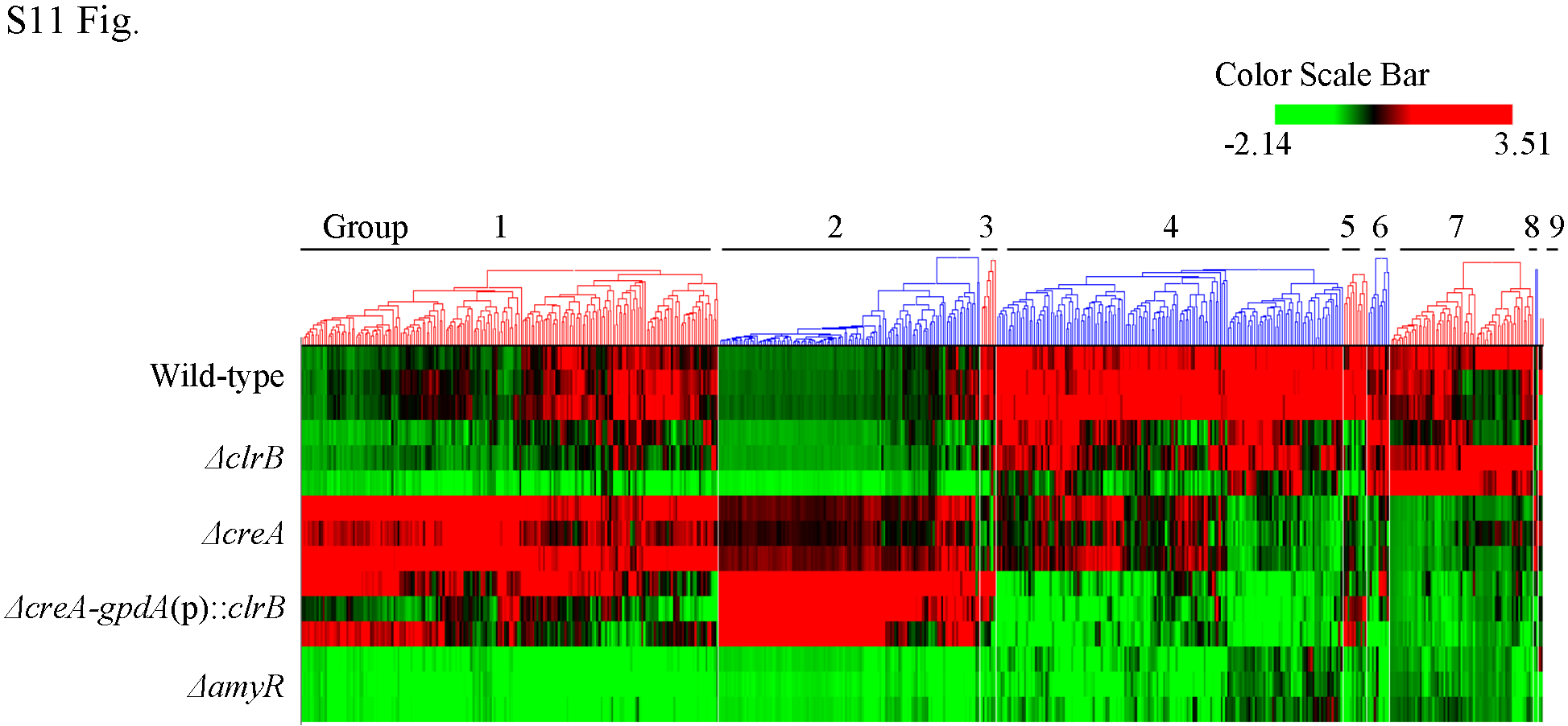

Supplement: S11 Fig — (TIF) [file pgen.1005509.s011.tif]
